# Preterm birth as a determinant of neurodevelopment and cognition in children (PRENCOG): protocol for an exposure-based cohort study in the UK

**DOI:** 10.1136/bmjopen-2024-085365

**Published:** 2024-09-16

**Authors:** James P Boardman, Ruth Andrew, Mark E Bastin, Cheryl Battersby, G David Batty, Manuel Blesa Cábez, Simon R Cox, Jill Hall, Lauren Ingledow, Riccardo E Marioni, Neena Modi, Lee Murphy, Alan J Quigley, Rebecca M Reynolds, Hilary Richardson, Sarah J Stock, Michael J Thrippleton, Athanasios Tsanas, Heather C Whalley

**Affiliations:** 1 Centre for Reproductive Health, University of Edinburgh, Edinburgh, UK; 2 Centre for Clinical Brain Sciences, University of Edinburgh, Edinburgh, UK; 3 Centre for Cardiovascular Science, University of Edinburgh, Edinburgh, UK; 4 Section of Neonatal Medicine, Imperial College London, London, UK; 5 Department of Epidemiology and Public Health, University College London, London, UK; 6 Lothian Birth Cohorts, Department of Psychology, The University of Edinburgh, Edinburgh, UK; 7 Adult Preemie Advocacy Network, London, UK; 8 Centre for Genomic and Experimental Medicine, University of Edinburgh, Edinburgh, UK; 9 Edinburgh Clinical Research Facility, University of Edinburgh, Edinburgh, UK; 10 Department of Radiology, NHS Lothian, Edinburgh, UK; 11 School of Philosophy, Psychology, and Language Sciences, University of Edinburgh, Edinburgh, UK; 12 Usher Institute, Edinburgh Medical School, University of Edinburgh, Edinburgh, UK; 13 The Alan Turing Institute, London, UK

**Keywords:** Neonatal intensive & critical care, Magnetic Resonance Imaging, Machine Learning, Developmental neurology & neurodisability, Physiological Stress, Immunity

## Abstract

**Abstract:**

**Introduction:**

Preterm birth (PTB) is strongly associated with encephalopathy of prematurity (EoP) and neurocognitive impairment. The biological axes linking PTB with atypical brain development are uncertain. We aim to elucidate the roles of neuroendocrine stress activation and immune dysregulation in linking PTB with EoP.

**Methods and analysis:**

PRENCOG (PREterm birth as a determinant of Neurodevelopment and COGnition in children: mechanisms and causal evidence) is an exposure-based cohort study at the University of Edinburgh. Three hundred mother–infant dyads comprising 200 preterm births (gestational age, GA <32 weeks, exposed) and 100 term births (GA >37 weeks, non-exposed), will be recruited between January 2023 and December 2027. We will collect parental and infant medical, demographic, socioeconomic characteristics and biological data which include placental tissue, umbilical cord blood, maternal and infant hair, infant saliva, infant dried blood spots, faecal material, and structural and diffusion MRI. Infant biosamples will be collected between birth and 44 weeks GA.

EoP will be characterised by MRI using morphometric similarity networks (MSNs), hierarchical complexity (HC) and magnetisation transfer saturation imaging (MTsat). We will conduct: first, multivariable regressions and statistical association assessments to test how PTB-associated risk factors (PTB-RFs) relate to MSNs, HC and or MTsat; second, structural equation modelling to investigate neuroendocrine stress activation and immune dysregulation as mediators of PTB-RFs on features of EoP. PTB-RF selection will be informed by the variables that predict real-world educational outcomes, ascertained by linking the UK National Neonatal Research Database with the National Pupil Database.

**Ethics and dissemination:**

A favourable ethical opinion has been given by the South East Scotland Research Ethics Committee 02 (23/SS/0067) and NHS Lothian Research and Development (2023/0150). Results will be reported to the Medical Research Council, in scientific media, via stakeholder partners and on a website in accessible language (https://www.ed.ac.uk/centre-reproductive-health/prencog).

**STRENGTHS AND LIMITATIONS OF THIS STUDY**

- The PRENCOG study includes a new cohort of neonates enriched for preterm birth (PTB) with detailed phenotyping of the hypothalamic–pituitary–adrenal axis, the epigenome, neuroanatomy (brain MRI), the social graph, demographic and medical characteristics, consent for longer-term follow-up.
- PRENCOG will determine the weighted contributions of multidimensional PTB-associated risk factors (PTB-RFs) to neurodevelopmental outcomes and real-world educational performance of children born preterm by linking the UK National Neonatal Research Database and the National Pupil Database.
- Neuroinformatic approaches will identify the biological axes that embed important PTB-RFs in child brain development and determine targets within neuroendocrine stress and immune pathways that lead to atypical brain development.
- Parents and survivors of PTB are involved in designing, delivering and disseminating the PRENCOG study and have co-created participant-facing study materials.
- A limitation is that PRENCOG is in a high-income setting, so the generalisability of results to low and middle income country (LMIC) settings is uncertain.

## Introduction

### Background

 Globally, preterm birth (PTB) is estimated to affect 13.4 million pregnancies per annum.^1^ Over the past two decades, the survival rate of children born preterm has improved due to advances in perinatal medicine, but outcomes remain challenging: 10%–15% of children born very preterm (<32 weeks) develop cerebral palsy, 30%–50% develop an intellectual disability, and this population is at increased risk of problems with socialisation, behaviour, language, low educational attainment, autism and attention deficit hyperactivity disorder.^2^ Adults who were born preterm are more likely to experience a mood disorder, age-related cognitive impairment, schizophrenia and cardiometabolic disease.^3^ PTB accounts for one of the highest numbers of disability-adjusted life-years of any single childhood condition.^4^ There are no effective treatments for improving brain health after PTB, which brings into sharp focus the need to identify protective factors and intervention targets.

The neurobiological basis for adverse neurological, cognitive and psychiatric outcomes following PTB is related to cerebral white matter injury and subsequent dysmaturational processes in white matter and neuroaxonal structures collectively termed the ‘encephalopathy of prematurity’ (EoP).^2^ MRI is sensitive to features of EoP and so has become an important assessment modality for investigating determinants of brain health in preterm infants.^5 6^

**Figure 1:**
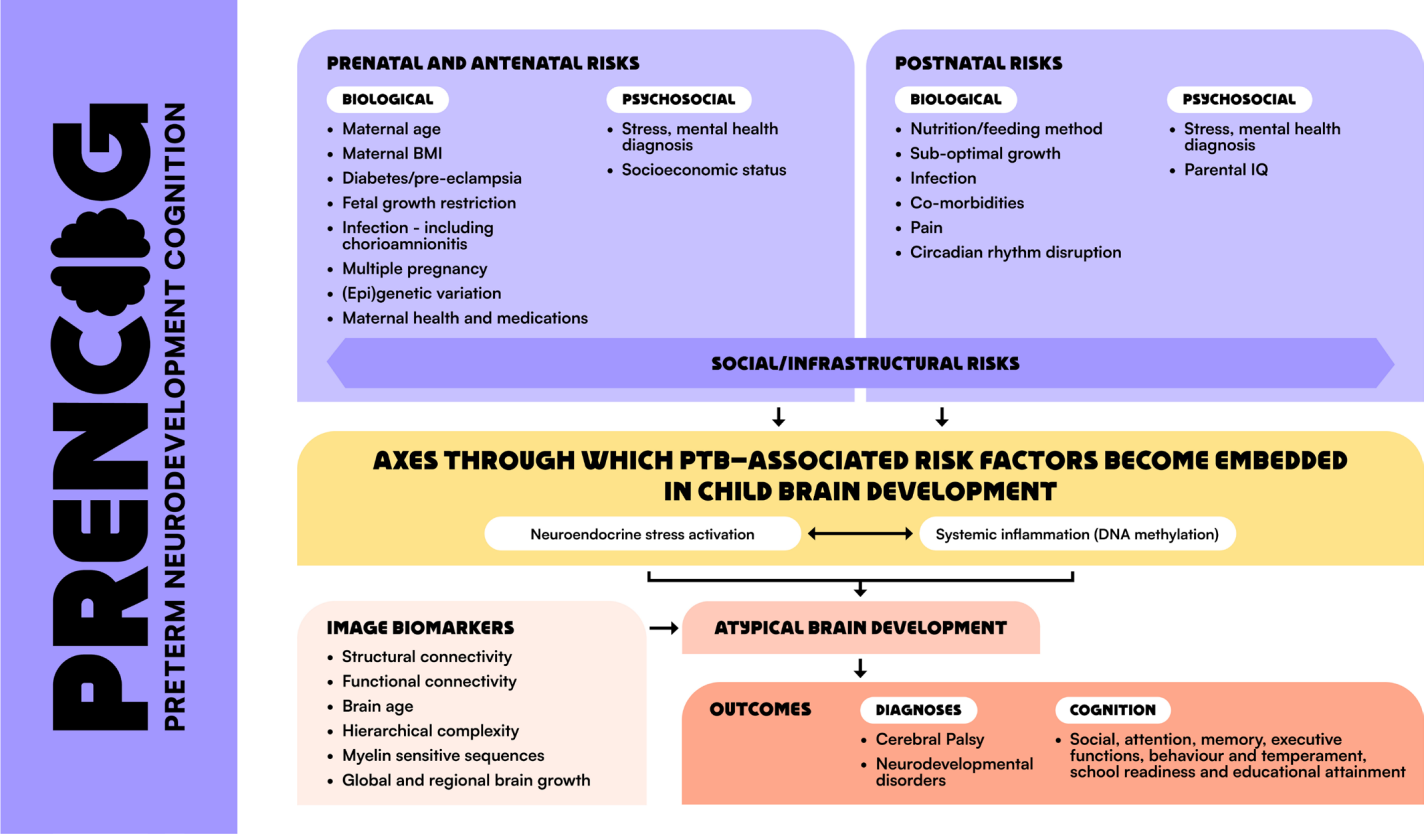
PTB-RFs linked with altered cognition in children, proposed biological pathways that transmit risk to atypical brain development/outcome, and image biomarkers for delineating upstream pathways and predicting risk and resilience. BMI, body mass index; PTB-RFs, preterm birth-associated risk factors.

Our premise, based on studies showing that adverse outcomes following PTB are not inevitable,^3 7^ is that it is not PTB per se that has a deleterious effect on brain development, but rather, it is multiple, often interacting PTB-associated risk factors (PTB-RFs). These are biological, psychosocial and social/infrastructural and can affect parent or child, or be shared, for instance, maternal/infant stress, infection/inflammation, suboptimal infant nutrition, comorbidities of PTB and socioeconomic deprivation (figure 1).

### Rationale for study

To intervene against the harmful effects of PTB and support child development requires a quantitative understanding of PTB as a complex multidimensional risk exposure and new knowledge about how PTB-RFs modify brain development.

### The perinatal stress environment and outcomes after PTB

Prenatal exposure to maternal stress affects 10%–35% of children worldwide and is associated with adverse neuropsychiatric outcomes.^8^ Adaptation of the maternal hypothalamic–pituitary–adrenal (HPA) axis with consequent variation in the transfer of glucocorticoids to the developing fetus appears to be a key mechanism linking maternal stress to offspring neurodevelopment.^9^,^11^

Our recent studies suggest this could be an important axis for embedding PTB-RFs in brain development. First, maternal hair cortisol concentrations during pregnancy are associated with newborn amygdala architecture across the whole gestational age (GA) range, indicating that HPA axis activation links the prenatal stress environment to a key neural substrate of socioemotional development in childhood.^12^ Second, alterations in placental expression of genes regulating cortisol regeneration and placental transfer consistent with increased fetal glucocorticoid exposure occur in association with lower maternal socioeconomic status.^13^ Third, maternal consumption of glycyrrhizin (a potent inhibitor of placental 11β-hydroxysteroid dehydrogenase type 2, the ‘barrier’ to maternal glucocorticoids) is associated with adverse neurodevelopmental and neuropsychiatric outcomes in children.^14^ Fourth, extremely preterm infants (<28 weeks) tend to have blunted cortisol reactivity to vaccination at 4 months, suggesting low GA (or a coexposure such as repeated painful experiences during neonatal intensive care) programmes HPA axis adaptation. Fifth, neonatal hair glucocorticoids are a marker of both prenatal and postnatal physiological stressors in preterm infants.^15^ Finally, chronic HPA axis activation is a plausible mechanistic link between early life stress, altered brain morphology and major depression in adulthood.^16^ Based on these studies, we propose that atypical HPA axis activity is triggered by PTB-RFs and is an axis through which multidimensional exposures become embedded in the brain development of preterm infants.

### Systemic inflammation and EoP

Early studies revealed that neurodevelopmental outcomes are worse if infants are exposed to comorbidities of PTB characterised by systemic inflammation, for example, chorioamnionitis, bloodstream infection and necrotising enterocolitis.^17 18^ This is because inflammation alters oligodendrocyte precursor responses, increases proliferation and death and impairs maturation into myelin-forming oligodendrocytes.^19^ The consequent hypomyelination deprives axons of metabolic/trophic support and insulation for electrical impulse conduction, resulting in EoP.

Emerging evidence indicates that PTB is associated with sustained inflammation.^20 21^ Specific mediators of the adaptive and immune responses to PTB and its comorbidities have been linked to MRI features of EoP^22^; however, there are inconsistencies in the broader literature associating inflammation with neurodevelopment, in part because of the absence of standard peripheral biomarkers of low-level systemic, chronic inflammation in neonates and partly because study designs have relied on a single (or low frequency) measurement of selected proteins that are highly phasic, maturation-dependent and subject to swift and rapid concentration changes in plasma.

DNA methylation (DNAm) is an epigenetic mechanism that links environmental factors to regulation of gene expression. We propose that epigenetic scores, EpiScores, act as proxies for plasma protein levels and may provide a more accurate reflection of inflammatory exposure.^23 24^ EpiScores have been linked to major incident disease outcomes across the lifecourse^23 25 26^; they predict levels of inflammatory proteins and neuroinflammation-related outcomes, including brain structure and cognition in children and adults.^27^,^31^ and DNAm proxies have greater longitudinal stability and stronger associations with cognition than serum measures.^25 32^ These observations are of particular interest because age-related and birth weight-related differences in DNAm are present across a large number of CpGs.^33^,^35^ Recently, we have shown that PTB is associated with profound and widely distributed changes in the methylome (saliva) that are linked to MRI markers of white matter microstructure,^36^ and the EpiScore for C-reactive protein (DNAmCRP) captures the allostatic load of inflammatory burden in preterm infants and associates with EoP.^26^

### Aim

To identify the biological axes underlying abnormal brain development in preterm infants. We will characterise brain dysmaturation associated with PTB using neonatal MRI and use this to investigate the relationship between (1) HPA axis activity and (2) systemic inflammation indexed by DNAm and brain development.

#### Hypotheses

- Atypical activation of the HPA axis leads to EoP, indexed on MRI by dysmaturity (altered chronological brain age), reduced connectome complexity and markers of hypomyelination.
- DNAm proxies of systemic inflammation are present in preterm infants at term equivalent age and are associated with MRI features of EoP.
- The effect of PTB-RFs on brain development is mediated by alterations in the neonatal HPA axis and or chronic systemic inflammation.

## Methods and analysis

### Study design

This is an exposure-based cohort study between January 2023 and December 2027.

### Study setting

Participants are recruited from the women’s and children’s services of the Royal Infirmary of Edinburgh (RIE), NHS (National Health Service) Lothian. The RIE provides maternity and newborn services for residents of the City of Edinburgh and the Lothians. It receives 7000 deliveries annually and is the regional centre for all neonatal intensive care in South East Scotland. Approximately 100 infants with a birth weight of <1500 g receive intensive care at the RIE per annum.

### Study population

We plan to recruit 300 mother–infant dyads: 200 preterm deliveries with GA <32 weeks (exposed cases) and 100 term deliveries with GA >37 weeks (non-exposed comparators). GA is determined by the first trimester ultrasound scan. Preterm infants are included if a mother booked her pregnancy and delivered at the RIE (study centre) or if a mother booked her pregnancy at a hospital outside the study centre but was transferred to it with her baby in utero due to planned or expected birth <32 weeks.

Exclusion criteria: (1) Preterm infants who are transferred to the study centre postnatally for intensive care; (2) Infants with congenital anomalies: structural or functional anomalies (eg, metabolic disorders) that occur during intrauterine life and can be identified prenatally, at birth or later in life (WHO definition) and (3) Infants with a contraindication to MRI at 3Tesla determined by the Edinburgh Imaging safety policy, which is developed in accordance with UK Medicines and Healthcare Products Regulatory Agency safety guidelines.

### Participant selection and enrolment

Women who present to the RIE with threatened preterm labour and for whom delivery is planned or expected at less than 32 weeks GA. The comparator group (non-exposed term infants) are born to women who attend the RIE for antenatal care or delivery at >37 weeks GA. Potential participants are identified using NHS systems: maternity TRAK and the neonatal electronic patient record. As with prior work,^37^ this will result in a sampling distribution with fewer GA values between 32 and 37 weeks but will maximise sampling at important ends of the distribution within practical funding and recruitment constraints. Our analytical strategy, outlined below, will therefore benefit from a relative increase in power under consideration of important assumptions which apply for some but not all variables, including that a linear dose-response effect is present across the GA continuum between term and preterm.^38 39^

Consent to enter the study is sought from each participant after a full explanation has been given, an information leaflet offered and time allowed for consideration. Signed participant consent is obtained in two stages for the preterm group: first, for data collection from the antenatal period to the first week of postnatal life, and second, for data and samples over the rest of the neonatal period to the end of the study. Signed participant consent for all aspects of the study will be obtained in one stage for the comparator group. Consent to recontact for follow-on studies subject to additional funding is sought.

### Outcomes

Identification of targets in neuroendocrine stress and immune pathways that lead to atypical brain development in preterm infants indexed using 3 MRI markers of EoP: morphometric similarity networks (MSNs),^40^ hierarchical complexity (HC)^41^ and magnetisation transfer saturation imaging (MTsat).^42^ Table 1 summarises the assessment schedule, data collection methods, sample type/domain and the test or task. Data from cases and comparators are collected using the same data collection instruments.

**Table 1:** Schedule of assessments, data collection methods, sample type/domain and test/task

Data collection point	Age	Data collection method	Sample type/domain	Test/task	Notes
1	Antenatal	Administrative/electronic health records and interview	Medical, demographic, SES	Ethnic background and language spoken at home; parents’ education and employment; family income; family structure, housing, neighbourhood quality, parents’ mental health, social network and support	All participants
				History and exposures: life events, prescribed medications, alcohol, smoking, substances, pregnancy complications	
2	Birth	Administrative/electronic health records, questionnaire and tissue	Medical	Peripartum history and exposures, mother and infant	All participants
				Anthropometry	
			Placenta	Structured histopathology rating and storage. mRNA levels of glucocorticoid-related genes	Collect and store
			Umbilical cord blood	(1) 2 mL umbilical cord blood; (2) dried blood spot for storage	All participants(1) Endogenous glucocorticoids and metabolites (glucocorticoid release); glucocorticoid receptor in cord blood leucocytes (glucocorticoid signalling). (2) Inflammatory markers and DNA (collect and store)
			Hair, infant	Overall glucocorticoid secretion	All participants
			Hair, maternal	Overall glucocorticoid secretion	
			Saliva	Methylome	Term comparators
3	Neonatal	Tissue	Dried blood spot	Inflammatory markers and DNA	Collect and store at postnatal day 5, preterm subset.
		Tissue	Saliva	DNAm	Preterms at term equivalent age (38–44 weeks gestational age).
		Tissue	Hair, infant	Overall glucocorticoid secretion	Preterms at term equivalent age (38–44 weeks gestational age).
		Biosample	Faeces	Microbiome	Collect and store: stool between postnatal days 7–14 (cases and controls) and predischarge from neonatal intensive care and at 38–44 weeks (comparators).
		Administrative/electronic records and direct observation	Medical	Anthropometry	All participants
			Comorbidities and exposures	Comorbidities of preterm birth, medications, feed type and method; health status of control group.	
			Parent IQ	National Adult Reading Test, second edition*	
		MRI	Brain structure and connectivity	sMRI, dMRI.	MRI acquisition at 38–44 weeks. Morphometric similarity networks (chronological brain age), hierarchical complexity, magnetisation transfer imaging.
		Administrative/electronic records and questionnaire	Demographics and medical	Update perinatal history	All participants
				Edinburgh postnatal depression scale†	All participants
				Parenting daily hassles‡	
				WHO Quality of Life§	
				Adult temperament questionnaire- short (V.1.3)¶	

* Nelson HE, Wilson J^74^ (1991) (), NFER-Nelson, Windsor, UK.

† Cox *et al*., J.L., Holden, J.M., and Sagovsky,^75^ R. 1987. Detection of postnatal depression: Development of the 10-item Edinburgh Postnatal Depression Scale. British Journal of Psychiatry 150.

‡ Crnic K. A., Greenberg, M. T.^76^(1990). Minor parenting stresses with young children. (5),

§ WHOQOL-BREF version.

¶ Evans, D.E.,Rothbart, M.K.^77^(2007). Development of a model for adult temperament. Journal of Research in Personality, 41, .

DNAmDNA methylations-/d-MRI, structural/diffusion MRISES, socioeconomic status

### Questionnaire and records

Demographic and clinical information is extracted from the maternal and infant records. The tools to assess cognition, behaviour, well-being and family circumstances are listed in table 1.

### Neuroimaging

Participants are scanned using a Siemens MAGNETOM Prisma 3T MRI clinical scanner (Siemens Healthcare, Erlangen, Germany). For those at term-equivalent age, a 16-channel phased-array paediatric head receive coil is used to acquire sagittal three-dimensional (3D) T2-weighted (T2w) sampling perfection with application-optimised contrasts by using flip angle evolution (SPACE; 1 mm isotropic resolution, echo time (TE)=409 ms, repetition time (TR)=3200 ms), axial spin-echo echo-planar imaging multishell diffusion MRI (dMRI; 2 mm isotropic; 3×b=0 with reverse phase encoding, 16×b=0, 3×b=200, 6×b=500, 64×b=750, 64×b=2500 s/mm^2^ with optimal angular coverage^43^; TR/TE=3500/78 ms), sagittal 3D T1-weighted (T1w) magnetisation-prepared rapid acquisition with gradient echo (MPRAGE; 1 mm isotropic, TR/TE=1970/4.69 ms, inversion time (TI)=1100 ms, flip angle (FA)=9°) and *B*_1_^+^ field mapping (2.59×2.59×3.00 mm) scans. Magnetisation transfer (MT) saturation (MTSat) imaging is acquired, comprising three sagittal multiecho spoiled gradient echo scans (1.6 mm isotropic, TE=2.21, 6.31, 10.41 ms): (1) with gaussian MT preparation pulse (offset 1200 Hz, duration 9.984 ms, FA=500°; TR=75 ms, FA=5°); (2) proton-density weighted (PDw; TR=75 ms, FA=5°) and (3) T1w (TR=15 ms, FA=14°); an additional 8 echoes are acquired during the PDw scan to facilitate T_2_* and quantitative susceptibility mapping (TE=15.00, 20.00, 25.00, 30.00, 35.00, 40.00, 45.00, 50.00 ms).

If the infant stays settled, axial 3D susceptibility-weighted (0.75×0.75×3.0 mm, TR/TE=28/20 ms) and axial 2D fluid-attenuated inversion-recovery (FLAIR) BLADE (0.94×0.94×3.0 mm, TR/TE/TI=10 000/130/2606 ms) scans are acquired. Tissue heating and acoustic noise exposure are limited through active noise cancellation and by appropriately setting the gradient slew rate and other pulse sequence parameters. Participants are scanned in normal mode with respect to tissue heating and peripheral nerve stimulation. Further details of the protocol are provided in online supplemental file 1.

Conventional images are reported by a paediatric radiologist using a structured system.^44 45^ We use established methods to derive three markers of EoP: MSNs,^40^ HC^41^ and MTsat.^42^ Images are processed to derive features for secondary analyses, including but not limited to tract segmentations^46 47^ and structural regions of interest.^48^

### HPA axis activity (umbilical cord blood and maternal and neonatal hair)

Laboratory analyses of corticosteroids and their precursors and metabolites in plasma (2 mL) and hair (>0.3 cm 2 cm from neonates, up to 3 cm from mothers) are conducted at the University of Edinburgh Clinical Research Facility Mass Spectrometry Core. We have developed a robust method for steroid extraction from plasma (100 µL) and tissues,^49^ with quantification of cortisol and related corticosteroids, including cortisone, as well as dexamethasone and its metabolites, simultaneously by liquid chromatography tandem mass spectrometry, using a Sciex QTRAP 6500 (Warrington, UK) operated in positive ion electrospray ionisation with a Waters Acquity UPLC system (Manchester, UK).^50^

### DNAm (saliva)

DNA from saliva is extracted using prepIT.L2P reagent (DNA Genotek, Ontario, Canada). DNA will be bisulfite converted and methylation measured using Illumina HumanMethylationEPIC BeadChip (Illumina, San Diego, California, USA) at the Edinburgh Clinical Research Facility Genetics Core, Edinburgh, UK. The epigenetic measures of immune function, EpiScores, are calculated for each participant.^29^

### Dried blood spots (umbilical cord blood and neonatal dried blood spot)

Blood spots will be collected using Schleicher and Schuell 903 filter paper (6×3.2 mm spots per subject). Cards are stored at −20°C in the Centre for Reproductive Health and analysed in batch, subject to funding.^21 22 37^

### Placenta

Samples are stored at −80°C in the Edinburgh Reproductive Tissue BioBank for future analyses subject to approvals.

### Gut microbiome (faeces)

The gut microbiome plays a role in human health and disease, including child development,^51^ and is modified by age at birth, sex, mode of delivery, antibiotic exposure and feed type.^52^,^55^ The microbiome may mediate interactions of the preterm gut–brain axis.^56^,^58^ Three faecal samples are collected from cases during NICU care, and one from comparators within 2 weeks of birth. Maternal faecal samples will be collected. Samples will be processed and stored at −80°C for later analyses, subject to funding.

### Outcomes measurement

Samples and data will be collected at three time points in the perinatal period (table 1).

### Data analysis

Image processing is carried out at the University of Edinburgh using established pipelines for MSNs,^40^ HC^41^ and MTsat.^42^ There are two statistical approaches. In the first, multivariable regressions in a predictive framework are used to test whether PTB-RFs are associated with MSNs (brain age), HC (connectome architecture) and MTsat (a marker of myelination). For this, we will use standard statistical approaches (hypothesis testing, statistical association computations) and also machine learning methods ranging from feature selection to statistical mapping using widely used tools such as random forests and support vector machines.^59^,^61^ In the second, mediation analyses within a structural equation modelling framework are used to investigate the role of neuroendocrine stress activation and chronic inflammation as mediators of PTB-RFs on features of EoP.^29^ This simultaneously characterises associations among HPA axis activation/DNAm, PTB-RFs, and brain features and specifically tests the hypothesis that stress and/or chronic inflammation partly and significantly mediate associations between PTB-RFs and brain development.

### PTB-risk factor selection

PTB-RF selection is informed by the results of a national population-based cohort study that is a part of the PRENCOG programme. In summary, the weighted contributions of multidimensional PTB-RFs to neurodevelopmental outcomes and the real-world educational performance of children born preterm will be determined by linking the electronic health records of >100 000 infants born in England and held in the National Neonatal Research Database (NNRD) to the National Pupil Database (NPD). The NNRD is a Health Research Authority-approved National Information Asset that contains detailed, quality-assured data (Neonatal Data Set; NHS Information Standard DAPB1595) extracted from Electronic Patient Records.^62 63^ The NPD is a key Department for Education data store covering attainment for learners in England. Ethical and regulatory approvals for this record linkage and analysis are granted to CB^64^ (REC reference 21/EM/0130).

### Sample size

The sample size for groupwise comparisons of image data using biological variables is based on properties of the chosen EoP image phenotypes,^40 41^ and term and preterm differences we have observed in predictor variables (1) hair cortisol concentrations^15^: 401 pg/mg (262–615) vs 82 pg/mg (55–169), respectively and (2) group differences in DNAmCRP EpiScores.^26^

To maximise reproducibility, we will use (1) open-access neuroimaging protocols and standard operating procedures (SOPs) for sampling and analysis of biosamples, (2) behavioural assessment with clinical, dimensional and trait measures, multiple informants, direct observation and biometric data, (3) recommended reporting standards for neuroimaging, HPA axis activity and DNAm, (4) prespecified blind data processing, (5) analysis preregistration and (6) source code and data sharing. All manuscripts will be posted on preprint sites to facilitate another layer of peer review including critical insights into methodology.

### Patient and public involvement

**Figure 2:**
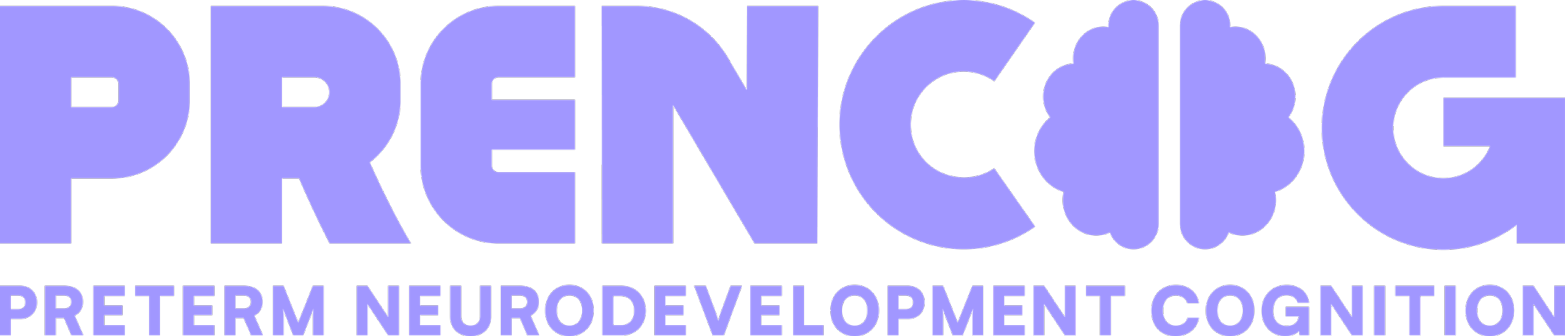
Codesigned PRENCOG logo, available in black and white.

The research questions were informed by parent priorities for research about childhood outcomes following PTB,^65^ attitudes of longitudinal cohort participants towards recent opportunities and controversies within health data science^66^ and stakeholders. The stakeholder groups are the Adult Preemie Advocacy Network (APAN, a network of adults who were born preterm, coauthor LI) and an eight-member parent advisory group. Stakeholders codesigned the research questions, reviewed the content of all participant-facing materials, including the participant information sheet and graphics (figures2  3), and informed our dissemination strategy. We commissioned a graphical design artist to create the PRENCOG study logo, a participant-facing infographic and a video animation to support recruitment (https://media.ed.ac.uk/media/Prencog_Neonatal/1_9llqdgsd).

**Figure 3:**
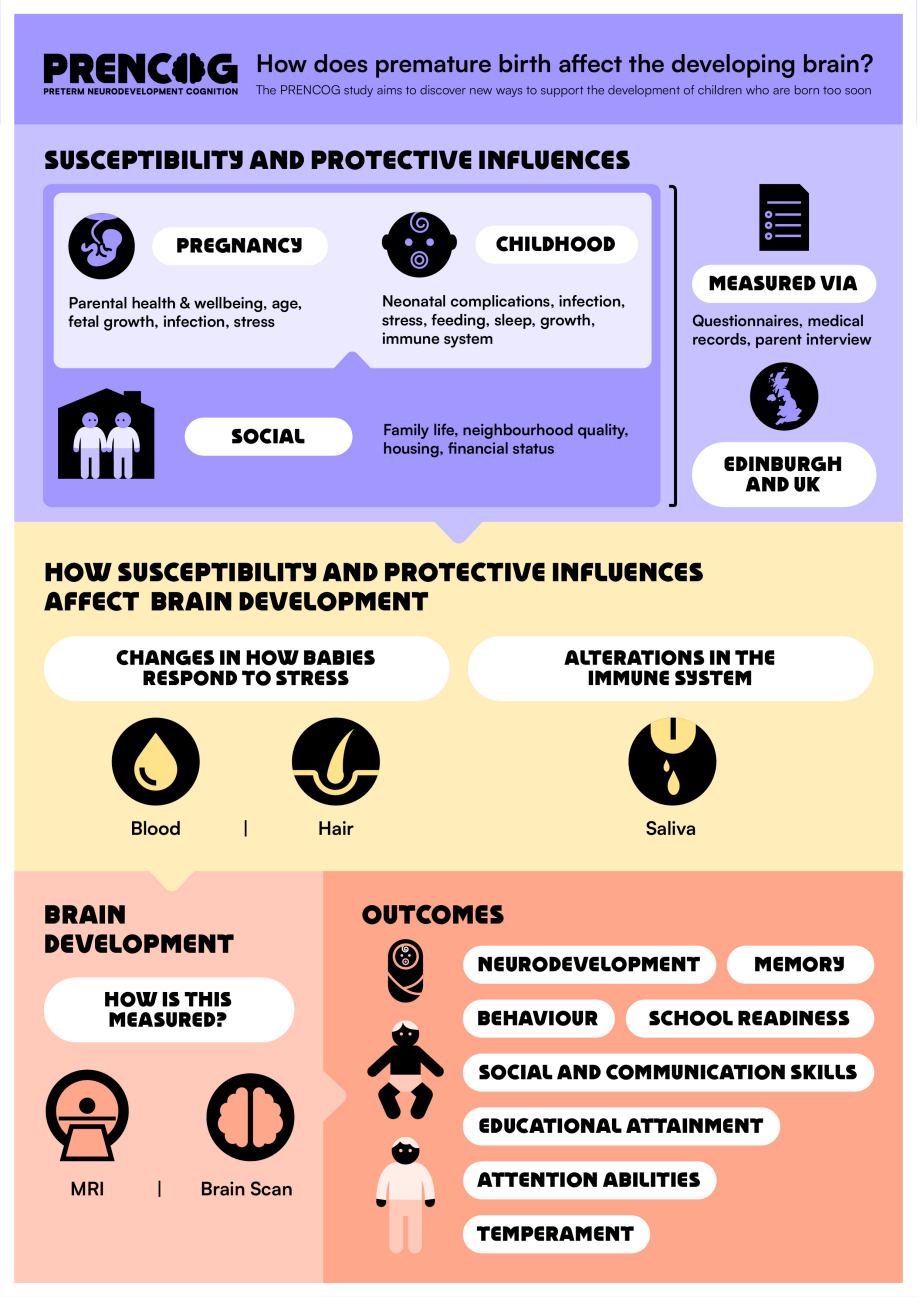
Codesigned participant-facing infographic.

### Related work

Children born preterm and term comparators enrolled in a separate longitudinal study (Theirworld Edinburgh Birth Cohort, TEBC) are invited for behavioural assessments at age five, as described in the TEBC protocol.^37^ With funding from the PRENCOG programme, MRI data are acquired at this time point. We have added the following behavioural tasks to those listed in the TEBC protocol: Theory of Mind booklet task,^67^ executive functions (Early Childhood Inhibitory Touchscreen Task,^68^ CORSI block tapping task^69^ and prohibited toy task),^70^ exploratory play (Novel toy task) and reading (from the Woodcock-Johnson IV subscales).

The goal is to define the functional and structural neural substrates of critical cognitive functions in preterm children and, using perinatal data, characterise factors that shape neurocognitive development at 5 years of age.

Five-year-old participants are scanned using a 32-channel phased-array adult head receive coil to acquire sagittal 3D T1w MPRAGE (1 mm isotropic, TR/TE/TI=2500/4.69/1180 ms, FA=7°), sagittal 3D T2w SPACE (0.9 mm isotropic, TR/TE=3200/407 ms), axial 2D T2w FLAIR (0.94×0.94×3.0 mm, TR/TE/TI=9500/124/2556 ms) and axial spin-echo echo-planar imaging multishell dMRI (2 mm isotropic; 3×b=0 with reverse phase encoding, 15×b=0, 3×b=200, 6×b=500, 64×b=1000, 64×b=2000 s/mm^2^ with optimal angular coverage^43^; TR/TE=2800/82 ms) scans. MTsat imaging is acquired, comprising three sagittal multiecho spoiled gradient echo scans (1.6 mm isotropic, TE=2.29, 6.33, 10.37 ms): (1) with MT preparation pulse as above (TR=35 ms, FA=5°), (2) PDw (TR=35 ms, FA=5°) and (3) T1w (TR=15 ms, FA=18°). *B*_0_ field mapping (2.3 mm isotropic) is acquired prior to three functional MRI scans, which are acquired using 2D gradient echo echo-planar imaging (2.3 mm isotropic, TR/TE=1000/30 ms, FA=60°). During the functional MRI scans, children view selected movies that are age appropriate, engaging and enable characterising neural correlates of several cognitive functions. Further details of the protocol are provided in online supplemental file 2.

We use an information booklet, an animation (https://media.ed.ac.uk/media/PRENCOG_5YEAROLDAPPOINTMENT_ANIMATION/1_akzmmsc4) and a mock scan to acclimate 5-year-old participants to the MRI environment and to train them to stay very still (ie, <2 mm motion).^71^ The children use in-ear headphones to listen to the soundtrack of movies and for communication with the researchers operating the scan in the control room. The researchers communicate with children approximately every 5 min during the scan; children respond by speaking aloud. The in-ear headphones reduce the MRI noise to safe levels; soft pads offer additional hearing protection and help to stabilise children’s heads. An additional member of the research team stands near the child’s feet and the bore of the scanner to monitor the child during the scan. If the child moves, this researcher pats the child’s leg as a reminder to stay still.

We will use functional, structural and diffusion MRI data to investigate differences in brain structure and function as a function of GA, in 5-year-old children. Our analyses will include focused studies of responses in specific functional networks that underly particular cognitive domains (eg, social cognition, attention, language, reading), as well as whole-brain studies characterising distributed impacts of PTB (eg, on white matter tract integrity, MTsat, network architecture and cortical morphology). These data also enable longitudinal studies of brain structure, given that participants completed structural and diffusion MRI scans as neonates. This line of work will build directly on evidence from the neonatal scans by testing for sustained impacts of GA on brain development at age 5 years.^40^,^42^ As described above, we will also investigate the relative roles of other risk and protective factors (eg, SES,^48^ maternal stress,^12^ infant nutrition^72^ and early linguistic environment^73^) on neurocognitive development in children born preterm.

### Ethics and dissemination

Ethical favourable opinion for all neonatal studies has been obtained from the South East Scotland Research Ethics Committee 02 (23/SS/0067) and NHS Lothian Research and Development (2023/0150). A favourable ethical opinion for data collection and analyses in related work on 5 years has been provided by the South East Scotland Research Ethics Committee 01 (16/SS/0154).

Statistical analysis plans for the main analyses will be published on Open Science Framework.

Results will be reported to the UKRI Medical Research Council. They will be presented at national and international scientific conferences and summarised on a study-specific website in lay form and via a newsletter for families (https://www.ed.ac.uk/centre-reproductive-health/prencog). They will be published on preprint servers and in peer-reviewed publications. At the end of the programme of work, we will cocreate with stakeholders a scientific animation to illustrate the research insights and offer accessible and digestible information to families. Stakeholders (APAN) will disseminate the main findings via their social media channels and website. We will engage with the University of Edinburgh public relations and media office to ensure maximum publicity and benefit.

### Safety considerations

We do not anticipate risk from any of the biosample collections or questionnaires.

The MRI scanner generates loud acoustic noise, so flexible earplugs and earmuffs are used to prevent noise discomfort and to encourage infants to sleep. We use established procedures described ensuring infant safety and physiological stability during imaging.^37^ The infant has continuous monitoring of vital signs (heart rate and oxygen saturation) with an MR conditional patient monitor. The attending clinical practitioner will record observations every 5 min until 1 hour after the infant has woken up, and the scan will be stopped if there are any abnormalities in monitoring. Full neonatal resuscitation facilities are available on site. SOPs for ensuring safety in the MRI environment are in place at the Edinburgh Imaging facility.

## supplementary material

10.1136/bmjopen-2024-085365 online supplemental file 1

10.1136/bmjopen-2024-085365 online supplemental file 2
