# Supplementary material for: Preterm birth as a determinant of neurodevelopment and cognition in children (PRENCOG): protocol for an exposure-based cohort study in the UK
Source: BMJ Open. 2024 Sep 16;14(9):e085365. doi: 10.1136/bmjopen-2024-085365 (PMC11409314; doi:10.1136/bmjopen-2024-085365)
Supplement: online supplemental file 1 [file bmjopen-14-9-s001.pdf]

**Table of contents**

\\Protocol Development

MJT

Neonate-development

PRENCOG_neonate

localizer_neonate
t2_haste_localiser
t2_blade_v3
t2_space_sag_p4_iso_v2x
DTI_Neonate_v6b_dummy
DTI_Neonate_v6b_rev
DTI_Neonate_v6b_pt1
DTI_Neonate_v6b_pt2
MPRAGE-v4
B1Map_tra
MTSatOn_neonate_v3
MTSatOff_neonate_v3
MTSatT1_neonate_v3
SWI_v2
t2_blade_dark-fluid_tra_v3

| \\Protocol Development\MJT\Neonate-development\PRENCOG_neonate\localizer_neonate |
|---|
| TA: 0:12 PM: REF Voxel size: 0.5×0.5×7.0 mmPAT: Off Rel. SNR: 1.00 : fl |

## Properties

| | |
|---|---|
| Prio recon | On |
| Load images to viewer | On |
| Inline movie | Off |
| Auto store images | On |
| Load images to stamp segments | On |
| Load images to graphic segments | On |
| Auto open inline display | Off |
| Auto close inline display | Off |
| Start measurement without further preparation | Off |
| Wait for user to start | Off |
| Start measurements | Single measurement |

## Routine

| | |
|---|---|
| Slice group | 1 |
| Slices | 1 |
| Dist. factor | 20 % |
| Position | Isocenter |
| Orientation | Sagittal |
| Phase enc. dir. | A >> P |
| Slice group | 2 |
| Slices | 1 |
| Dist. factor | 20 % |
| Position | L0.0 P47.8 F62.3 mm |
| Orientation | Transversal |
| Phase enc. dir. | A >> P |
| Slice group | 3 |
| Slices | 1 |
| Dist. factor | 20 % |
| Position | L0.0 P47.8 F62.3 mm |
| Orientation | Coronal |
| Phase enc. dir. | R >> L |
| AutoAlign | --- |
| Phase oversampling | 0 % |
| FoV read | 250 mm |
| FoV phase | 100.0 % |
| Slice thickness | 7.0 mm |
| TR | 7.5 ms |
| TE | 3.69 ms |
| Averages | 2 |
| Concatenations | 3 |
| Filter | Prescan Normalize, Elliptical filter |
| Coil elements | PeH;PeN |

## Contrast - Common

| | |
|---|---|
| TR | 7.5 ms |
| TE | 3.69 ms |
| TD | 0 ms |
| MTC | Off |
| Magn. preparation | None |
| Flip angle | 20 deg |
| Fat suppr. | None |
| Water suppr. | None |
| SWI | Off |

## Contrast - Dynamic

| | |
|---|---|
| Averages | 2 |
| Averaging mode | Short term |
| Reconstruction | Magnitude |
| Measurements | 1 |

## Contrast - Dynamic

| | |
|---|---|
| Multiple series | Each measurement |

## Resolution - Common

| | |
|---|---|
| FoV read | 250 mm |
| FoV phase | 100.0 % |
| Slice thickness | 7.0 mm |
| Base resolution | 256 |
| Phase resolution | 91 % |
| Phase partial Fourier | Off |
| Interpolation | On |

## Resolution - iPAT

| | |
|---|---|
| PAT mode | None |

## Resolution - Filter Image

| | |
|---|---|
| Image Filter | Off |
| Distortion Corr. | Off |
| Prescan Normalize | On |
| Unfiltered images | Off |
| Normalize | Off |
| B1 filter | Off |

## Resolution - Filter Rawdata

| | |
|---|---|
| Raw filter | Off |
| Elliptical filter | On |

## Geometry - Common

| | |
|---|---|
| Slice group | 1 |
| Slices | 1 |
| Dist. factor | 20 % |
| Position | Isocenter |
| Orientation | Sagittal |
| Phase enc. dir. | A >> P |
| Slice group | 2 |
| Slices | 1 |
| Dist. factor | 20 % |
| Position | L0.0 P47.8 F62.3 mm |
| Orientation | Transversal |
| Phase enc. dir. | A >> P |
| Slice group | 3 |
| Slices | 1 |
| Dist. factor | 20 % |
| Position | L0.0 P47.8 F62.3 mm |
| Orientation | Coronal |
| Phase enc. dir. | R >> L |
| FoV read | 250 mm |
| FoV phase | 100.0 % |
| Slice thickness | 7.0 mm |
| TR | 7.5 ms |
| Multi-slice mode | Sequential |
| Series | Interleaved |
| Concatenations | 3 |

## Geometry - AutoAlign

| | |
|---|---|
| Slice group | 1 |
| Position | Isocenter |
| Orientation | Sagittal |
| Phase enc. dir. | A >> P |
| Slice group | 2 |
| Position | L0.0 P47.8 F62.3 mm |

## Geometry - AutoAlign

| | |
|---|---|
| Orientation | Transversal |
| Phase enc. dir. | A >> P |
| Slice group | 3 |
| Position | L0.0 P47.8 F62.3 mm |
| Orientation | Coronal |
| Phase enc. dir. | R >> L |
| AutoAlign | --- |
| Initial Position | Isocenter |
| L | 0.0 mm |
| P | 0.0 mm |
| H | 0.0 mm |
| Initial Rotation | 0.00 deg |
| Initial Orientation | Sagittal |

## Geometry - Saturation

| | |
|---|---|
| Saturation mode | Standard |
| Fat suppr. | None |
| Water suppr. | None |
| Special sat. | None |

## Geometry - Tim Planning Suite

| | |
|---|---|
| Set-n-Go Protocol | Off |
| Table position | H |
| Table position | 0 mm |
| Inline Composing | Off |

## System - Miscellaneous

| | |
|---|---|
| Positioning mode | REF |
| Table position | H |
| Table position | 0 mm |
| MSMA | S - C - T |
| Sagittal | R >> L |
| Coronal | A >> P |
| Transversal | F >> H |
| Coil Combine Mode | Adaptive Combine |
| Save uncombined | Off |
| Matrix Optimization | Off |
| AutoAlign | --- |
| Coil Select Mode | Default |

## System - Adjustments

| | |
|---|---|
| B0 Shim mode | Tune up |
| B1 Shim mode | TrueForm |
| Adjust with body coil | Off |
| Confirm freq. adjustment | Off |
| Assume Dominant Fat | Off |
| Assume Silicone | Off |
| Adjustment Tolerance | Auto |

## System - Adjust Volume

| | |
|---|---|
| Position | Isocenter |
| Orientation | Transversal |
| Rotation | 0.00 deg |
| A >> P | 263 mm |
| R >> L | 350 mm |
| F >> H | 350 mm |
| Reset | Off |

## System - pTx Volumes

| | |
|---|---|
| B1 Shim mode | TrueForm |
| Excitation | Slice-sel. |

## System - Tx/Rx

| | |
|---|---|
| Frequency 1H | 123.244475 MHz |
| Correction factor | 1 |
| Gain | High |
| Img. Scale Cor. | 1.000 |
| Reset | Off |
| ? Ref. amplitude 1H | 0.000 V |

## Physio - Signal1

| | |
|---|---|
| 1st Signal/Mode | None |
| TR | 7.5 ms |
| Concatenations | 3 |
| Segments | 1 |

## Physio - Cardiac

| | |
|---|---|
| Tagging | None |
| Magn. preparation | None |
| Fat suppr. | None |
| Dark blood | Off |
| FoV read | 250 mm |
| FoV phase | 100.0 % |
| Phase resolution | 91 % |

## Physio - PACE

| | |
|---|---|
| Resp. control | Off |
| Concatenations | 3 |

## Inline - Common

| | |
|---|---|
| Subtract | Off |
| Measurements | 1 |
| StdDev | Off |
| Liver registration | Off |
| Save original images | On |

## Inline - MIP

| | |
|---|---|
| MIP-Sag | Off |
| MIP-Cor | Off |
| MIP-Tra | Off |
| MIP-Time | Off |
| Save original images | On |

## Inline - Soft Tissue

| | |
|---|---|
| Wash - In | Off |
| Wash - Out | Off |
| TTP | Off |
| PEI | Off |
| MIP - time | Off |
| Measurements | 1 |

## Inline - Composing

| | |
|---|---|
| Inline Composing | Off |
| Distortion Corr. | Off |

## Inline - MapIt

| | |
|---|---|
| Save original images | On |
| MapIt | None |
| Flip angle | 20 deg |
| Measurements | 1 |
| Contrasts | 1 |
| TR | 7.5 ms |
| TE | 3.69 ms |

## Sequence - Part 1

| | |
|---|---|
| Introduction | On |

## Sequence - Part 1

| | |
|---|---|
| Dimension | 2D |
| Phase stabilisation | Off |
| Asymmetric echo | Allowed |
| Contrasts | 1 |
| Flow comp. | No |
| Multi-slice mode | Sequential |
| Bandwidth | 320 Hz/Px |

## Sequence - Part 2

| | |
|---|---|
| Segments | 1 |
| Acoustic noise reduction | None |
| RF pulse type | Fast |
| Gradient mode | Fast |
| Excitation | Slice-sel. |
| RF spoiling | On |

## Sequence - Assistant

| | |
|---|---|
| Mode | Off |
| Allowed delay | 0 s |

**\\Protocol Development\MJT\Neonate-development\PRENCOG_neonate\t2_haste_localiser**

TA: 6.0 s PM: REF Voxel size: 0.7×0.7×4.0 mmPAT: 2 Rel. SNR: 1.00 : h

## Properties

| | |
|---|---|
| Prio recon | Off |
| Load images to viewer | On |
| Inline movie | Off |
| Auto store images | On |
| Load images to stamp segments | On |
| Load images to graphic segments | On |
| Auto open inline display | Off |
| Auto close inline display | Off |
| Start measurement without further preparation | Off |
| Wait for user to start | Off |
| Start measurements | Single measurement |

## Routine

| | |
|---|---|
| Slice group | 1 |
| Slices | 1 |
| Dist. factor | 30 % |
| Position | Isocenter |
| Orientation | Sagittal |
| Phase enc. dir. | A >> P |
| Slice group | 2 |
| Slices | 1 |
| Dist. factor | 30 % |
| Position | L0.0 P0.0 H5.2 mm |
| Orientation | Transversal |
| Phase enc. dir. | R >> L |
| Slice group | 3 |
| Slices | 1 |
| Dist. factor | 30 % |
| Position | L0.0 P0.0 H10.4 mm |
| Orientation | Coronal |
| Phase enc. dir. | R >> L |
| AutoAlign | --- |
| Phase oversampling | 0 % |
| FoV read | 220 mm |
| FoV phase | 100.0 % |
| Slice thickness | 4.0 mm |
| TR | 1500.0 ms |
| TE | 94 ms |
| Averages | 1 |
| Concatenations | 1 |
| Filter | Prescan Normalize, Elliptical filter |
| Coil elements | HE1-4 |

## Contrast - Common

| | |
|---|---|
| TR | 1500.0 ms |
| TE | 94 ms |
| MTC | Off |
| Magn. preparation | None |
| Flip angle | 150 deg |
| Fat suppr. | None |
| Water suppr. | None |
| Restore magn. | Off |

## Contrast - Dynamic

| | |
|---|---|
| Averages | 1 |
| Averaging mode | Long term |
| Reconstruction | Magnitude |
| Measurements | 1 |
| Multiple series | Each measurement |

## Resolution - Common

| | |
|---|---|
| FoV read | 220 mm |
| FoV phase | 100.0 % |
| Slice thickness | 4.0 mm |
| Base resolution | 320 |
| Phase resolution | 80 % |
| Phase partial Fourier | 4/8 |
| Interpolation | Off |

## Resolution - iPAT

| | |
|---|---|
| PAT mode | GRAPPA |
| Accel. factor PE | 2 |
| Ref. lines PE | 24 |
| Reference scan mode | Integrated |

## Resolution - Filter Image

| | |
|---|---|
| Image Filter | Off |
| Distortion Corr. | Off |
| Prescan Normalize | On |
| Unfiltered images | Off |
| Normalize | Off |
| B1 filter | Off |

## Resolution - Filter Rawdata

| | |
|---|---|
| Raw filter | Off |
| Elliptical filter | On |

## Geometry - Common

| | |
|---|---|
| Slice group | 1 |
| Slices | 1 |
| Dist. factor | 30 % |
| Position | Isocenter |
| Orientation | Sagittal |
| Phase enc. dir. | A >> P |
| Slice group | 2 |
| Slices | 1 |
| Dist. factor | 30 % |
| Position | L0.0 P0.0 H5.2 mm |
| Orientation | Transversal |
| Phase enc. dir. | R >> L |
| Slice group | 3 |
| Slices | 1 |
| Dist. factor | 30 % |
| Position | L0.0 P0.0 H10.4 mm |
| Orientation | Coronal |
| Phase enc. dir. | R >> L |
| FoV read | 220 mm |
| FoV phase | 100.0 % |
| Slice thickness | 4.0 mm |
| TR | 1500.0 ms |
| Multi-slice mode | Single shot |
| Series | Interleaved |
| Concatenations | 1 |

## Geometry - AutoAlign

| | |
|---|---|
| Slice group | 1 |
| Position | Isocenter |
| Orientation | Sagittal |
| Phase enc. dir. | A >> P |
| Slice group | 2 |
| Position | L0.0 P0.0 H5.2 mm |

## Geometry - AutoAlign

| | |
|---|---|
| Orientation | Transversal |
| Phase enc. dir. | R >> L |
| Slice group | 3 |
| Position | L0.0 P0.0 H10.4 mm |
| Orientation | Coronal |
| Phase enc. dir. | R >> L |
| AutoAlign | --- |
| Initial Position | Isocenter |
| L | 0.0 mm |
| P | 0.0 mm |
| H | 0.0 mm |
| Initial Rotation | 0.00 deg |
| Initial Orientation | Sagittal |

## Geometry - Saturation

| | |
|---|---|
| Fat suppr. | None |
| Water suppr. | None |
| Restore magn. | Off |
| Special sat. | None |

## Geometry - Navigator

## Geometry - Tim Planning Suite

| | |
|---|---|
| Set-n-Go Protocol | Off |
| Table position | H |
| Table position | 0 mm |
| Inline Composing | Off |

## System - Miscellaneous

| | |
|---|---|
| Positioning mode | REF |
| Table position | H |
| Table position | 0 mm |
| MSMA | S - C - T |
| Sagittal | R >> L |
| Coronal | A >> P |
| Transversal | F >> H |
| Coil Combine Mode | Adaptive Combine |
| Save uncombined | Off |
| Matrix Optimization | Off |
| AutoAlign | --- |
| Coil Select Mode | On - AutoCoilSelect |

## System - Adjustments

| | |
|---|---|
| B0 Shim mode | Tune up |
| B1 Shim mode | TrueForm |
| Adjust with body coil | Off |
| Confirm freq. adjustment | Off |
| Assume Dominant Fat | Off |
| Assume Silicone | Off |
| Adjustment Tolerance | Auto |

## System - Adjust Volume

| | |
|---|---|
| Position | Isocenter |
| Orientation | Transversal |
| Rotation | 0.00 deg |
| A >> P | 263 mm |
| R >> L | 350 mm |
| F >> H | 350 mm |
| Reset | Off |

## System - pTx Volumes

| | |
|---|---|
| B1 Shim mode | TrueForm |

## System - Tx/Rx

| | |
|---|---|
| Frequency 1H | 123.244475 MHz |
| Correction factor | 1 |
| Gain | High |
| Img. Scale Cor. | 1.000 |
| Reset | Off |
| ? Ref. amplitude 1H | 0.000 V |

## Physio - Signal1

| | |
|---|---|
| 1st Signal/Mode | None |
| TR | 1500.0 ms |
| Concatenations | 1 |

## Physio - Cardiac

| | |
|---|---|
| Magn. preparation | None |
| Fat suppr. | None |
| Dark blood | Off |
| FoV read | 220 mm |
| FoV phase | 100.0 % |
| Phase resolution | 80 % |

## Physio - PACE

| | |
|---|---|
| Resp. control | Off |
| Concatenations | 1 |

## Inline - Common

| | |
|---|---|
| Subtract | Off |
| Measurements | 1 |
| StdDev | Off |
| Save original images | On |

## Inline - MIP

| | |
|---|---|
| MIP-Sag | Off |
| MIP-Cor | Off |
| MIP-Tra | Off |
| MIP-Time | Off |
| Save original images | On |

## Inline - Composing

| | |
|---|---|
| Inline Composing | Off |
| Distortion Corr. | Off |

## Sequence - Part 1

| | |
|---|---|
| Introduction | On |
| Dimension | 2D |
| Contrasts | 1 |
| Flow comp. | No |
| Multi-slice mode | Single shot |
| Echo spacing | 7.22 ms |
| Bandwidth | 601 Hz/Px |

## Sequence - Part 2

| | |
|---|---|
| RF pulse type | Normal |
| Gradient mode | Whisper |
| Hyperecho | Off |
| Turbo factor | 256 |

## Sequence - Assistant

| | |
|---|---|
| Mode | Min flip angle |
| Min flip angle | 130 deg |
| Allowed delay | 60 s |

| \\Protocol Development\MJT\Neonate-development\PRENCOG_neonate\t2_blade_v3 |
|---|
| TA: 2:29 PM: REF Voxel size: 0.7×0.7×3.0 mmPAT: 2 Rel. SNR: 1.00 : qtseBR_rr |

## Properties

| | |
|---|---|
| Prio recon | Off |
| Load images to viewer | On |
| Inline movie | Off |
| Auto store images | On |
| Load images to stamp segments | On |
| Load images to graphic segments | Off |
| Auto open inline display | Off |
| Auto close inline display | Off |
| Start measurement without further preparation | Off |
| Wait for user to start | Off |
| Start measurements | Single measurement |

## Routine

| | |
|---|---|
| Slice group | 1 |
| Slices | 40 |
| Dist. factor | 0 % |
| Position | R1.2 P40.0 H50.2 mm |
| Orientation | Transversal |
| Phase enc. dir. | A >> P |
| AutoAlign | --- |
| Phase oversampling | 0.0 % |
| FoV read | 220 mm |
| FoV phase | 100.0 % |
| Slice thickness | 3.0 mm |
| TR | 4100.0 ms |
| TE | 207 ms |
| Averages | 1 |
| Concatenations | 4 |
| Filter | Prescan Normalize |
| Coil elements | PeH;PeN |

## Contrast - Common

| | |
|---|---|
| TR | 4100.0 ms |
| TE | 207 ms |
| TD | 0.0 ms |
| MTC | Off |
| Magn. preparation | None |
| Flip angle | 90 deg |
| Fat suppr. | None |
| Water suppr. | None |
| Restore magn. | On |

## Contrast - Dynamic

| | |
|---|---|
| Averages | 1 |
| Averaging mode | Short term |
| Reconstruction | Magnitude |
| Measurements | 1 |
| Multiple series | Each measurement |

## Resolution - Common

| | |
|---|---|
| FoV read | 220 mm |
| FoV phase | 100.0 % |
| Slice thickness | 3.0 mm |
| Base resolution | 320 |
| BLADE coverage | 100.0 % |
| Trajectory | BLADE |
| Interpolation | Off |

## Resolution - iPAT

| | |
|---|---|
| PAT mode | GRAPPA |
| Accel. factor PE | 2 |
| Ref. lines PE | 8 |
| Reference scan mode | Integrated |

## Resolution - Filter Image

| | |
|---|---|
| Image Filter | Off |
| Distortion Corr. | Off |
| Prescan Normalize | On |
| Unfiltered images | Off |
| Normalize | Off |
| B1 filter | Off |

## Resolution - Filter Rawdata

| | |
|---|---|
| Raw filter | Off |
| Elliptical filter | Off |

## Geometry - Common

| | |
|---|---|
| Slice group | 1 |
| Slices | 40 |
| Dist. factor | 0 % |
| Position | R1.2 P40.0 H50.2 mm |
| Orientation | Transversal |
| Phase enc. dir. | A >> P |
| FoV read | 220 mm |
| FoV phase | 100.0 % |
| Slice thickness | 3.0 mm |
| TR | 4100.0 ms |
| Multi-slice mode | Interleaved |
| Series | Interleaved |
| Concatenations | 4 |

## Geometry - AutoAlign

| | |
|---|---|
| Slice group | 1 |
| Position | R1.2 P40.0 H50.2 mm |
| Orientation | Transversal |
| Phase enc. dir. | A >> P |
| AutoAlign | --- |
| Initial Position | R1.2 P40.0 H50.2 |
| R | 1.2 mm |
| P | 40.0 mm |
| H | 50.2 mm |
| Initial Rotation | 0.00 deg |
| Initial Orientation | Transversal |

## Geometry - Saturation

| | |
|---|---|
| Fat suppr. | None |
| Water suppr. | None |
| Restore magn. | On |
| Special sat. | None |

## Geometry - Navigator

## Geometry - Tim Planning Suite

| | |
|---|---|
| Set-n-Go Protocol | Off |
| Table position | H |
| Table position | 0 mm |
| Inline Composing | Off |

## System - Miscellaneous

| | |
|---|---|
| Positioning mode | REF |
| Table position | H |
| Table position | 0 mm |
| MSMA | S - C - T |
| Sagittal | R >> L |
| Coronal | A >> P |
| Transversal | F >> H |
| Coil Combine Mode | Adaptive Combine |
| Save uncombined | Off |
| Matrix Optimization | Off |
| AutoAlign | --- |
| Coil Select Mode | On - AutoCoilSelect |

## System - Adjustments

| | |
|---|---|
| B0 Shim mode | Tune up |
| B1 Shim mode | TrueForm |
| Adjust with body coil | Off |
| Confirm freq. adjustment | Off |
| Assume Dominant Fat | Off |
| Assume Silicone | Off |
| Adjustment Tolerance | Auto |

## System - Adjust Volume

| | |
|---|---|
| Position | Isocenter |
| Orientation | Transversal |
| Rotation | 0.00 deg |
| A >> P | 263 mm |
| R >> L | 350 mm |
| F >> H | 350 mm |
| Reset | Off |

## System - pTx Volumes

| | |
|---|---|
| B1 Shim mode | TrueForm |

## System - Tx/Rx

| | |
|---|---|
| Frequency 1H | 123.244475 MHz |
| Correction factor | 1 |
| Gain | High |
| Img. Scale Cor. | 1.000 |
| Reset | Off |
| ? Ref. amplitude 1H | 0.000 V |

## Physio - Signal1

| | |
|---|---|
| 1st Signal/Mode | None |
| TR | 4100.0 ms |
| Concatenations | 4 |

## Physio - Cardiac

| | |
|---|---|
| Magn. preparation | None |
| Fat suppr. | None |
| Dark blood | Off |
| FoV read | 220 mm |
| FoV phase | 100.0 % |
| BLADE coverage | 100.0 % |
| Trajectory | BLADE |

## Physio - PACE

| | |
|---|---|
| Resp. control | Off |
| Concatenations | 4 |

## Inline - Common

| | |
|---|---|
| Subtract | Off |
| Measurements | 1 |
| StdDev | Off |
| Save original images | On |

## Inline - MIP

| | |
|---|---|
| MIP-Sag | Off |
| MIP-Cor | Off |
| MIP-Tra | Off |
| MIP-Time | Off |
| Save original images | On |

## Inline - Composing

| | |
|---|---|
| Inline Composing | Off |
| Distortion Corr. | Off |

## Sequence - Part 1

| | |
|---|---|
| Introduction | On |
| Dimension | 2D |
| Compensate T2 decay | Off |
| Contrasts | 1 |
| Flow comp. | Read |
| Multi-slice mode | Interleaved |
| Free echo spacing | Off |
| Echo spacing | 10.9 ms |
| Bandwidth | 363 Hz/Px |

## Sequence - Part 2

| | |
|---|---|
| Define | Turbo factor |
| Echo trains per slice | 8 |
| Phase correction | Automatic |
| Acoustic noise reduction | Active |
| RF pulse type | Low SAR |
| Gradient mode | Fast |
| Hyperecho | On |
| WARP | Off |
| Motion correction | On |
| Red. EC sensitivity | Off |
| Turbo factor | 36 |

## Sequence - Assistant

| | |
|---|---|
| Mode | Off |
| Allowed delay | 30 s |

**\\Protocol Development\MJT\Neonate-development\PRENCOG_neonate\t2_space_sag_p4_iso_v2x**

TA: 2:13 PM: REF Voxel size: 1.0×1.0×1.0 mmPAT: 4 Rel. SNR: 1.00 : spcR

## Properties

| | |
|---|---|
| Prio recon | Off |
| Load images to viewer | On |
| Inline movie | Off |
| Auto store images | On |
| Load images to stamp segments | Off |
| Load images to graphic segments | Off |
| Auto open inline display | Off |
| Auto close inline display | Off |
| Start measurement without further preparation | Off |
| Wait for user to start | Off |
| Start measurements | Single measurement |

## Routine

| | |
|---|---|
| Slab group | 1 |
| Slabs | 1 |
| Position | R1.2 P36.9 H0.0 mm |
| Orientation | Sagittal |
| Phase enc. dir. | A >> P |
| AutoAlign | --- |
| Phase oversampling | 0 % |
| Slice oversampling | 0.0 % |
| Slices per slab | 160 |
| FoV read | 128 mm |
| FoV phase | 150.0 % |
| Slice thickness | 1.00 mm |
| TR | 3200 ms |
| TE | 409 ms |
| Averages | 1.4 |
| Concatenations | 1 |
| Filter | Raw filter, Prescan Normalize |
| Coil elements | PeH;PeN |

## Contrast - Common

| | |
|---|---|
| TR | 3200 ms |
| TE | 409 ms |
| MTC | Off |
| Magn. preparation | None |
| Fat suppr. | Fat sat. |
| Fat sat. mode | Strong |
| Blood suppr. | Off |
| Restore magn. | On |

## Contrast - Dynamic

| | |
|---|---|
| Averages | 1.4 |
| Reconstruction | Magnitude |
| Measurements | 1 |
| Multiple series | Each measurement |

## Resolution - Common

| | |
|---|---|
| FoV read | 128 mm |
| FoV phase | 150.0 % |
| Slice thickness | 1.00 mm |
| Base resolution | 128 |
| Phase resolution | 100 % |
| Slice resolution | 100 % |
| Phase partial Fourier | Allowed |
| Slice partial Fourier | Off |
| Interpolation | Off |

## Resolution - iPAT

| | |
|---|---|
| PAT mode | GRAPPA |
| Accel. factor PE | 2 |
| Ref. lines PE | 24 |
| Accel. factor 3D | 2 |
| Ref. lines 3D | 24 |
| Reference scan mode | Integrated |

## Resolution - Filter Image

| | |
|---|---|
| Image Filter | Off |
| Distortion Corr. | Off |
| Prescan Normalize | On |
| Unfiltered images | Off |
| Normalize | Off |
| B1 filter | Off |

## Resolution - Filter Rawdata

| | |
|---|---|
| Raw filter | On |
| Elliptical filter | Off |

## Geometry - Common

| | |
|---|---|
| Slab group | 1 |
| Slabs | 1 |
| Position | R1.2 P36.9 H0.0 mm |
| Orientation | Sagittal |
| Phase enc. dir. | A >> P |
| Slice oversampling | 0.0 % |
| Slices per slab | 160 |
| FoV read | 128 mm |
| FoV phase | 150.0 % |
| Slice thickness | 1.00 mm |
| TR | 3200 ms |
| Series | Interleaved |
| Concatenations | 1 |

## Geometry - AutoAlign

| | |
|---|---|
| Slab group | 1 |
| Position | R1.2 P36.9 H0.0 mm |
| Orientation | Sagittal |
| Phase enc. dir. | A >> P |
| AutoAlign | --- |
| Initial Position | R1.2 P36.9 H0.0 |
| R | 1.2 mm |
| P | 36.9 mm |
| H | 0.0 mm |
| Initial Rotation | 0.00 deg |
| Initial Orientation | Sagittal |

## Geometry - Saturation

| | |
|---|---|
| Fat suppr. | Fat sat. |
| Fat sat. mode | Strong |
| Restore magn. | On |
| Special sat. | None |

## Geometry - Navigator

## Geometry - Tim Planning Suite

| | |
|---|---|
| Set-n-Go Protocol | Off |
| Table position | H |
| Table position | 0 mm |
| Inline Composing | Off |

## System - Miscellaneous

| | |
|---|---|
| Positioning mode | REF |
| Table position | H |
| Table position | 0 mm |
| MSMA | S - C - T |
| Sagittal | R >> L |
| Coronal | A >> P |
| Transversal | F >> H |
| Coil Combine Mode | Adaptive Combine |
| Save uncombined | Off |
| Matrix Optimization | Off |
| AutoAlign | --- |
| Coil Select Mode | On - AutoCoilSelect |

## System - Adjustments

| | |
|---|---|
| B0 Shim mode | Standard |
| B1 Shim mode | TrueForm |
| Adjust with body coil | Off |
| Confirm freq. adjustment | Off |
| Assume Dominant Fat | Off |
| Assume Silicone | Off |
| Adjustment Tolerance | Auto |

## System - Adjust Volume

| | |
|---|---|
| Position | R1.2 P36.9 H0.0 mm |
| Orientation | Sagittal |
| Rotation | 90.00 deg |
| F >> H | 128 mm |
| A >> P | 192 mm |
| R >> L | 160 mm |
| Reset | Off |

## System - pTx Volumes

| | |
|---|---|
| B1 Shim mode | TrueForm |
| Excitation | Non-sel. |

## System - Tx/Rx

| | |
|---|---|
| Frequency 1H | 123.244475 MHz |
| Correction factor | 1 |
| Gain | High |
| Img. Scale Cor. | 3.000 |
| Reset | Off |
| ? Ref. amplitude 1H | 0.000 V |

## Physio - Signal1

| | |
|---|---|
| 1st Signal/Mode | None |
| Trigger delay | 0 ms |
| TR | 3200 ms |
| Concatenations | 1 |

## Physio - Cardiac

| | |
|---|---|
| Magn. preparation | None |
| Fat suppr. | Fat sat. |
| Dark blood | Off |
| FoV read | 128 mm |
| FoV phase | 150.0 % |
| Phase resolution | 100 % |

## Physio - PACE

| | |
|---|---|
| Resp. control | Off |
| Concatenations | 1 |

## Inline - Common

| | |
|---|---|
| Subtract | Off |
| Measurements | 1 |
| StdDev | Off |
| Save original images | On |

## Inline - MIP

| | |
|---|---|
| MIP-Sag | Off |
| MIP-Cor | Off |
| MIP-Tra | Off |
| MIP-Time | Off |
| Save original images | On |

## Inline - Composing

| | |
|---|---|
| Inline Composing | Off |
| Distortion Corr. | Off |

## Sequence - Part 1

| | |
|---|---|
| Introduction | On |
| Dimension | 3D |
| Elliptical scanning | Off |
| Reordering | Linear |
| Flow comp. | No |
| Echo spacing | 4.4 ms |
| Adiabatic-mode | Off |
| Bandwidth | 592 Hz/Px |

## Sequence - Part 2

| | |
|---|---|
| Echo train duration | 1034 ms |
| RF pulse type | Low SAR |
| Gradient mode | Whisper |
| Excitation | Non-sel. |
| Flip angle mode | T2 var |
| Turbo factor | 282 |

## Sequence - Assistant

| | |
|---|---|
| Allowed delay | 30 s |

| \\Protocol Development\MJT\Neonate-development\PRENCOG_neonate\DTI_Neonate_v6b_dummy |
|---|
| TA: 0:28 PM: FIX Voxel size: 2.0×2.0×2.0 mmPAT: 4 Rel. SNR: 1.00 : epse |

## Properties

| | |
|---|---|
| Prio recon | Off |
| Load images to viewer | On |
| Inline movie | Off |
| Auto store images | On |
| Load images to stamp segments | Off |
| Load images to graphic segments | Off |
| Auto open inline display | Off |
| Auto close inline display | Off |
| Start measurement without further preparation | Off |
| Wait for user to start | Off |
| Start measurements | Single measurement |

## Routine

| | |
|---|---|
| Slice group | 1 |
| Slices | 58 |
| Dist. factor | 0 % |
| Position | R1.2 P39.7 H47.8 mm |
| Orientation | Transversal |
| Phase enc. dir. | R >> L |
| AutoAlign | --- |
| Phase oversampling | 0 % |
| FoV read | 256 mm |
| FoV phase | 100.0 % |
| Slice thickness | 2.0 mm |
| TR | 3500 ms |
| TE | 78.0 ms |
| Concatenations | 1 |
| Filter | Raw filter, Prescan Normalize |
| Coil elements | PeH;PeN |

## Contrast - Common

| | |
|---|---|
| TR | 3500 ms |
| TE | 78.0 ms |
| MTC | Off |
| Magn. preparation | None |
| Fat suppr. | Fat sat. |
| Fat sat. mode | Strong |

## Contrast - Dynamic

| | |
|---|---|
| Averaging mode | Long term |
| Reconstruction | Magnitude |
| Measurements | 1 |
| Delay in TR | 0 ms |
| Multiple series | Off |

## Resolution - Common

| | |
|---|---|
| FoV read | 256 mm |
| FoV phase | 100.0 % |
| Slice thickness | 2.0 mm |
| Base resolution | 128 |
| Phase resolution | 100 % |
| Phase partial Fourier | 7/8 |
| Interpolation | Off |

## Resolution - iPAT

| | |
|---|---|
| Accel. mode | Slice accel. |
| Accel. factor PE | 2 |
| Ref. lines PE | 40 |

## Resolution - iPAT

| | |
|---|---|
| Accel. factor slice | 2 |
| Reference scan mode | EPI/separate |

## Resolution - Filter Image

| | |
|---|---|
| Distortion Corr. | Off |
| Prescan Normalize | On |
| Dynamic Field Corr. | Off |

## Resolution - Filter Rawdata

| | |
|---|---|
| Raw filter | On |
| Elliptical filter | Off |

## Geometry - Common

| | |
|---|---|
| Slice group | 1 |
| Slices | 58 |
| Dist. factor | 0 % |
| Position | R1.2 P39.7 H47.8 mm |
| Orientation | Transversal |
| Phase enc. dir. | R >> L |
| FoV read | 256 mm |
| FoV phase | 100.0 % |
| Slice thickness | 2.0 mm |
| TR | 3500 ms |
| Multi-slice mode | Interleaved |
| Series | Interleaved |
| Concatenations | 1 |

## Geometry - AutoAlign

| | |
|---|---|
| Slice group | 1 |
| Position | R1.2 P39.7 H47.8 mm |
| Orientation | Transversal |
| Phase enc. dir. | R >> L |
| AutoAlign | --- |
| Initial Position | R1.2 P39.7 H47.8 |
| R | 1.2 mm |
| P | 39.7 mm |
| H | 47.8 mm |
| Initial Rotation | 90.00 deg |
| Initial Orientation | Transversal |

## Geometry - Saturation

| | |
|---|---|
| Fat suppr. | Fat sat. |
| Fat sat. mode | Strong |
| Special sat. | None |

## Geometry - Navigator

## Geometry - Tim Planning Suite

| | |
|---|---|
| Set-n-Go Protocol | Off |
| Table position | H |
| Table position | 0 mm |
| Inline Composing | Off |

## System - Miscellaneous

| | |
|---|---|
| Positioning mode | FIX |
| Table position | H |
| Table position | 0 mm |
| MSMA | S - C - T |
| Sagittal | R >> L |

## System - Miscellaneous

| | |
|---|---|
| Coronal | A >> P |
| Transversal | F >> H |
| Coil Combine Mode | Adaptive Combine |
| Matrix Optimization | Performance |
| AutoAlign | --- |
| Coil Select Mode | On - AutoCoilSelect |

## System - Adjustments

| | |
|---|---|
| B0 Shim mode | Standard |
| B1 Shim mode | TrueForm |
| Adjust with body coil | Off |
| Confirm freq. adjustment | Off |
| Assume Dominant Fat | Off |
| Assume Silicone | Off |
| Adjustment Tolerance | Auto |

## System - Adjust Volume

| | |
|---|---|
| Position | R1.2 P39.7 H47.8 mm |
| Orientation | Transversal |
| Rotation | 90.00 deg |
| R >> L | 256 mm |
| A >> P | 256 mm |
| F >> H | 116 mm |
| Reset | Off |

## System - pTx Volumes

| | |
|---|---|
| B1 Shim mode | TrueForm |
| Excitation | Standard |

## System - Tx/Rx

| | |
|---|---|
| Frequency 1H | 123.244475 MHz |
| Correction factor | 1 |
| Gain | High |
| Img. Scale Cor. | 1.000 |
| Reset | Off |
| ? Ref. amplitude 1H | 0.000 V |

## Physio - Signal1

| | |
|---|---|
| 1st Signal/Mode | None |
| TR | 3500 ms |
| Concatenations | 1 |

## Physio - PACE

| | |
|---|---|
| Resp. control | Off |
| Concatenations | 1 |

## Diff - Neuro

| | |
|---|---|
| Diffusion mode | Free |
| Diff. directions | 71 |
| Diffusion Scheme | Monopolar |
| Diff. weightings | 1 |
| b-value | 0 s/mm² |
| b-value | 3 |
| Diff. weighted images | On |
| Trace weighted images | Off |
| ADC maps | Off |
| FA maps | Off |
| Mosaic | Off |
| Tensor | Off |
| Noise level | 40 |

## Diff - Body

| | |
|---|---|
| Diffusion mode | Free |

## Diff - Body

| | |
|---|---|
| Diff. directions | 71 |
| Diffusion Scheme | Monopolar |
| Diff. weightings | 1 |
| b-value | 0 s/mm² |
| b-value | 3 |
| Diff. weighted images | On |
| Trace weighted images | Off |
| ADC maps | Off |
| Exponential ADC Maps | Off |
| FA maps | Off |
| Invert Gray Scale | Off |
| Calculated Image | Off |
| b-Value >= | 0 s/mm² |
| Noise level | 40 |

## Diff - Composing

| | |
|---|---|
| Inline Composing | Off |
| Distortion Corr. | Off |

## Sequence - Part 1

| | |
|---|---|
| Introduction | Off |
| Optimization | None |
| Multi-slice mode | Interleaved |
| Free echo spacing | Off |
| Echo spacing | 0.78 ms |
| Bandwidth | 1446 Hz/Px |

## Sequence - Part 2

| | |
|---|---|
| EPI factor | 128 |
| RF pulse type | Low SAR |
| Gradient mode | Normal |
| Excitation | Standard |

## Sequence - pTX Pulses

| \\Protocol Development\MJT\Neonate-development\PRENCOG_neonate\DTI_Neonate_v6b_rev |
|---|
| TA: 0:28 PM: FIX Voxel size: 2.0×2.0×2.0 mmPAT: 4 Rel. SNR: 1.00 : epse |

## Properties

| | |
|---|---|
| Prio recon | Off |
| Load images to viewer | On |
| Inline movie | Off |
| Auto store images | On |
| Load images to stamp segments | Off |
| Load images to graphic segments | Off |
| Auto open inline display | Off |
| Auto close inline display | Off |
| Start measurement without further preparation | Off |
| Wait for user to start | Off |
| Start measurements | Single measurement |

## Routine

| | |
|---|---|
| Slice group | 1 |
| Slices | 58 |
| Dist. factor | 0 % |
| Position | R1.2 P39.7 H47.8 mm |
| Orientation | Transversal |
| Phase enc. dir. | R >> L |
| AutoAlign | --- |
| Phase oversampling | 0 % |
| FoV read | 256 mm |
| FoV phase | 100.0 % |
| Slice thickness | 2.0 mm |
| TR | 3500 ms |
| TE | 78.0 ms |
| Concatenations | 1 |
| Filter | Raw filter, Prescan Normalize |
| Coil elements | PeH;PeN |

## Contrast - Common

| | |
|---|---|
| TR | 3500 ms |
| TE | 78.0 ms |
| MTC | Off |
| Magn. preparation | None |
| Fat suppr. | Fat sat. |
| Fat sat. mode | Strong |

## Contrast - Dynamic

| | |
|---|---|
| Averaging mode | Long term |
| Reconstruction | Magnitude |
| Measurements | 1 |
| Delay in TR | 0 ms |
| Multiple series | Off |

## Resolution - Common

| | |
|---|---|
| FoV read | 256 mm |
| FoV phase | 100.0 % |
| Slice thickness | 2.0 mm |
| Base resolution | 128 |
| Phase resolution | 100 % |
| Phase partial Fourier | 7/8 |
| Interpolation | Off |

## Resolution - iPAT

| | |
|---|---|
| Accel. mode | Slice accel. |
| Accel. factor PE | 2 |
| Ref. lines PE | 40 |

## Resolution - iPAT

| | |
|---|---|
| Accel. factor slice | 2 |
| Reference scan mode | EPI/separate |

## Resolution - Filter Image

| | |
|---|---|
| Distortion Corr. | Off |
| Prescan Normalize | On |
| Dynamic Field Corr. | Off |

## Resolution - Filter Rawdata

| | |
|---|---|
| Raw filter | On |
| Elliptical filter | Off |

## Geometry - Common

| | |
|---|---|
| Slice group | 1 |
| Slices | 58 |
| Dist. factor | 0 % |
| Position | R1.2 P39.7 H47.8 mm |
| Orientation | Transversal |
| Phase enc. dir. | R >> L |
| FoV read | 256 mm |
| FoV phase | 100.0 % |
| Slice thickness | 2.0 mm |
| TR | 3500 ms |
| Multi-slice mode | Interleaved |
| Series | Interleaved |
| Concatenations | 1 |

## Geometry - AutoAlign

| | |
|---|---|
| Slice group | 1 |
| Position | R1.2 P39.7 H47.8 mm |
| Orientation | Transversal |
| Phase enc. dir. | R >> L |
| AutoAlign | --- |
| Initial Position | R1.2 P39.7 H47.8 |
| R | 1.2 mm |
| P | 39.7 mm |
| H | 47.8 mm |
| Initial Rotation | 90.00 deg |
| Initial Orientation | Transversal |

## Geometry - Saturation

| | |
|---|---|
| Fat suppr. | Fat sat. |
| Fat sat. mode | Strong |
| Special sat. | None |

## Geometry - Navigator

## Geometry - Tim Planning Suite

| | |
|---|---|
| Set-n-Go Protocol | Off |
| Table position | H |
| Table position | 0 mm |
| Inline Composing | Off |

## System - Miscellaneous

| | |
|---|---|
| Positioning mode | FIX |
| Table position | H |
| Table position | 0 mm |
| MSMA | S - C - T |
| Sagittal | R >> L |

## System - Miscellaneous

| | |
|---|---|
| Coronal | A >> P |
| Transversal | F >> H |
| Coil Combine Mode | Adaptive Combine |
| Matrix Optimization | Performance |
| AutoAlign | --- |
| Coil Select Mode | On - AutoCoilSelect |

## System - Adjustments

| | |
|---|---|
| B0 Shim mode | Standard |
| B1 Shim mode | TrueForm |
| Adjust with body coil | Off |
| Confirm freq. adjustment | Off |
| Assume Dominant Fat | Off |
| Assume Silicone | Off |
| Adjustment Tolerance | Auto |

## System - Adjust Volume

| | |
|---|---|
| Position | R1.2 P39.7 H47.8 mm |
| Orientation | Transversal |
| Rotation | 90.00 deg |
| R >> L | 256 mm |
| A >> P | 256 mm |
| F >> H | 116 mm |
| Reset | Off |

## System - pTx Volumes

| | |
|---|---|
| B1 Shim mode | TrueForm |
| Excitation | Standard |

## System - Tx/Rx

| | |
|---|---|
| Frequency 1H | 123.244475 MHz |
| Correction factor | 1 |
| Gain | High |
| Img. Scale Cor. | 1.000 |
| Reset | Off |
| ? Ref. amplitude 1H | 0.000 V |

## Physio - Signal1

| | |
|---|---|
| 1st Signal/Mode | None |
| TR | 3500 ms |
| Concatenations | 1 |

## Physio - PACE

| | |
|---|---|
| Resp. control | Off |
| Concatenations | 1 |

## Diff - Neuro

| | |
|---|---|
| Diffusion mode | MDDW |
| Diff. directions | 6 |
| Diffusion Scheme | Monopolar |
| Diff. weightings | 1 |
| b-value | 0 s/mm² |
| b-value | 3 |
| Diff. weighted images | On |
| Trace weighted images | Off |
| ADC maps | Off |
| FA maps | Off |
| Mosaic | Off |
| Tensor | Off |
| Noise level | 40 |

## Diff - Body

| | |
|---|---|
| Diffusion mode | MDDW |

## Diff - Body

| | |
|---|---|
| Diff. directions | 6 |
| Diffusion Scheme | Monopolar |
| Diff. weightings | 1 |
| b-value | 0 s/mm² |
| b-value | 3 |
| Diff. weighted images | On |
| Trace weighted images | Off |
| ADC maps | Off |
| Exponential ADC Maps | Off |
| FA maps | Off |
| Invert Gray Scale | Off |
| Calculated Image | Off |
| b-Value >= | 0 s/mm² |
| Noise level | 40 |

## Diff - Composing

| | |
|---|---|
| Inline Composing | Off |
| Distortion Corr. | Off |

## Sequence - Part 1

| | |
|---|---|
| Introduction | Off |
| Optimization | None |
| Multi-slice mode | Interleaved |
| Free echo spacing | Off |
| Echo spacing | 0.78 ms |
| Bandwidth | 1446 Hz/Px |

## Sequence - Part 2

| | |
|---|---|
| EPI factor | 128 |
| RF pulse type | Low SAR |
| Gradient mode | Normal |
| Excitation | Standard |

## Sequence - pTX Pulses

| \\Protocol Development\MJT\Neonate-development\PRENCOG_neonate\DTI_Neonate_v6b_pt1 |
|---|
| TA: 4:29 PM: FIX Voxel size: 2.0×2.0×2.0 mmPAT: 4 Rel. SNR: 1.00 : epse |

## Properties

| | |
|---|---|
| Prio recon | Off |
| Load images to viewer | On |
| Inline movie | Off |
| Auto store images | On |
| Load images to stamp segments | Off |
| Load images to graphic segments | Off |
| Auto open inline display | Off |
| Auto close inline display | Off |
| Start measurement without further preparation | Off |
| Wait for user to start | Off |
| Start measurements | Single measurement |

## Routine

| | |
|---|---|
| Slice group | 1 |
| Slices | 58 |
| Dist. factor | 0 % |
| Position | R1.2 P39.7 H47.8 mm |
| Orientation | Transversal |
| Phase enc. dir. | L >> R |
| AutoAlign | --- |
| Phase oversampling | 0 % |
| FoV read | 256 mm |
| FoV phase | 100.0 % |
| Slice thickness | 2.0 mm |
| TR | 3500 ms |
| TE | 78.0 ms |
| Averages | 1 |
| Concatenations | 1 |
| Filter | Raw filter, Prescan Normalize |
| Coil elements | PeH;PeN |

## Contrast - Common

| | |
|---|---|
| TR | 3500 ms |
| TE | 78.0 ms |
| MTC | Off |
| Magn. preparation | None |
| Fat suppr. | Fat sat. |
| Fat sat. mode | Strong |

## Contrast - Dynamic

| | |
|---|---|
| Averages | 1 |
| Averaging mode | Long term |
| Reconstruction | Magnitude |
| Measurements | 1 |
| Delay in TR | 0 ms |
| Multiple series | Off |

## Resolution - Common

| | |
|---|---|
| FoV read | 256 mm |
| FoV phase | 100.0 % |
| Slice thickness | 2.0 mm |
| Base resolution | 128 |
| Phase resolution | 100 % |
| Phase partial Fourier | 7/8 |
| Interpolation | Off |

## Resolution - iPAT

| | |
|---|---|
| Accel. mode | Slice accel. |

## Resolution - iPAT

| | |
|---|---|
| Accel. factor PE | 2 |
| Ref. lines PE | 40 |
| Accel. factor slice | 2 |
| Reference scan mode | EPI/separate |

## Resolution - Filter Image

| | |
|---|---|
| Distortion Corr. | Off |
| Prescan Normalize | On |
| Dynamic Field Corr. | Off |

## Resolution - Filter Rawdata

| | |
|---|---|
| Raw filter | On |
| Elliptical filter | Off |

## Geometry - Common

| | |
|---|---|
| Slice group | 1 |
| Slices | 58 |
| Dist. factor | 0 % |
| Position | R1.2 P39.7 H47.8 mm |
| Orientation | Transversal |
| Phase enc. dir. | L >> R |
| FoV read | 256 mm |
| FoV phase | 100.0 % |
| Slice thickness | 2.0 mm |
| TR | 3500 ms |
| Multi-slice mode | Interleaved |
| Series | Interleaved |
| Concatenations | 1 |

## Geometry - AutoAlign

| | |
|---|---|
| Slice group | 1 |
| Position | R1.2 P39.7 H47.8 mm |
| Orientation | Transversal |
| Phase enc. dir. | L >> R |
| AutoAlign | --- |
| Initial Position | R1.2 P39.7 H47.8 |
| R | 1.2 mm |
| P | 39.7 mm |
| H | 47.8 mm |
| Initial Rotation | -90.00 deg |
| Initial Orientation | Transversal |

## Geometry - Saturation

| | |
|---|---|
| Fat suppr. | Fat sat. |
| Fat sat. mode | Strong |
| Special sat. | None |

## Geometry - Navigator

## Geometry - Tim Planning Suite

| | |
|---|---|
| Set-n-Go Protocol | Off |
| Table position | H |
| Table position | 0 mm |
| Inline Composing | Off |

## System - Miscellaneous

| | |
|---|---|
| Positioning mode | FIX |
| Table position | H |
| Table position | 0 mm |

## System - Miscellaneous

| | |
|---|---|
| MSMA | S - C - T |
| Sagittal | R >> L |
| Coronal | A >> P |
| Transversal | F >> H |
| Coil Combine Mode | Adaptive Combine |
| Matrix Optimization | Performance |
| AutoAlign | --- |
| Coil Select Mode | On - AutoCoilSelect |

## System - Adjustments

| | |
|---|---|
| B0 Shim mode | Standard |
| B1 Shim mode | TrueForm |
| Adjust with body coil | Off |
| Confirm freq. adjustment | Off |
| Assume Dominant Fat | Off |
| Assume Silicone | Off |
| Adjustment Tolerance | Auto |

## System - Adjust Volume

| | |
|---|---|
| Position | R1.2 P39.7 H47.8 mm |
| Orientation | Transversal |
| Rotation | -90.00 deg |
| R >> L | 256 mm |
| A >> P | 256 mm |
| F >> H | 116 mm |
| Reset | Off |

## System - pTx Volumes

| | |
|---|---|
| B1 Shim mode | TrueForm |
| Excitation | Standard |

## System - Tx/Rx

| | |
|---|---|
| Frequency 1H | 123.244475 MHz |
| Correction factor | 1 |
| Gain | High |
| Img. Scale Cor. | 1.000 |
| Reset | Off |
| ? Ref. amplitude 1H | 0.000 V |

## Physio - Signal1

| | |
|---|---|
| 1st Signal/Mode | None |
| TR | 3500 ms |
| Concatenations | 1 |

## Physio - PACE

| | |
|---|---|
| Resp. control | Off |
| Concatenations | 1 |

## Diff - Neuro

| | |
|---|---|
| Diffusion mode | Free |
| Diff. directions | 71 |
| Diffusion Scheme | Monopolar |
| Diff. weightings | 2 |
| b-value 1 | 0 s/mm² |
| b-value 2 | 750 s/mm² |
| b-value 1 | 1 |
| b-value 2 | 1 |
| Diff. weighted images | On |
| Trace weighted images | Off |
| ADC maps | Off |
| FA maps | Off |
| Mosaic | On |
| Tensor | Off |

## Diff - Neuro

| | |
|---|---|
| Noise level | 40 |

## Diff - Body

| | |
|---|---|
| Diffusion mode | Free |
| Diff. directions | 71 |
| Diffusion Scheme | Monopolar |
| Diff. weightings | 2 |
| b-value 1 | 0 s/mm² |
| b-value 2 | 750 s/mm² |
| b-value 1 | 1 |
| b-value 2 | 1 |
| Diff. weighted images | On |
| Trace weighted images | Off |
| ADC maps | Off |
| Exponential ADC Maps | Off |
| FA maps | Off |
| Invert Gray Scale | Off |
| Calculated Image | Off |
| b-Value >= | 0 s/mm² |
| Noise level | 40 |

## Diff - Composing

| | |
|---|---|
| Inline Composing | Off |
| Distortion Corr. | Off |

## Sequence - Part 1

| | |
|---|---|
| Introduction | Off |
| Optimization | None |
| Multi-slice mode | Interleaved |
| Free echo spacing | Off |
| Echo spacing | 0.78 ms |
| Bandwidth | 1446 Hz/Px |

## Sequence - Part 2

| | |
|---|---|
| EPI factor | 128 |
| RF pulse type | Low SAR |
| Gradient mode | Normal |
| Excitation | Standard |

## Sequence - pTX Pulses

| \\Protocol Development\MJT\Neonate-development\PRENCOG_neonate\DTI_Neonate_v6b_pt2 |
|---|
| TA: 5:01 PM: FIX Voxel size: 2.0×2.0×2.0 mmPAT: 4 Rel. SNR: 1.00 : epse |

## Properties

| | |
|---|---|
| Prio recon | Off |
| Load images to viewer | On |
| Inline movie | Off |
| Auto store images | On |
| Load images to stamp segments | Off |
| Load images to graphic segments | Off |
| Auto open inline display | Off |
| Auto close inline display | Off |
| Start measurement without further preparation | Off |
| Wait for user to start | Off |
| Start measurements | Single measurement |

## Routine

| | |
|---|---|
| Slice group | 1 |
| Slices | 58 |
| Dist. factor | 0 % |
| Position | R1.2 P39.7 H47.8 mm |
| Orientation | Transversal |
| Phase enc. dir. | L >> R |
| AutoAlign | --- |
| Phase oversampling | 0 % |
| FoV read | 256 mm |
| FoV phase | 100.0 % |
| Slice thickness | 2.0 mm |
| TR | 3500 ms |
| TE | 78.0 ms |
| Averages | 1 |
| Concatenations | 1 |
| Filter | Raw filter, Prescan Normalize |
| Coil elements | PeH;PeN |

## Contrast - Common

| | |
|---|---|
| TR | 3500 ms |
| TE | 78.0 ms |
| MTC | Off |
| Magn. preparation | None |
| Fat suppr. | Fat sat. |
| Fat sat. mode | Strong |

## Contrast - Dynamic

| | |
|---|---|
| Averages | 1 |
| Averaging mode | Long term |
| Reconstruction | Magnitude |
| Measurements | 1 |
| Delay in TR | 0 ms |
| Multiple series | Off |

## Resolution - Common

| | |
|---|---|
| FoV read | 256 mm |
| FoV phase | 100.0 % |
| Slice thickness | 2.0 mm |
| Base resolution | 128 |
| Phase resolution | 100 % |
| Phase partial Fourier | 7/8 |
| Interpolation | Off |

## Resolution - iPAT

| | |
|---|---|
| Accel. mode | Slice accel. |
| Accel. factor PE | 2 |
| Ref. lines PE | 40 |
| Accel. factor slice | 2 |
| Reference scan mode | EPI/separate |

## Resolution - Filter Image

| | |
|---|---|
| Distortion Corr. | Off |
| Prescan Normalize | On |
| Dynamic Field Corr. | Off |

## Resolution - Filter Rawdata

| | |
|---|---|
| Raw filter | On |
| Elliptical filter | Off |

## Geometry - Common

| | |
|---|---|
| Slice group | 1 |
| Slices | 58 |
| Dist. factor | 0 % |
| Position | R1.2 P39.7 H47.8 mm |
| Orientation | Transversal |
| Phase enc. dir. | L >> R |
| FoV read | 256 mm |
| FoV phase | 100.0 % |
| Slice thickness | 2.0 mm |
| TR | 3500 ms |
| Multi-slice mode | Interleaved |
| Series | Interleaved |
| Concatenations | 1 |

## Geometry - AutoAlign

| | |
|---|---|
| Slice group | 1 |
| Position | R1.2 P39.7 H47.8 mm |
| Orientation | Transversal |
| Phase enc. dir. | L >> R |
| AutoAlign | --- |
| Initial Position | R1.2 P39.7 H47.8 |
| R | 1.2 mm |
| P | 39.7 mm |
| H | 47.8 mm |
| Initial Rotation | -90.00 deg |
| Initial Orientation | Transversal |

## Geometry - Saturation

| | |
|---|---|
| Fat suppr. | Fat sat. |
| Fat sat. mode | Strong |
| Special sat. | None |

## Geometry - Navigator

## Geometry - Tim Planning Suite

| | |
|---|---|
| Set-n-Go Protocol | Off |
| Table position | H |
| Table position | 0 mm |
| Inline Composing | Off |

## System - Miscellaneous

| | |
|---|---|
| Positioning mode | FIX |
| Table position | H |
| Table position | 0 mm |

## System - Miscellaneous

| | |
|---|---|
| MSMA | S - C - T |
| Sagittal | R >> L |
| Coronal | A >> P |
| Transversal | F >> H |
| Coil Combine Mode | Adaptive Combine |
| Matrix Optimization | Performance |
| AutoAlign | --- |
| Coil Select Mode | On - AutoCoilSelect |

## System - Adjustments

| | |
|---|---|
| B0 Shim mode | Standard |
| B1 Shim mode | TrueForm |
| Adjust with body coil | Off |
| Confirm freq. adjustment | Off |
| Assume Dominant Fat | Off |
| Assume Silicone | Off |
| Adjustment Tolerance | Auto |

## System - Adjust Volume

| | |
|---|---|
| Position | R1.2 P39.7 H47.8 mm |
| Orientation | Transversal |
| Rotation | -90.00 deg |
| R >> L | 256 mm |
| A >> P | 256 mm |
| F >> H | 116 mm |
| Reset | Off |

## System - pTx Volumes

| | |
|---|---|
| B1 Shim mode | TrueForm |
| Excitation | Standard |

## System - Tx/Rx

| | |
|---|---|
| Frequency 1H | 123.244475 MHz |
| Correction factor | 1 |
| Gain | High |
| Img. Scale Cor. | 1.000 |
| Reset | Off |
| ? Ref. amplitude 1H | 0.000 V |

## Physio - Signal1

| | |
|---|---|
| 1st Signal/Mode | None |
| TR | 3500 ms |
| Concatenations | 1 |

## Physio - PACE

| | |
|---|---|
| Resp. control | Off |
| Concatenations | 1 |

## Diff - Neuro

| | |
|---|---|
| Diffusion mode | Free |
| Diff. directions | 80 |
| Diffusion Scheme | Monopolar |
| Diff. weightings | 2 |
| b-value 1 | 0 s/mm² |
| b-value 2 | 2500 s/mm² |
| b-value 1 | 1 |
| b-value 2 | 1 |
| Diff. weighted images | On |
| Trace weighted images | Off |
| ADC maps | Off |
| FA maps | Off |
| Mosaic | On |
| Tensor | Off |

## Diff - Neuro

| | |
|---|---|
| Noise level | 40 |

## Diff - Body

| | |
|---|---|
| Diffusion mode | Free |
| Diff. directions | 80 |
| Diffusion Scheme | Monopolar |
| Diff. weightings | 2 |
| b-value 1 | 0 s/mm² |
| b-value 2 | 2500 s/mm² |
| b-value 1 | 1 |
| b-value 2 | 1 |
| Diff. weighted images | On |
| Trace weighted images | Off |
| ADC maps | Off |
| Exponential ADC Maps | Off |
| FA maps | Off |
| Invert Gray Scale | Off |
| Calculated Image | Off |
| b-Value >= | 0 s/mm² |
| Noise level | 40 |

## Diff - Composing

| | |
|---|---|
| Inline Composing | Off |
| Distortion Corr. | Off |

## Sequence - Part 1

| | |
|---|---|
| Introduction | Off |
| Optimization | None |
| Multi-slice mode | Interleaved |
| Free echo spacing | Off |
| Echo spacing | 0.78 ms |
| Bandwidth | 1446 Hz/Px |

## Sequence - Part 2

| | |
|---|---|
| EPI factor | 128 |
| RF pulse type | Low SAR |
| Gradient mode | Normal |
| Excitation | Standard |

## Sequence - pTX Pulses

**\\Protocol Development\MJT\Neonate-development\PRENCOG_neonate\MPRAGE-v4**

TA: 3:09 PM: FIX Voxel size: 1.0×1.0×1.0 mmPAT: 2 Rel. SNR: 1.00 : tfl

## Properties

| | |
|---|---|
| Prio recon | Off |
| Load images to viewer | On |
| Inline movie | Off |
| Auto store images | On |
| Load images to stamp segments | On |
| Load images to graphic segments | Off |
| Auto open inline display | Off |
| Auto close inline display | Off |
| Start measurement without further preparation | Off |
| Wait for user to start | Off |
| Start measurements | Single measurement |

## Routine

| | |
|---|---|
| Slab group | 1 |
| Slabs | 1 |
| Dist. factor | 50 % |
| Position | R1.1 P38.9 F20.7 mm |
| Orientation | Sagittal |
| Phase enc. dir. | A >> P |
| AutoAlign | --- |
| Phase oversampling | 20 % |
| Slice oversampling | 0.0 % |
| Slices per slab | 160 |
| FoV read | 160 mm |
| FoV phase | 100.0 % |
| Slice thickness | 1.00 mm |
| TR | 1970.0 ms |
| TE | 4.69 ms |
| Averages | 1 |
| Concatenations | 1 |
| Filter | Prescan Normalize |
| Coil elements | PeH;PeN;SP1 |

## Contrast - Common

| | |
|---|---|
| TR | 1970.0 ms |
| TE | 4.69 ms |
| Magn. preparation | Non-sel. IR |
| TI | 1100 ms |
| Flip angle | 9 deg |
| Fat suppr. | None |
| Water suppr. | None |

## Contrast - Dynamic

| | |
|---|---|
| Averages | 1 |
| Averaging mode | Long term |
| Reconstruction | Magnitude |
| Measurements | 1 |
| Multiple series | Each measurement |

## Resolution - Common

| | |
|---|---|
| FoV read | 160 mm |
| FoV phase | 100.0 % |
| Slice thickness | 1.00 mm |
| Base resolution | 160 |
| Phase resolution | 100 % |
| Slice resolution | 100 % |
| Phase partial Fourier | 7/8 |
| Slice partial Fourier | Off |
| Interpolation | Off |

## Resolution - iPAT

| | |
|---|---|
| PAT mode | GRAPPA |
| Accel. factor PE | 2 |
| Ref. lines PE | 24 |
| Accel. factor 3D | 1 |
| Reference scan mode | Integrated |

## Resolution - Filter Image

| | |
|---|---|
| Image Filter | Off |
| Distortion Corr. | Off |
| Prescan Normalize | On |
| Unfiltered images | Off |
| Normalize | Off |
| B1 filter | Off |

## Resolution - Filter Rawdata

| | |
|---|---|
| Raw filter | Off |
| Elliptical filter | Off |

## Geometry - Common

| | |
|---|---|
| Slab group | 1 |
| Slabs | 1 |
| Dist. factor | 50 % |
| Position | R1.1 P38.9 F20.7 mm |
| Orientation | Sagittal |
| Phase enc. dir. | A >> P |
| Slice oversampling | 0.0 % |
| Slices per slab | 160 |
| FoV read | 160 mm |
| FoV phase | 100.0 % |
| Slice thickness | 1.00 mm |
| TR | 1970.0 ms |
| Multi-slice mode | Single shot |
| Series | Interleaved |
| Concatenations | 1 |

## Geometry - AutoAlign

| | |
|---|---|
| Slab group | 1 |
| Position | R1.1 P38.9 F20.7 mm |
| Orientation | Sagittal |
| Phase enc. dir. | A >> P |
| AutoAlign | --- |
| Initial Position | R1.1 P38.9 F20.7 |
| R | 1.1 mm |
| P | 38.9 mm |
| F | 20.7 mm |
| Initial Rotation | 0.00 deg |
| Initial Orientation | Sagittal |

## Geometry - Navigator

## Geometry - Tim Planning Suite

| | |
|---|---|
| Set-n-Go Protocol | Off |
| Table position | H |
| Table position | 0 mm |
| Inline Composing | Off |

## System - Miscellaneous

| | |
|---|---|
| Positioning mode | FIX |
| Table position | H |
| Table position | 0 mm |

## System - Miscellaneous

| | |
|---|---|
| MSMA | S - C - T |
| Sagittal | R >> L |
| Coronal | A >> P |
| Transversal | F >> H |
| Coil Combine Mode | Adaptive Combine |
| Save uncombined | Off |
| Matrix Optimization | Off |
| Coil Focus | Flat |
| AutoAlign | --- |
| Coil Select Mode | On - AutoCoilSelect |

## System - Adjustments

| | |
|---|---|
| B0 Shim mode | Standard |
| B1 Shim mode | TrueForm |
| Adjust with body coil | Off |
| Confirm freq. adjustment | Off |
| Assume Dominant Fat | Off |
| Assume Silicone | Off |
| Adjustment Tolerance | Auto |

## System - Adjust Volume

| | |
|---|---|
| Position | R1.1 P38.9 F20.7 mm |
| Orientation | Sagittal |
| Rotation | 0.00 deg |
| A >> P | 160 mm |
| F >> H | 160 mm |
| R >> L | 160 mm |
| Reset | Off |

## System - pTx Volumes

| | |
|---|---|
| B1 Shim mode | TrueForm |
| Excitation | Non-sel. |

## System - Tx/Rx

| | |
|---|---|
| Frequency 1H | 123.244475 MHz |
| Correction factor | 1 |
| Gain | Low |
| Img. Scale Cor. | 4.000 |
| Reset | Off |
| ? Ref. amplitude 1H | 0.000 V |

## Physio - Signal1

| | |
|---|---|
| 1st Signal/Mode | None |
| TR | 1970.0 ms |
| Concatenations | 1 |

## Physio - Cardiac

| | |
|---|---|
| Magn. preparation | Non-sel. IR |
| TI | 1100 ms |
| Fat suppr. | None |
| Dark blood | Off |
| FoV read | 160 mm |
| FoV phase | 100.0 % |
| Phase resolution | 100 % |

## Physio - PACE

| | |
|---|---|
| Resp. control | Off |
| Concatenations | 1 |

## Inline - Common

| | |
|---|---|
| Subtract | Off |
| Measurements | 1 |
| StdDev | Off |

## Inline - Common

| | |
|---|---|
| Save original images | On |

## Inline - MIP

| | |
|---|---|
| MIP-Sag | Off |
| MIP-Cor | Off |
| MIP-Tra | Off |
| MIP-Time | Off |
| Save original images | On |

## Inline - Composing

| | |
|---|---|
| Inline Composing | Off |
| Distortion Corr. | Off |

## Inline - MapIt

| | |
|---|---|
| Save original images | On |
| MapIt | None |
| Flip angle | 9 deg |
| Measurements | 1 |
| TR | 1970.0 ms |
| TE | 4.69 ms |

## Sequence - Part 1

| | |
|---|---|
| Introduction | On |
| Dimension | 3D |
| Elliptical scanning | Off |
| Reordering | Linear |
| Asymmetric echo | Off |
| Flow comp. | No |
| Multi-slice mode | Single shot |
| Echo spacing | 10.8 ms |
| Bandwidth | 140 Hz/Px |

## Sequence - Part 2

| | |
|---|---|
| RF pulse type | Normal |
| Gradient mode | Whisper |
| Excitation | Non-sel. |
| RF spoiling | On |
| Incr. Gradient spoiling | Off |
| Turbo factor | 160 |

## Sequence - Assistant

| | |
|---|---|
| Mode | Off |

| \\Protocol Development\MJT\Neonate-development\PRENCOG_neonate\B1Map_tra |
|---|
| TA: 0:19 PM: FIX Voxel size: 2.6×2.6×3.0 mmPAT: Off Rel. SNR: 1.00 : tfl |

## Properties

| | |
|---|---|
| Prio recon | Off |
| Load images to viewer | On |
| Inline movie | Off |
| Auto store images | On |
| Load images to stamp segments | Off |
| Load images to graphic segments | Off |
| Auto open inline display | Off |
| Auto close inline display | Off |
| Start measurement without further preparation | Off |
| Wait for user to start | Off |
| Start measurements | Single measurement |

## Routine

| | |
|---|---|
| Slice group | 1 |
| Slices | 31 |
| Dist. factor | 50 % |
| Position | R0.6 P2.2 H11.6 mm |
| Orientation | Transversal |
| Phase enc. dir. | R >> L |
| AutoAlign | Head > Brain |
| Phase oversampling | 0 % |
| FoV read | 166 mm |
| FoV phase | 93.8 % |
| Slice thickness | 3.0 mm |
| TR | 9500.0 ms |
| TE | 2.5 ms |
| Averages | 1 |
| Concatenations | 1 |
| Filter | Prescan Normalize |
| Coil elements | HEA;HEP |

## Contrast - Common

| | |
|---|---|
| TR | 9500.0 ms |
| TE | 2.5 ms |
| Magn. preparation | None |
| Flip angle | 8 deg |
| Fat suppr. | None |
| Water suppr. | None |

## Contrast - Dynamic

| | |
|---|---|
| Averages | 1 |
| Reconstruction | Magnitude |
| Measurements | 1 |
| Multiple series | Each measurement |

## Resolution - Common

| | |
|---|---|
| FoV read | 166 mm |
| FoV phase | 93.8 % |
| Slice thickness | 3.0 mm |
| Base resolution | 64 |
| Phase resolution | 100 % |
| Phase partial Fourier | Off |
| Interpolation | Off |

## Resolution - iPAT

| | |
|---|---|
| PAT mode | None |

## Resolution - Filter Image

| | |
|---|---|
| Image Filter | Off |

## Resolution - Filter Image

| | |
|---|---|
| Distortion Corr. | Off |
| Prescan Normalize | On |
| Unfiltered images | Off |
| Normalize | Off |
| B1 filter | Off |

## Resolution - Filter Rawdata

| | |
|---|---|
| Raw filter | Off |
| Elliptical filter | Off |

## Geometry - Common

| | |
|---|---|
| Slice group | 1 |
| Slices | 31 |
| Dist. factor | 50 % |
| Position | R0.6 P2.2 H11.6 mm |
| Orientation | Transversal |
| Phase enc. dir. | R >> L |
| FoV read | 166 mm |
| FoV phase | 93.8 % |
| Slice thickness | 3.0 mm |
| TR | 9500.0 ms |
| Multi-slice mode | Interleaved |
| Series | Interleaved |
| Concatenations | 1 |

## Geometry - AutoAlign

| | |
|---|---|
| Slice group | 1 |
| Position | R0.6 P2.2 H11.6 mm |
| Orientation | Transversal |
| Phase enc. dir. | R >> L |
| AutoAlign | Head > Brain |
| Initial Position | R0.6 P2.2 H11.6 |
| R | 0.6 mm |
| P | 2.2 mm |
| H | 11.6 mm |
| Initial Rotation | 90.00 deg |
| Initial Orientation | Transversal |

## Geometry - Tim Planning Suite

| | |
|---|---|
| Set-n-Go Protocol | Off |
| Table position | H |
| Table position | 0 mm |
| Inline Composing | Off |

## System - Miscellaneous

| | |
|---|---|
| Positioning mode | FIX |
| Table position | H |
| Table position | 0 mm |
| MSMA | S - C - T |
| Sagittal | R >> L |
| Coronal | A >> P |
| Transversal | F >> H |
| Coil Combine Mode | Sum of Squares |
| Save uncombined | Off |
| Matrix Optimization | Off |
| AutoAlign | Head > Brain |
| Coil Select Mode | Default |

## System - Adjustments

| | |
|---|---|
| B0 Shim mode | Standard |

## System - Adjustments

| | |
|---|---|
| B1 Shim mode | TrueForm |
| Adjust with body coil | Off |
| Confirm freq. adjustment | Off |
| Assume Dominant Fat | Off |
| Assume Silicone | Off |
| Adjustment Tolerance | Auto |

## System - Adjust Volume

| | |
|---|---|
| Position | R0.6 P2.2 H11.6 mm |
| Orientation | Transversal |
| Rotation | 90.00 deg |
| R >> L | 156 mm |
| A >> P | 166 mm |
| F >> H | 138 mm |
| Reset | Off |

## System - pTx Volumes

| | |
|---|---|
| B1 Shim mode | TrueForm |
| Excitation | Slice-sel. |

## System - Tx/Rx

| | |
|---|---|
| Frequency 1H | 123.244475 MHz |
| Correction factor | 1 |
| Gain | High |
| Img. Scale Cor. | 1.000 |
| Reset | Off |
| ? Ref. amplitude 1H | 0.000 V |

## Inline - Common

| | |
|---|---|
| Subtract | Off |
| Measurements | 1 |
| StdDev | Off |
| Save original images | On |

## Inline - MIP

| | |
|---|---|
| MIP-Sag | Off |
| MIP-Cor | Off |
| MIP-Tra | Off |
| MIP-Time | Off |
| Save original images | On |

## Inline - Composing

| | |
|---|---|
| Inline Composing | Off |
| Distortion Corr. | Off |

## Sequence - Part 1

| | |
|---|---|
| Introduction | Off |
| Dimension | 2D |
| Asymmetric echo | Allowed |
| Flow comp. | No |
| Multi-slice mode | Interleaved |
| Echo spacing | 5 ms |
| Bandwidth | 580 Hz/Px |

## Sequence - Part 2

| | |
|---|---|
| RF pulse type | Low SAR |
| Gradient mode | Whisper |
| Excitation | Slice-sel. |
| RF spoiling | On |
| Turbo factor | 60 |

## Sequence - Assistant

| | |
|---|---|
| Mode | Off |

**\\Protocol Development\MJT\Neonate-development\PRENCOG_neonate\MTSatOn_neonate_v3**

TA: 2:36 PM: REF Voxel size: 1.6×1.6×1.6 mmPAT: 4 Rel. SNR: 1.00 : qfl

## Properties

| | |
|---|---|
| Prio recon | Off |
| Load images to viewer | On |
| Inline movie | Off |
| Auto store images | On |
| Load images to stamp segments | Off |
| Load images to graphic segments | Off |
| Auto open inline display | Off |
| Auto close inline display | Off |
| Start measurement without further preparation | Off |
| Wait for user to start | Off |
| Start measurements | Single measurement |

## Routine

| | |
|---|---|
| Slab group | 1 |
| Slabs | 1 |
| Dist. factor | 20 % |
| Position | R6.7 P19.4 H34.5 mm |
| Orientation | Sagittal |
| Phase enc. dir. | A >> P |
| AutoAlign | --- |
| Phase oversampling | 0 % |
| Slice oversampling | 0.0 % |
| Slices per slab | 88 |
| FoV read | 158 mm |
| FoV phase | 100.0 % |
| Slice thickness | 1.65 mm |
| TR | 75.0 ms |
| TE 1 | 2.21 ms |
| TE 2 | 6.31 ms |
| TE 3 | 10.41 ms |
| Averages | 1 |
| Concatenations | 1 |
| Filter | Prescan Normalize |
| Coil elements | PeH;PeN |

## Contrast - Common

| | |
|---|---|
| TR | 75.0 ms |
| TE 1 | 2.21 ms |
| TE 2 | 6.31 ms |
| TE 3 | 10.41 ms |
| MTC | On |
| Magn. preparation | None |
| Flip angle | 5 deg |
| Fat suppr. | None |
| Water suppr. | None |
| SWI | Off |

## Contrast - Dynamic

| | |
|---|---|
| Averages | 1 |
| Averaging mode | Short term |
| Reconstruction | Magnitude |
| Measurements | 1 |
| Multiple series | Each measurement |

## Resolution - Common

| | |
|---|---|
| FoV read | 158 mm |
| FoV phase | 100.0 % |
| Slice thickness | 1.65 mm |
| Base resolution | 96 |
| Phase resolution | 100 % |
| Slice resolution | 100 % |
| Phase partial Fourier | Off |
| Slice partial Fourier | Off |
| Interpolation | Off |

## Resolution - iPAT

| | |
|---|---|
| PAT mode | GRAPPA |
| Accel. factor PE | 2 |
| Ref. lines PE | 24 |
| Accel. factor 3D | 2 |
| Ref. lines 3D | 24 |
| Reference scan mode | Integrated |

## Resolution - Filter Image

| | |
|---|---|
| Image Filter | Off |
| Distortion Corr. | Off |
| Prescan Normalize | On |
| Unfiltered images | Off |
| Normalize | Off |
| B1 filter | Off |

## Resolution - Filter Rawdata

| | |
|---|---|
| Raw filter | Off |
| Elliptical filter | Off |

## Geometry - Common

| | |
|---|---|
| Slab group | 1 |
| Slabs | 1 |
| Dist. factor | 20 % |
| Position | R6.7 P19.4 H34.5 mm |
| Orientation | Sagittal |
| Phase enc. dir. | A >> P |
| Slice oversampling | 0.0 % |
| Slices per slab | 88 |
| FoV read | 158 mm |
| FoV phase | 100.0 % |
| Slice thickness | 1.65 mm |
| TR | 75.0 ms |
| Multi-slice mode | Interleaved |
| Series | Interleaved |
| Concatenations | 1 |

## Geometry - AutoAlign

| | |
|---|---|
| Slab group | 1 |
| Position | R6.7 P19.4 H34.5 mm |
| Orientation | Sagittal |
| Phase enc. dir. | A >> P |
| AutoAlign | --- |
| Initial Position | R6.7 P19.4 H34.5 |
| R | 6.7 mm |
| P | 19.4 mm |
| H | 34.5 mm |
| Initial Rotation | 0.00 deg |
| Initial Orientation | Sagittal |

## Geometry - Saturation

| | |
|---|---|
| Saturation mode | Standard |
| Fat suppr. | None |
| Water suppr. | None |

## Geometry - Saturation

| | |
|---|---|
| Special sat. | None |

## Geometry - Tim Planning Suite

| | |
|---|---|
| Set-n-Go Protocol | Off |
| Table position | H |
| Table position | 0 mm |
| Inline Composing | Off |

## System - Miscellaneous

| | |
|---|---|
| Positioning mode | REF |
| Table position | H |
| Table position | 0 mm |
| MSMA | S - C - T |
| Sagittal | R >> L |
| Coronal | A >> P |
| Transversal | F >> H |
| Coil Combine Mode | Sum of Squares |
| Save uncombined | Off |
| Matrix Optimization | Off |
| AutoAlign | --- |
| Coil Select Mode | Off - AutoCoilSelect |

## System - Adjustments

| | |
|---|---|
| B0 Shim mode | Standard |
| B1 Shim mode | TrueForm |
| Adjust with body coil | Off |
| Confirm freq. adjustment | Off |
| Assume Dominant Fat | Off |
| Assume Silicone | Off |
| Adjustment Tolerance | Auto |

## System - Adjust Volume

| | |
|---|---|
| Position | R6.7 P19.4 H34.5 mm |
| Orientation | Sagittal |
| Rotation | 0.00 deg |
| A >> P | 158 mm |
| F >> H | 158 mm |
| R >> L | 146 mm |
| Reset | Off |

## System - pTx Volumes

| | |
|---|---|
| B1 Shim mode | TrueForm |
| Excitation | Non-sel. |

## System - Tx/Rx

| | |
|---|---|
| Frequency 1H | 123.244475 MHz |
| Correction factor | 1 |
| Gain | Low |
| Img. Scale Cor. | 3.000 |
| Reset | Off |
| ? Ref. amplitude 1H | 0.000 V |

## Physio - Signal1

| | |
|---|---|
| 1st Signal/Mode | None |
| TR | 75.0 ms |
| Concatenations | 1 |
| Segments | 1 |

## Physio - Cardiac

| | |
|---|---|
| Tagging | None |
| Magn. preparation | None |
| Fat suppr. | None |
| Dark blood | Off |

## Physio - Cardiac

| | |
|---|---|
| FoV read | 158 mm |
| FoV phase | 100.0 % |
| Phase resolution | 100 % |

## Physio - PACE

| | |
|---|---|
| Resp. control | Off |
| Concatenations | 1 |

## Inline - Common

| | |
|---|---|
| Subtract | Off |
| Measurements | 1 |
| StdDev | Off |
| Liver registration | Off |
| Save original images | On |

## Inline - MIP

| | |
|---|---|
| MIP-Sag | Off |
| MIP-Cor | Off |
| MIP-Tra | Off |
| MIP-Time | Off |
| Save original images | On |

## Inline - Soft Tissue

| | |
|---|---|
| Wash - In | Off |
| Wash - Out | Off |
| TTP | Off |
| PEI | Off |
| MIP - time | Off |
| Measurements | 1 |

## Inline - Composing

| | |
|---|---|
| Inline Composing | Off |
| Distortion Corr. | Off |

## Inline - MapIt

| | |
|---|---|
| Save original images | On |
| MapIt | None |
| Flip angle | 5 deg |
| Measurements | 1 |
| Contrasts | 3 |
| TR | 75.0 ms |
| TE 1 | 2.21 ms |
| TE 2 | 6.31 ms |
| TE 3 | 10.41 ms |

## Sequence - Part 1

| | |
|---|---|
| Introduction | Off |
| Dimension | 3D |
| Elliptical scanning | On |
| Phase stabilisation | Off |
| Asymmetric echo | Off |
| Contrasts | 3 |
| Flow comp. 1 | No |
| Readout mode | Monopolar |
| Multi-slice mode | Interleaved |
| Bandwidth 1 | 350 Hz/Px |
| Bandwidth 2 | 350 Hz/Px |
| Bandwidth 3 | 350 Hz/Px |

## Sequence - Part 2

| | |
|---|---|
| Segments | 1 |
| Acoustic noise reduction | Active |
| RF pulse type | Low SAR |

## Sequence - Part 2

| | |
|---|---|
| Gradient mode | Normal |
| Excitation | Non-sel. |
| RF spoiling | On |

## Sequence - Assistant

| | |
|---|---|
| Mode | Off |
| Allowed delay | 30 s |

**\\Protocol Development\MJT\Neonate-development\PRENCOG_neonate\MTSatOff_neonate_v3**

TA: 2:36 PM: REF Voxel size: 1.6×1.6×1.6 mmPAT: 4 Rel. SNR: 1.00 : qfl

## Properties

| | |
|---|---|
| Prio recon | Off |
| Load images to viewer | On |
| Inline movie | Off |
| Auto store images | On |
| Load images to stamp segments | Off |
| Load images to graphic segments | Off |
| Auto open inline display | Off |
| Auto close inline display | Off |
| Start measurement without further preparation | Off |
| Wait for user to start | Off |
| Start measurements | Single measurement |

## Routine

| | |
|---|---|
| Slab group | 1 |
| Slabs | 1 |
| Dist. factor | 20 % |
| Position | R6.7 P19.4 H34.5 mm |
| Orientation | Sagittal |
| Phase enc. dir. | A >> P |
| AutoAlign | --- |
| Phase oversampling | 0 % |
| Slice oversampling | 0.0 % |
| Slices per slab | 88 |
| FoV read | 158 mm |
| FoV phase | 100.0 % |
| Slice thickness | 1.65 mm |
| TR | 75.0 ms |
| TE 1 | 2.21 ms |
| TE 2 | 6.31 ms |
| TE 3 | 10.41 ms |
| TE 4 | 15.00 ms |
| TE 5 | 20.00 ms |
| TE 6 | 25.00 ms |
| TE 7 | 30.00 ms |
| TE 8 | 35.00 ms |
| TE 9 | 40.00 ms |
| TE 10 | 45.00 ms |
| TE 11 | 50.00 ms |
| Averages | 1 |
| Concatenations | 1 |
| Filter | Prescan Normalize |
| Coil elements | PeH;PeN |

## Contrast - Common

| | |
|---|---|
| TR | 75.0 ms |
| TE 1 | 2.21 ms |
| TE 2 | 6.31 ms |
| TE 3 | 10.41 ms |
| TE 4 | 15.00 ms |
| TE 5 | 20.00 ms |
| TE 6 | 25.00 ms |
| TE 7 | 30.00 ms |
| TE 8 | 35.00 ms |
| TE 9 | 40.00 ms |
| TE 10 | 45.00 ms |
| TE 11 | 50.00 ms |
| MTC | Off |
| Magn. preparation | None |
| Flip angle | 5 deg |
| Fat suppr. | None |

## Contrast - Common

| | |
|---|---|
| Water suppr. | None |
| SWI | Off |

## Contrast - Dynamic

| | |
|---|---|
| Averages | 1 |
| Averaging mode | Short term |
| Reconstruction | Magn./Phase |
| Measurements | 1 |
| Multiple series | Each measurement |

## Resolution - Common

| | |
|---|---|
| FoV read | 158 mm |
| FoV phase | 100.0 % |
| Slice thickness | 1.65 mm |
| Base resolution | 96 |
| Phase resolution | 100 % |
| Slice resolution | 100 % |
| Phase partial Fourier | Off |
| Slice partial Fourier | Off |
| Interpolation | Off |

## Resolution - iPAT

| | |
|---|---|
| PAT mode | GRAPPA |
| Accel. factor PE | 2 |
| Ref. lines PE | 24 |
| Accel. factor 3D | 2 |
| Ref. lines 3D | 24 |
| Reference scan mode | Integrated |

## Resolution - Filter Image

| | |
|---|---|
| Image Filter | Off |
| Distortion Corr. | Off |
| Prescan Normalize | On |
| Unfiltered images | Off |
| Normalize | Off |
| B1 filter | Off |

## Resolution - Filter Rawdata

| | |
|---|---|
| Raw filter | Off |
| Elliptical filter | Off |

## Geometry - Common

| | |
|---|---|
| Slab group | 1 |
| Slabs | 1 |
| Dist. factor | 20 % |
| Position | R6.7 P19.4 H34.5 mm |
| Orientation | Sagittal |
| Phase enc. dir. | A >> P |
| Slice oversampling | 0.0 % |
| Slices per slab | 88 |
| FoV read | 158 mm |
| FoV phase | 100.0 % |
| Slice thickness | 1.65 mm |
| TR | 75.0 ms |
| Multi-slice mode | Interleaved |
| Series | Interleaved |
| Concatenations | 1 |

## Geometry - AutoAlign

| | |
|---|---|
| Slab group | 1 |

## Geometry - AutoAlign

| | |
|---|---|
| Position | R6.7 P19.4 H34.5 mm |
| Orientation | Sagittal |
| Phase enc. dir. | A >> P |
| AutoAlign | --- |
| Initial Position | R6.7 P19.4 H34.5 |
| R | 6.7 mm |
| P | 19.4 mm |
| H | 34.5 mm |
| Initial Rotation | 0.00 deg |
| Initial Orientation | Sagittal |

## Geometry - Saturation

| | |
|---|---|
| Saturation mode | Standard |
| Fat suppr. | None |
| Water suppr. | None |
| Special sat. | None |

## Geometry - Tim Planning Suite

| | |
|---|---|
| Set-n-Go Protocol | Off |
| Table position | H |
| Table position | 0 mm |
| Inline Composing | Off |

## System - Miscellaneous

| | |
|---|---|
| Positioning mode | REF |
| Table position | H |
| Table position | 0 mm |
| MSMA | S - C - T |
| Sagittal | R >> L |
| Coronal | A >> P |
| Transversal | F >> H |
| Coil Combine Mode | Sum of Squares |
| Save uncombined | Off |
| Matrix Optimization | Off |
| AutoAlign | --- |
| Coil Select Mode | Off - AutoCoilSelect |

## System - Adjustments

| | |
|---|---|
| B0 Shim mode | Standard |
| B1 Shim mode | TrueForm |
| Adjust with body coil | Off |
| Confirm freq. adjustment | Off |
| Assume Dominant Fat | Off |
| Assume Silicone | Off |
| Adjustment Tolerance | Auto |

## System - Adjust Volume

| | |
|---|---|
| Position | R6.7 P19.4 H34.5 mm |
| Orientation | Sagittal |
| Rotation | 0.00 deg |
| A >> P | 158 mm |
| F >> H | 158 mm |
| R >> L | 146 mm |
| Reset | Off |

## System - pTx Volumes

| | |
|---|---|
| B1 Shim mode | TrueForm |
| Excitation | Non-sel. |

## System - Tx/Rx

| | |
|---|---|
| Frequency 1H | 123.244475 MHz |
| Correction factor | 1 |
| Gain | Low |

## System - Tx/Rx

| | |
|---|---|
| Img. Scale Cor. | 3.000 |
| Reset | Off |
| ? Ref. amplitude 1H | 0.000 V |

## Physio - Signal1

| | |
|---|---|
| 1st Signal/Mode | None |
| TR | 75.0 ms |
| Concatenations | 1 |
| Segments | 1 |

## Physio - Cardiac

| | |
|---|---|
| Tagging | None |
| Magn. preparation | None |
| Fat suppr. | None |
| Dark blood | Off |
| FoV read | 158 mm |
| FoV phase | 100.0 % |
| Phase resolution | 100 % |

## Physio - PACE

| | |
|---|---|
| Resp. control | Off |
| Concatenations | 1 |

## Inline - Common

| | |
|---|---|
| Subtract | Off |
| Measurements | 1 |
| StdDev | Off |
| Liver registration | Off |
| Save original images | On |

## Inline - MIP

| | |
|---|---|
| MIP-Sag | Off |
| MIP-Cor | Off |
| MIP-Tra | Off |
| MIP-Time | Off |
| Save original images | On |

## Inline - Soft Tissue

| | |
|---|---|
| Wash - In | Off |
| Wash - Out | Off |
| TTP | Off |
| PEI | Off |
| MIP - time | Off |
| Measurements | 1 |

## Inline - Composing

| | |
|---|---|
| Inline Composing | Off |
| Distortion Corr. | Off |

## Inline - MapIt

| | |
|---|---|
| Noise threshold | 20 |
| Save original images | On |
| MapIt | T2* map |
| Flip angle | 5 deg |
| Measurements | 1 |
| Contrasts | 11 |
| TR | 75.0 ms |
| TE 1 | 2.21 ms |
| TE 2 | 6.31 ms |
| TE 3 | 10.41 ms |
| TE 4 | 15.00 ms |
| TE 5 | 20.00 ms |
| TE 6 | 25.00 ms |

## Inline - MapIt

| | |
|---|---|
| TE 7 | 30.00 ms |
| TE 8 | 35.00 ms |
| TE 9 | 40.00 ms |
| TE 10 | 45.00 ms |
| TE 11 | 50.00 ms |

## Sequence - Part 1

| | |
|---|---|
| Introduction | Off |
| Dimension | 3D |
| Elliptical scanning | On |
| Phase stabilisation | Off |
| Asymmetric echo | Off |
| Contrasts | 11 |
| Flow comp. 1 | No |
| Readout mode | Monopolar |
| Multi-slice mode | Interleaved |
| Bandwidth 1 | 350 Hz/Px |
| Bandwidth 2 | 350 Hz/Px |
| Bandwidth 3 | 350 Hz/Px |
| Bandwidth 4 | 350 Hz/Px |
| Bandwidth 5 | 350 Hz/Px |
| Bandwidth 6 | 350 Hz/Px |
| Bandwidth 7 | 350 Hz/Px |
| Bandwidth 8 | 350 Hz/Px |
| Bandwidth 9 | 350 Hz/Px |
| Bandwidth 10 | 350 Hz/Px |
| Bandwidth 11 | 350 Hz/Px |

## Sequence - Part 2

| | |
|---|---|
| Segments | 1 |
| Acoustic noise reduction | Active |
| RF pulse type | Low SAR |
| Gradient mode | Normal |
| Excitation | Non-sel. |
| RF spoiling | On |

## Sequence - Assistant

| | |
|---|---|
| Mode | Off |
| Allowed delay | 30 s |

| \\Protocol Development\MJT\Neonate-development\PRENCOG_neonate\MTSatT1_neonate_v3 |
|---|
| TA: 0:31 PM: REF Voxel size: 1.6×1.6×1.6 mmPAT: 4 Rel. SNR: 1.00 : qfl |

## Properties

| | |
|---|---|
| Prio recon | Off |
| Load images to viewer | On |
| Inline movie | Off |
| Auto store images | On |
| Load images to stamp segments | Off |
| Load images to graphic segments | Off |
| Auto open inline display | Off |
| Auto close inline display | Off |
| Start measurement without further preparation | Off |
| Wait for user to start | Off |
| Start measurements | Single measurement |

## Routine

| | |
|---|---|
| Slab group | 1 |
| Slabs | 1 |
| Dist. factor | 20 % |
| Position | R6.7 P19.4 H34.5 mm |
| Orientation | Sagittal |
| Phase enc. dir. | A >> P |
| AutoAlign | --- |
| Phase oversampling | 0 % |
| Slice oversampling | 0.0 % |
| Slices per slab | 88 |
| FoV read | 158 mm |
| FoV phase | 100.0 % |
| Slice thickness | 1.65 mm |
| TR | 15.0 ms |
| TE 1 | 2.21 ms |
| TE 2 | 6.31 ms |
| TE 3 | 10.41 ms |
| Averages | 1 |
| Concatenations | 1 |
| Filter | Prescan Normalize |
| Coil elements | PeH;PeN |

## Contrast - Common

| | |
|---|---|
| TR | 15.0 ms |
| TE 1 | 2.21 ms |
| TE 2 | 6.31 ms |
| TE 3 | 10.41 ms |
| MTC | Off |
| Magn. preparation | None |
| Flip angle | 14 deg |
| Fat suppr. | None |
| Water suppr. | None |
| SWI | Off |

## Contrast - Dynamic

| | |
|---|---|
| Averages | 1 |
| Averaging mode | Short term |
| Reconstruction | Magnitude |
| Measurements | 1 |
| Multiple series | Each measurement |

## Resolution - Common

| | |
|---|---|
| FoV read | 158 mm |
| FoV phase | 100.0 % |
| Slice thickness | 1.65 mm |
| Base resolution | 96 |

## Resolution - Common

| | |
|---|---|
| Phase resolution | 100 % |
| Slice resolution | 100 % |
| Phase partial Fourier | Off |
| Slice partial Fourier | Off |
| Interpolation | Off |

## Resolution - iPAT

| | |
|---|---|
| PAT mode | GRAPPA |
| Accel. factor PE | 2 |
| Ref. lines PE | 24 |
| Accel. factor 3D | 2 |
| Ref. lines 3D | 24 |
| Reference scan mode | Integrated |

## Resolution - Filter Image

| | |
|---|---|
| Image Filter | Off |
| Distortion Corr. | Off |
| Prescan Normalize | On |
| Unfiltered images | Off |
| Normalize | Off |
| B1 filter | Off |

## Resolution - Filter Rawdata

| | |
|---|---|
| Raw filter | Off |
| Elliptical filter | Off |

## Geometry - Common

| | |
|---|---|
| Slab group | 1 |
| Slabs | 1 |
| Dist. factor | 20 % |
| Position | R6.7 P19.4 H34.5 mm |
| Orientation | Sagittal |
| Phase enc. dir. | A >> P |
| Slice oversampling | 0.0 % |
| Slices per slab | 88 |
| FoV read | 158 mm |
| FoV phase | 100.0 % |
| Slice thickness | 1.65 mm |
| TR | 15.0 ms |
| Multi-slice mode | Interleaved |
| Series | Interleaved |
| Concatenations | 1 |

## Geometry - AutoAlign

| | |
|---|---|
| Slab group | 1 |
| Position | R6.7 P19.4 H34.5 mm |
| Orientation | Sagittal |
| Phase enc. dir. | A >> P |
| AutoAlign | --- |
| Initial Position | R6.7 P19.4 H34.5 |
| R | 6.7 mm |
| P | 19.4 mm |
| H | 34.5 mm |
| Initial Rotation | 0.00 deg |
| Initial Orientation | Sagittal |

## Geometry - Saturation

| | |
|---|---|
| Saturation mode | Standard |
| Fat suppr. | None |
| Water suppr. | None |

## Geometry - Saturation

| | |
|---|---|
| Special sat. | None |

## Geometry - Tim Planning Suite

| | |
|---|---|
| Set-n-Go Protocol | Off |
| Table position | H |
| Table position | 0 mm |
| Inline Composing | Off |

## System - Miscellaneous

| | |
|---|---|
| Positioning mode | REF |
| Table position | H |
| Table position | 0 mm |
| MSMA | S - C - T |
| Sagittal | R >> L |
| Coronal | A >> P |
| Transversal | F >> H |
| Coil Combine Mode | Sum of Squares |
| Save uncombined | Off |
| Matrix Optimization | Off |
| AutoAlign | --- |
| Coil Select Mode | Off - AutoCoilSelect |

## System - Adjustments

| | |
|---|---|
| B0 Shim mode | Standard |
| B1 Shim mode | TrueForm |
| Adjust with body coil | Off |
| Confirm freq. adjustment | Off |
| Assume Dominant Fat | Off |
| Assume Silicone | Off |
| Adjustment Tolerance | Auto |

## System - Adjust Volume

| | |
|---|---|
| Position | R6.7 P19.4 H34.5 mm |
| Orientation | Sagittal |
| Rotation | 0.00 deg |
| A >> P | 158 mm |
| F >> H | 158 mm |
| R >> L | 146 mm |
| Reset | Off |

## System - pTx Volumes

| | |
|---|---|
| B1 Shim mode | TrueForm |
| Excitation | Non-sel. |

## System - Tx/Rx

| | |
|---|---|
| Frequency 1H | 123.244475 MHz |
| Correction factor | 1 |
| Gain | Low |
| Img. Scale Cor. | 3.000 |
| Reset | Off |
| ? Ref. amplitude 1H | 0.000 V |

## Physio - Signal1

| | |
|---|---|
| 1st Signal/Mode | None |
| TR | 15.0 ms |
| Concatenations | 1 |
| Segments | 1 |

## Physio - Cardiac

| | |
|---|---|
| Tagging | None |
| Magn. preparation | None |
| Fat suppr. | None |
| Dark blood | Off |

## Physio - Cardiac

| | |
|---|---|
| FoV read | 158 mm |
| FoV phase | 100.0 % |
| Phase resolution | 100 % |

## Physio - PACE

| | |
|---|---|
| Resp. control | Off |
| Concatenations | 1 |

## Inline - Common

| | |
|---|---|
| Subtract | Off |
| Measurements | 1 |
| StdDev | Off |
| Liver registration | Off |
| Save original images | On |

## Inline - MIP

| | |
|---|---|
| MIP-Sag | Off |
| MIP-Cor | Off |
| MIP-Tra | Off |
| MIP-Time | Off |
| Save original images | On |

## Inline - Soft Tissue

| | |
|---|---|
| Wash - In | Off |
| Wash - Out | Off |
| TTP | Off |
| PEI | Off |
| MIP - time | Off |
| Measurements | 1 |

## Inline - Composing

| | |
|---|---|
| Inline Composing | Off |
| Distortion Corr. | Off |

## Inline - MapIt

| | |
|---|---|
| Save original images | On |
| MapIt | None |
| Flip angle | 14 deg |
| Measurements | 1 |
| Contrasts | 3 |
| TR | 15.0 ms |
| TE 1 | 2.21 ms |
| TE 2 | 6.31 ms |
| TE 3 | 10.41 ms |

## Sequence - Part 1

| | |
|---|---|
| Introduction | Off |
| Dimension | 3D |
| Elliptical scanning | On |
| Phase stabilisation | Off |
| Asymmetric echo | Off |
| Contrasts | 3 |
| Flow comp. 1 | No |
| Readout mode | Monopolar |
| Multi-slice mode | Interleaved |
| Bandwidth 1 | 350 Hz/Px |
| Bandwidth 2 | 350 Hz/Px |
| Bandwidth 3 | 350 Hz/Px |

## Sequence - Part 2

| | |
|---|---|
| Segments | 1 |
| Acoustic noise reduction | Active |
| RF pulse type | Low SAR |

## Sequence - Part 2

| | |
|---|---|
| Gradient mode | Normal |
| Excitation | Non-sel. |
| RF spoiling | On |

## Sequence - Assistant

| | |
|---|---|
| Mode | Off |
| Allowed delay | 30 s |

**\\Protocol Development\MJT\Neonate-development\PRENCOG_neonate\SWI_v2**

TA: 2:23 PM: FIX Voxel size: 0.8×0.8×3.0 mmPAT: 3 Rel. SNR: 1.00 : qswi_r

## Properties

| | |
|---|---|
| Prio recon | Off |
| Load images to viewer | On |
| Inline movie | Off |
| Auto store images | On |
| Load images to stamp segments | Off |
| Load images to graphic segments | Off |
| Auto open inline display | Off |
| Auto close inline display | Off |
| Start measurement without further preparation | Off |
| Wait for user to start | Off |
| Start measurements | Single measurement |

## Routine

| | |
|---|---|
| Slab group | 1 |
| Slabs | 1 |
| Dist. factor | 20 % |
| Position | L0.0 A2.3 H2.2 mm |
| Orientation | Transversal |
| Phase enc. dir. | R >> L |
| AutoAlign | --- |
| Phase oversampling | 0 % |
| Slice oversampling | 20.0 % |
| Slices per slab | 40 |
| FoV read | 240 mm |
| FoV phase | 84.4 % |
| Slice thickness | 3.00 mm |
| TR | 28.0 ms |
| TE | 20.00 ms |
| Averages | 1 |
| Concatenations | 1 |
| Filter | Prescan Normalize |
| Coil elements | HEA;HEP |

## Contrast - Common

| | |
|---|---|
| TR | 28.0 ms |
| TE | 20.00 ms |
| MTC | Off |
| Magn. preparation | None |
| Flip angle | 9 deg |
| Fat suppr. | None |
| Water suppr. | None |
| SWI | On |

## Contrast - Dynamic

| | |
|---|---|
| Averages | 1 |
| Averaging mode | Short term |
| Reconstruction | Magn./Phase |
| Measurements | 1 |
| Multiple series | Each measurement |

## Resolution - Common

| | |
|---|---|
| FoV read | 240 mm |
| FoV phase | 84.4 % |
| Slice thickness | 3.00 mm |
| Base resolution | 320 |
| Phase resolution | 100 % |
| Slice resolution | 100 % |
| Phase partial Fourier | Off |
| Slice partial Fourier | Off |

## Resolution - Common

| | |
|---|---|
| Interpolation | Off |

## Resolution - iPAT

| | |
|---|---|
| PAT mode | GRAPPA |
| Accel. factor PE | 3 |
| Ref. lines PE | 24 |
| Accel. factor 3D | 1 |
| Reference scan mode | Integrated |

## Resolution - Filter Image

| | |
|---|---|
| Image Filter | Off |
| Distortion Corr. | Off |
| Prescan Normalize | On |
| Unfiltered images | Off |
| Normalize | Off |
| B1 filter | Off |

## Resolution - Filter Rawdata

| | |
|---|---|
| Raw filter | Off |
| Elliptical filter | Off |

## Geometry - Common

| | |
|---|---|
| Slab group | 1 |
| Slabs | 1 |
| Dist. factor | 20 % |
| Position | L0.0 A2.3 H2.2 mm |
| Orientation | Transversal |
| Phase enc. dir. | R >> L |
| Slice oversampling | 20.0 % |
| Slices per slab | 40 |
| FoV read | 240 mm |
| FoV phase | 84.4 % |
| Slice thickness | 3.00 mm |
| TR | 28.0 ms |
| Multi-slice mode | Interleaved |
| Series | Interleaved |
| Concatenations | 1 |

## Geometry - AutoAlign

| | |
|---|---|
| Slab group | 1 |
| Position | L0.0 A2.3 H2.2 mm |
| Orientation | Transversal |
| Phase enc. dir. | R >> L |
| AutoAlign | --- |
| Initial Position | L0.0 A2.3 H2.2 |
| L | 0.0 mm |
| A | 2.3 mm |
| H | 2.2 mm |
| Initial Rotation | 89.61 deg |
| Initial Orientation | Transversal |

## Geometry - Saturation

| | |
|---|---|
| Saturation mode | Standard |
| Fat suppr. | None |
| Water suppr. | None |
| Special sat. | None |

## Geometry - Tim Planning Suite

| | |
|---|---|
| Set-n-Go Protocol | Off |
| Table position | H |

## Geometry - Tim Planning Suite

| | |
|---|---|
| Table position | 0 mm |
| Inline Composing | Off |

## System - Miscellaneous

| | |
|---|---|
| Positioning mode | FIX |
| Table position | H |
| Table position | 0 mm |
| MSMA | S - C - T |
| Sagittal | R >> L |
| Coronal | A >> P |
| Transversal | F >> H |
| Coil Combine Mode | Adaptive Combine |
| Save uncombined | Off |
| Matrix Optimization | Off |
| AutoAlign | --- |
| Coil Select Mode | On - AutoCoilSelect |

## System - Adjustments

| | |
|---|---|
| B0 Shim mode | Standard |
| B1 Shim mode | TrueForm |
| Adjust with body coil | Off |
| Confirm freq. adjustment | Off |
| Assume Dominant Fat | Off |
| Assume Silicone | Off |
| Adjustment Tolerance | Auto |

## System - Adjust Volume

| | |
|---|---|
| Position | L0.0 A2.3 H2.2 mm |
| Orientation | Transversal |
| Rotation | 89.61 deg |
| R >> L | 203 mm |
| A >> P | 240 mm |
| F >> H | 120 mm |
| Reset | Off |

## System - pTx Volumes

| | |
|---|---|
| B1 Shim mode | TrueForm |
| Excitation | Slab-sel. |

## System - Tx/Rx

| | |
|---|---|
| Frequency 1H | 123.244475 MHz |
| Correction factor | 1 |
| Gain | Low |
| Img. Scale Cor. | 3.000 |
| Reset | Off |
| ? Ref. amplitude 1H | 0.000 V |

## Physio - Signal1

| | |
|---|---|
| 1st Signal/Mode | None |
| TR | 28.0 ms |
| Concatenations | 1 |
| Segments | 1 |

## Physio - Cardiac

| | |
|---|---|
| Tagging | None |
| Magn. preparation | None |
| Fat suppr. | None |
| Dark blood | Off |
| FoV read | 240 mm |
| FoV phase | 84.4 % |
| Phase resolution | 100 % |

## Physio - PACE

| | |
|---|---|
| Resp. control | Off |
| Concatenations | 1 |

## Inline - Common

| | |
|---|---|
| Subtract | Off |
| Measurements | 1 |
| StdDev | Off |
| Liver registration | Off |
| Save original images | On |

## Inline - MIP

| | |
|---|---|
| MIP-Sag | Off |
| MIP-Cor | Off |
| MIP-Tra | Off |
| MIP-Time | Off |
| Save original images | On |

## Inline - Soft Tissue

| | |
|---|---|
| Wash - In | Off |
| Wash - Out | Off |
| TTP | Off |
| PEI | Off |
| MIP - time | Off |
| Measurements | 1 |

## Inline - Composing

| | |
|---|---|
| Inline Composing | Off |
| Distortion Corr. | Off |

## Inline - MapIt

| | |
|---|---|
| Save original images | On |
| MapIt | None |
| Flip angle | 9 deg |
| Measurements | 1 |
| Contrasts | 1 |
| TR | 28.0 ms |
| TE | 20.00 ms |

## Sequence - Part 1

| | |
|---|---|
| Introduction | On |
| Dimension | 3D |
| Elliptical scanning | Off |
| Phase stabilisation | Off |
| Asymmetric echo | Off |
| Contrasts | 1 |
| Flow comp. | Yes |
| Multi-slice mode | Interleaved |
| Bandwidth | 120 Hz/Px |

## Sequence - Part 2

| | |
|---|---|
| Segments | 1 |
| Acoustic noise reduction | Active |
| RF pulse type | Fast |
| Gradient mode | Whisper |
| Excitation | Slab-sel. |
| RF spoiling | On |

## Sequence - Assistant

| | |
|---|---|
| Mode | Off |
| Allowed delay | 30 s |

**\\Protocol Development\MJT\Neonate-development\PRENCOG_neonate\t2_blade_dark-fluid_tra_v3**

TA: 3:22 PM: REF Voxel size: 0.9×0.9×3.0 mmPAT: 2 Rel. SNR: 1.00 : qtirB_rr

## Properties

| | |
|---|---|
| Prio recon | Off |
| Load images to viewer | On |
| Inline movie | Off |
| Auto store images | On |
| Load images to stamp segments | On |
| Load images to graphic segments | Off |
| Auto open inline display | Off |
| Auto close inline display | Off |
| Start measurement without further preparation | Off |
| Wait for user to start | Off |
| Start measurements | Single measurement |

## Routine

| | |
|---|---|
| Slice group | 1 |
| Slices | 40 |
| Dist. factor | 0 % |
| Position | Isocenter |
| Orientation | Transversal |
| Phase enc. dir. | R >> L |
| AutoAlign | --- |
| Phase oversampling | 0.0 % |
| FoV read | 240 mm |
| FoV phase | 100.0 % |
| Slice thickness | 3.0 mm |
| TR | 10000.0 ms |
| TE | 130 ms |
| Averages | 1 |
| Concatenations | 2 |
| Filter | Prescan Normalize |
| Coil elements | HEA;HEP |

## Contrast - Common

| | |
|---|---|
| TR | 10000.0 ms |
| TE | 130 ms |
| TD | 0.0 ms |
| MTC | Off |
| Magn. preparation | Slice-sel. IR |
| TI | 2606 ms |
| Flip angle | 130 deg |
| Fat suppr. | Fat sat. |
| Fat sat. mode | Strong |
| Water suppr. | None |
| Restore magn. | Off |
| Freeze suppressed tissue | On |

## Contrast - Dynamic

| | |
|---|---|
| Averages | 1 |
| Averaging mode | Short term |
| Reconstruction | Magnitude |
| Measurements | 1 |
| Multiple series | Each measurement |

## Resolution - Common

| | |
|---|---|
| FoV read | 240 mm |
| FoV phase | 100.0 % |
| Slice thickness | 3.0 mm |
| Base resolution | 256 |
| BLADE coverage | 100.0 % |
| Trajectory | BLADE |

## Resolution - Common

| | |
|---|---|
| Interpolation | Off |

## Resolution - iPAT

| | |
|---|---|
| PAT mode | GRAPPA |
| Accel. factor PE | 2 |
| Ref. lines PE | 8 |
| Reference scan mode | Integrated |

## Resolution - Filter Image

| | |
|---|---|
| Image Filter | Off |
| Distortion Corr. | Off |
| Prescan Normalize | On |
| Unfiltered images | Off |
| Normalize | Off |
| B1 filter | Off |

## Resolution - Filter Rawdata

| | |
|---|---|
| Raw filter | Off |
| Elliptical filter | Off |

## Geometry - Common

| | |
|---|---|
| Slice group | 1 |
| Slices | 40 |
| Dist. factor | 0 % |
| Position | Isocenter |
| Orientation | Transversal |
| Phase enc. dir. | R >> L |
| FoV read | 240 mm |
| FoV phase | 100.0 % |
| Slice thickness | 3.0 mm |
| TR | 10000.0 ms |
| Multi-slice mode | Interleaved |
| Series | Interleaved |
| Concatenations | 2 |

## Geometry - AutoAlign

| | |
|---|---|
| Slice group | 1 |
| Position | Isocenter |
| Orientation | Transversal |
| Phase enc. dir. | R >> L |
| AutoAlign | --- |
| Initial Position | Isocenter |
| L | 0.0 mm |
| P | 0.0 mm |
| H | 0.0 mm |
| Initial Rotation | 90.00 deg |
| Initial Orientation | Transversal |

## Geometry - Saturation

| | |
|---|---|
| Fat suppr. | Fat sat. |
| Fat sat. mode | Strong |
| Water suppr. | None |
| Restore magn. | Off |
| Special sat. | Parallel F |
| Gap | 10 mm |
| Thickness | 70 mm |

## Geometry - Navigator

## Geometry - Tim Planning Suite

| | |
|---|---|
| Set-n-Go Protocol | Off |
| Table position | H |
| Table position | 0 mm |
| Inline Composing | Off |

## System - Miscellaneous

| | |
|---|---|
| Positioning mode | REF |
| Table position | H |
| Table position | 0 mm |
| MSMA | S - C - T |
| Sagittal | R >> L |
| Coronal | A >> P |
| Transversal | F >> H |
| Coil Combine Mode | Adaptive Combine |
| Save uncombined | Off |
| Matrix Optimization | Off |
| AutoAlign | --- |
| Coil Select Mode | On - AutoCoilSelect |

## System - Adjustments

| | |
|---|---|
| B0 Shim mode | Standard |
| B1 Shim mode | TrueForm |
| Adjust with body coil | Off |
| Confirm freq. adjustment | Off |
| Assume Dominant Fat | Off |
| Assume Silicone | Off |
| Adjustment Tolerance | Auto |

## System - Adjust Volume

| | |
|---|---|
| Position | Isocenter |
| Orientation | Transversal |
| Rotation | 90.00 deg |
| R >> L | 240 mm |
| A >> P | 240 mm |
| F >> H | 120 mm |
| Reset | Off |

## System - pTx Volumes

| | |
|---|---|
| B1 Shim mode | TrueForm |

## System - Tx/Rx

| | |
|---|---|
| Frequency 1H | 123.244475 MHz |
| Correction factor | 1 |
| Gain | High |
| Img. Scale Cor. | 2.000 |
| Reset | Off |
| ? Ref. amplitude 1H | 0.000 V |

## Physio - Signal1

| | |
|---|---|
| 1st Signal/Mode | None |
| TR | 10000.0 ms |
| Concatenations | 2 |

## Physio - Cardiac

| | |
|---|---|
| Magn. preparation | Slice-sel. IR |
| TI | 2606 ms |
| Fat suppr. | Fat sat. |
| Dark blood | Off |
| FoV read | 240 mm |
| FoV phase | 100.0 % |
| BLADE coverage | 100.0 % |
| Trajectory | BLADE |

## Physio - PACE

| | |
|---|---|
| Resp. control | Off |
| Concatenations | 2 |

## Inline - Common

| | |
|---|---|
| Subtract | Off |
| Measurements | 1 |
| StdDev | Off |
| Save original images | On |

## Inline - MIP

| | |
|---|---|
| MIP-Sag | Off |
| MIP-Cor | Off |
| MIP-Tra | Off |
| MIP-Time | Off |
| Save original images | On |

## Inline - Composing

| | |
|---|---|
| Inline Composing | Off |
| Distortion Corr. | Off |

## Sequence - Part 1

| | |
|---|---|
| Introduction | On |
| Dimension | 2D |
| Compensate T2 decay | Off |
| Contrasts | 1 |
| Flow comp. | Read |
| Multi-slice mode | Interleaved |
| Free echo spacing | Off |
| Echo spacing | 8.64 ms |
| Bandwidth | 362 Hz/Px |

## Sequence - Part 2

| | |
|---|---|
| Define | Turbo factor |
| Echo trains per slice | 9 |
| Phase correction | Automatic |
| Acoustic noise reduction | Active |
| RF pulse type | Low SAR |
| Gradient mode | Normal |
| Hyperecho | Off |
| WARP | Off |
| Motion correction | On |
| Red. EC sensitivity | Off |
| Turbo factor | 28 |

## Sequence - Assistant

| | |
|---|---|
| Mode | Min flip angle |
| Min flip angle | 130 deg |
| Allowed delay | 30 s |
