# Supplementary material for: Preterm birth as a determinant of neurodevelopment and cognition in children (PRENCOG): protocol for an exposure-based cohort study in the UK
Source: BMJ Open. 2024 Sep 16;14(9):e085365. doi: 10.1136/bmjopen-2024-085365 (PMC11409314; doi:10.1136/bmjopen-2024-085365)
Supplement: online supplemental file 2 [file bmjopen-14-9-s002.pdf]

Table of contents

\\Study Protocols
BRAIN
Other
TEBC_5 year old - E161723
AAHead_Scout_32ch-head-coil
t1_mprage_sag
t2_space_sag
t2_blade_dark-fluid_tra
DTI_rev_PA
DTI_AP
MTSatOn_5y
MTSatOff_5y
MTSatT1w_5y
gre_field_mapping_3mm
ep2d_p2_s3_AP_pixar-1
ep2d_p2_s3_AP_pixar-2
ep2d_p2_s3_AP_sesame

\\Study Protocols\BRAIN\Other\TEBC_5 year old - E161723\AAHead_Scout_32ch-head-coil
TA: 0:14 PM: REF Voxel size: 1.6×1.6×1.6 mmPAT: 3 Rel. SNR: 1.00 : fl

Properties

Prio recon	Off
Load images to viewer	On
Inline movie	Off
Auto store images	On
Load images to stamp segments	On
Load images to graphic segments	On
Auto open inline display	Off
Auto close inline display	Off
Start measurement without further preparation	On
Wait for user to start	Off
Start measurements	Single measurement

Routine

Slab group	1
Slabs	1
Dist. factor	20 %
Position	Isocenter
Orientation	Sagittal
Phase enc. dir.	A >> P
Phase oversampling	0 %
Slice oversampling	0.0 %
Slices per slab	128
FoV read	260 mm
FoV phase	100.0 %
Slice thickness	1.6 mm
TR	3.15 ms
TE	1.37 ms
Averages	1
Concatenations	1
Filter	Prescan Normalize
Coil elements	HEA;HEP

Contrast - Common

TR	3.15 ms
TE	1.37 ms
Flip angle	8 deg

Contrast - Dynamic

Averages	1
Averaging mode	Short term
Reconstruction	Magnitude
Measurements	1

Resolution - Common

FoV read	260 mm
FoV phase	100.0 %
Slice thickness	1.6 mm
Base resolution	160
Phase resolution	100 %
Slice resolution	69 %
Phase partial Fourier	6/8
Slice partial Fourier	6/8
Trajectory	Cartesian

Resolution - iPAT

PAT mode	GRAPPA
Accel. factor PE	3
Ref. lines PE	24
Accel. factor 3D	1

Resolution - iPAT

Reference scan mode	Integrated

Resolution - Filter Image

Image Filter	Off
Distortion Corr.	Off
Prescan Normalize	On
Unfiltered images	Off
Normalize	Off
B1 filter	Off

Resolution - Filter Rawdata

Raw filter	Off
Elliptical filter	Off

Geometry - Common

Slab group	1
Slabs	1
Dist. factor	20 %
Position	Isocenter
Orientation	Sagittal
Phase enc. dir.	A >> P
Slice oversampling	0.0 %
Slices per slab	128
FoV read	260 mm
FoV phase	100.0 %
Slice thickness	1.6 mm
TR	3.15 ms
Multi-slice mode	Sequential
Series	Ascending
Concatenations	1

Geometry - AutoAlign

Slab group	1
Position	Isocenter
Orientation	Sagittal
Phase enc. dir.	A >> P
Initial Position	Isocenter
L	0.0 mm
P	0.0 mm
H	0.0 mm
Initial Rotation	0.00 deg
Initial Orientation	Transversal

Geometry - Tim Planning Suite

Set-n-Go Protocol	Off
Table position	H
Table position	0 mm
Inline Composing	Off

System - Miscellaneous

Positioning mode	REF
Table position	H
Table position	0 mm
MSMA	S - C - T
Sagittal	R >> L
Coronal	A >> P
Transversal	F >> H
Coil Combine Mode	Adaptive Combine
Save uncombined	Off
Matrix Optimization	Off

System - Miscellaneous

Coil Select Mode	Default

System - Adjustments

B0 Shim mode	Tune up
B1 Shim mode	TrueForm
Adjust with body coil	Off
Confirm freq. adjustment	Off
Assume Dominant Fat	Off
Assume Silicone	Off
Adjustment Tolerance	Auto

System - Adjust Volume

Position	Isocenter
Orientation	Transversal
Rotation	0.00 deg
A >> P	263 mm
R >> L	350 mm
F >> H	350 mm
Reset	Off

System - pTx Volumes

B1 Shim mode	TrueForm
Excitation	Non-sel.

System - Tx/Rx

Frequency 1H	123.244475 MHz
Correction factor	1
Gain	Low
Img. Scale Cor.	1.000
Reset	Off
? Ref. amplitude 1H	0.000 V

Physio - PACE

Resp. control	Off
Concatenations	1

Inline - Common

Flip angle	8 deg
Measurements	1
Time to center	6.2 s

Inline - Inline

Subtract	Off
Measurements	1
StdDev	Off
Save original images	On

Inline - MIP

MIP-Sag	Off
MIP-Cor	Off
MIP-Tra	Off
MIP-Time	Off
Save original images	On

Inline - Composing

Inline Composing	Off
Distortion Corr.	Off

Inline - MapIt

Save original images	On
MapIt	None
Flip angle	8 deg
Measurements	1
Contrasts	1
TR	3.15 ms
TE	1.37 ms

Sequence - Part 1

Introduction	On
Dimension	3D
Asymmetric echo	Weak
Contrasts	1
Multi-slice mode	Sequential
Bandwidth	540 Hz/Px

Sequence - Part 2

RF pulse type	Fast
Gradient mode	Normal
Excitation	Non-sel.
RF spoiling	On

Sequence - Assistant

Mode	Off

\\Study Protocols\BRAIN\Other\TEBC_5 year old - E161723\t1_mprage_sag
TA: 3:45 PM: FIX Voxel size: 1.0×1.0×1.0 mmPAT: 3 Rel. SNR: 1.00 : tfl

Properties

Prio recon	Off
Load images to viewer	On
Inline movie	Off
Auto store images	On
Load images to stamp segments	On
Load images to graphic segments	Off
Auto open inline display	Off
Auto close inline display	Off
Start measurement without further preparation	Off
Wait for user to start	Off
Start measurements	Single measurement

Routine

Slab group	1
Slabs	1
Dist. factor	50 %
Position	Isocenter
Orientation	Sagittal
Phase enc. dir.	A >> P
AutoAlign	Head > Basis
Phase oversampling	0 %
Slice oversampling	0.0 %
Slices per slab	192
FoV read	256 mm
FoV phase	100.0 %
Slice thickness	1.00 mm
TR	2500.0 ms
TE	4.69 ms
Averages	1
Concatenations	1
Filter	Prescan Normalize
Coil elements	HEA;HEP

Contrast - Common

TR	2500.0 ms
TE	4.69 ms
Magn. preparation	Non-sel. IR
TI	1180 ms
Flip angle	7 deg
Fat suppr.	Water excit. fast
Water suppr.	None

Contrast - Dynamic

Averages	1
Averaging mode	Long term
Reconstruction	Magnitude
Measurements	1
Multiple series	Each measurement

Resolution - Common

FoV read	256 mm
FoV phase	100.0 %
Slice thickness	1.00 mm
Base resolution	256
Phase resolution	100 %
Slice resolution	100 %
Phase partial Fourier	7/8
Slice partial Fourier	Off
Interpolation	Off

Resolution - iPAT

PAT mode	GRAPPA
Accel. factor PE	3
Ref. lines PE	24
Accel. factor 3D	1
Reference scan mode	Integrated

Resolution - Filter Image

Image Filter	Off
Distortion Corr.	Off
Prescan Normalize	On
Unfiltered images	Off
Normalize	Off
B1 filter	Off

Resolution - Filter Rawdata

Raw filter	Off
Elliptical filter	Off

Geometry - Common

Slab group	1
Slabs	1
Dist. factor	50 %
Position	Isocenter
Orientation	Sagittal
Phase enc. dir.	A >> P
Slice oversampling	0.0 %
Slices per slab	192
FoV read	256 mm
FoV phase	100.0 %
Slice thickness	1.00 mm
TR	2500.0 ms
Multi-slice mode	Single shot
Series	Interleaved
Concatenations	1

Geometry - AutoAlign

Slab group	1
Position	Isocenter
Orientation	Sagittal
Phase enc. dir.	A >> P
AutoAlign	Head > Basis
Initial Position	Isocenter
L	0.0 mm
P	0.0 mm
H	0.0 mm
Initial Rotation	0.00 deg
Initial Orientation	Sagittal

Geometry - Navigator

Geometry - Tim Planning Suite

Set-n-Go Protocol	Off
Table position	H
Table position	0 mm
Inline Composing	Off

System - Miscellaneous

Positioning mode	FIX
Table position	H
Table position	0 mm

System - Miscellaneous

MSMA	S - C - T
Sagittal	R >> L
Coronal	A >> P
Transversal	F >> H
Coil Combine Mode	Adaptive Combine
Save uncombined	Off
Matrix Optimization	Off
AutoAlign	Head > Basis
Coil Select Mode	On - AutoCoilSelect

System - Adjustments

B0 Shim mode	Standard
B1 Shim mode	TrueForm
Adjust with body coil	Off
Confirm freq. adjustment	Off
Assume Dominant Fat	Off
Assume Silicone	Off
Adjustment Tolerance	Auto

System - Adjust Volume

Position	Isocenter
Orientation	Sagittal
Rotation	0.00 deg
A >> P	256 mm
F >> H	256 mm
R >> L	192 mm
Reset	Off

System - pTx Volumes

B1 Shim mode	TrueForm
Excitation	Non-sel.

System - Tx/Rx

Frequency 1H	123.244475 MHz
Correction factor	1
Gain	Low
Img. Scale Cor.	2.000
Reset	Off
? Ref. amplitude 1H	0.000 V

Physio - Signal1

1st Signal/Mode	None
TR	2500.0 ms
Concatenations	1

Physio - Cardiac

Magn. preparation	Non-sel. IR
TI	1180 ms
Fat suppr.	Water excit. fast
Dark blood	Off
FoV read	256 mm
FoV phase	100.0 %
Phase resolution	100 %

Physio - PACE

Resp. control	Off
Concatenations	1

Inline - Common

Subtract	Off
Measurements	1
StdDev	Off
Save original images	On

Inline - MIP

MIP-Sag	Off
MIP-Cor	Off
MIP-Tra	Off
MIP-Time	Off
Save original images	On

Inline - Composing

Inline Composing	Off
Distortion Corr.	Off

Inline - MapIt

Save original images	On
MapIt	None
Flip angle	7 deg
Measurements	1
TR	2500.0 ms
TE	4.69 ms

Sequence - Part 1

Introduction	Off
Dimension	3D
Elliptical scanning	Off
Reordering	Linear
Asymmetric echo	Off
Flow comp.	No
Multi-slice mode	Single shot
Echo spacing	12 ms
Bandwidth	140 Hz/Px

Sequence - Part 2

RF pulse type	Fast
Gradient mode	Whisper
Excitation	Non-sel.
RF spoiling	On
Incr. Gradient spoiling	Off
Turbo factor	192

Sequence - Assistant

Mode	Off

\\Study Protocols\BRAIN\Other\TEBC_5 year old - E161723\t2_space_sag
TA: 3:30 PM: FIX Voxel size: 0.9×0.9×0.9 mmPAT: 4 Rel. SNR: 1.00 : spcR

Properties

Prio recon	Off
Load images to viewer	On
Inline movie	Off
Auto store images	On
Load images to stamp segments	Off
Load images to graphic segments	Off
Auto open inline display	Off
Auto close inline display	Off
Start measurement without further preparation	Off
Wait for user to start	Off
Start measurements	Single measurement

Routine

Slab group	1
Slabs	1
Position	Isocenter
Orientation	Sagittal
Phase enc. dir.	A >> P
AutoAlign	Head > Basis
Phase oversampling	0 %
Slice oversampling	0.0 %
Slices per slab	192
FoV read	230 mm
FoV phase	100.0 %
Slice thickness	0.90 mm
TR	3200 ms
TE	407 ms
Averages	1.4
Concatenations	1
Filter	Raw filter, Prescan Normalize
Coil elements	HEA;HEP

Contrast - Common

TR	3200 ms
TE	407 ms
MTC	Off
Magn. preparation	None
Fat suppr.	None
Blood suppr.	Off
Restore magn.	On

Contrast - Dynamic

Averages	1.4
Reconstruction	Magnitude
Measurements	1
Multiple series	Each measurement

Resolution - Common

FoV read	230 mm
FoV phase	100.0 %
Slice thickness	0.90 mm
Base resolution	256
Phase resolution	100 %
Slice resolution	100 %
Phase partial Fourier	Allowed
Slice partial Fourier	Off
Interpolation	Off

Resolution - iPAT

PAT mode	GRAPPA
Accel. factor PE	2
Ref. lines PE	24
Accel. factor 3D	2
Ref. lines 3D	24
Reference scan mode	Integrated

Resolution - Filter Image

Image Filter	Off
Distortion Corr.	Off
Prescan Normalize	On
Unfiltered images	Off
Normalize	Off
B1 filter	Off

Resolution - Filter Rawdata

Raw filter	On
Elliptical filter	Off

Geometry - Common

Slab group	1
Slabs	1
Position	Isocenter
Orientation	Sagittal
Phase enc. dir.	A >> P
Slice oversampling	0.0 %
Slices per slab	192
FoV read	230 mm
FoV phase	100.0 %
Slice thickness	0.90 mm
TR	3200 ms
Series	Interleaved
Concatenations	1

Geometry - AutoAlign

Slab group	1
Position	Isocenter
Orientation	Sagittal
Phase enc. dir.	A >> P
AutoAlign	Head > Basis
Initial Position	Isocenter
L	0.0 mm
P	0.0 mm
H	0.0 mm
Initial Rotation	0.00 deg
Initial Orientation	Sagittal

Geometry - Saturation

Fat suppr.	None
Restore magn.	On
Special sat.	None

Geometry - Navigator

Geometry - Tim Planning Suite

Set-n-Go Protocol	Off
Table position	H
Table position	0 mm
Inline Composing	Off

System - Miscellaneous

Positioning mode	FIX
Table position	H
Table position	0 mm
MSMA	S - C - T
Sagittal	R >> L
Coronal	A >> P
Transversal	F >> H
Coil Combine Mode	Adaptive Combine
Save uncombined	Off
Matrix Optimization	Off
AutoAlign	Head > Basis
Coil Select Mode	Off - AutoCoilSelect

System - Adjustments

B0 Shim mode	Tune up
B1 Shim mode	TrueForm
Adjust with body coil	Off
Confirm freq. adjustment	Off
Assume Dominant Fat	Off
Assume Silicone	Off
Adjustment Tolerance	Auto

System - Adjust Volume

Position	Isocenter
Orientation	Transversal
Rotation	0.00 deg
A >> P	263 mm
R >> L	350 mm
F >> H	350 mm
Reset	Off

System - pTx Volumes

B1 Shim mode	TrueForm
Excitation	Non-sel.

System - Tx/Rx

Frequency 1H	123.244475 MHz
Correction factor	1
Gain	High
Img. Scale Cor.	2.000
Reset	Off
? Ref. amplitude 1H	0.000 V

Physio - Signal1

1st Signal/Mode	None
Trigger delay	0 ms
TR	3200 ms
Concatenations	1

Physio - Cardiac

Magn. preparation	None
Fat suppr.	None
Dark blood	Off
FoV read	230 mm
FoV phase	100.0 %
Phase resolution	100 %

Physio - PACE

Resp. control	Off
Concatenations	1

Inline - Common

Subtract	Off

Inline - Common

Measurements	1
StdDev	Off
Save original images	On

Inline - MIP

MIP-Sag	Off
MIP-Cor	Off
MIP-Tra	Off
MIP-Time	Off
Save original images	On

Inline - Composing

Inline Composing	Off
Distortion Corr.	Off

Sequence - Part 1

Introduction	On
Dimension	3D
Elliptical scanning	Off
Reordering	Linear
Flow comp.	No
Echo spacing	4.52 ms
Adiabatic-mode	Off
Bandwidth	592 Hz/Px

Sequence - Part 2

Echo train duration	1049 ms
RF pulse type	Low SAR
Gradient mode	Whisper
Excitation	Non-sel.
Flip angle mode	T2 var
Turbo factor	282

Sequence - Assistant

Allowed delay	30 s

\\Study Protocols\BRAIN\Other\TEBC_5 year old - E161723\t2_blade_dark-fluid_tra

TA: 3:12 PM: FIX Voxel size: 0.9×0.9×3.0 mmPAT: 2 Rel. SNR: 1.00 : tirB_rr

Properties

Prio recon	Off
Load images to viewer	On
Inline movie	Off
Auto store images	On
Load images to stamp segments	On
Load images to graphic segments	Off
Auto open inline display	Off
Auto close inline display	Off
Start measurement without further preparation	Off
Wait for user to start	Off
Start measurements	Single measurement

Routine

Slice group	1
Slices	48
Dist. factor	0 %
Position	Isocenter
Orientation	Transversal
Phase enc. dir.	R >> L
AutoAlign	Head > Brain
Phase oversampling	0.0 %
FoV read	240 mm
FoV phase	100.0 %
Slice thickness	3.0 mm
TR	9500.0 ms
TE	124 ms
Averages	1
Concatenations	2
Filter	Prescan Normalize
Coil elements	HEA;HEP

Contrast - Common

TR	9500.0 ms
TE	124 ms
TD	0.0 ms
MTC	Off
Magn. preparation	Slice-sel. IR
TI	2556 ms
Flip angle	130 deg
Fat suppr.	Fat sat.
Fat sat. mode	Strong
Water suppr.	None
Restore magn.	Off
Freeze suppressed tissue	On

Contrast - Dynamic

Averages	1
Averaging mode	Short term
Reconstruction	Magnitude
Measurements	1
Multiple series	Each measurement

Resolution - Common

FoV read	240 mm
FoV phase	100.0 %
Slice thickness	3.0 mm
Base resolution	256
BLADE coverage	100.0 %
Trajectory	BLADE

Resolution - Common

Interpolation	Off

Resolution - iPAT

PAT mode	GRAPPA
Accel. factor PE	2
Ref. lines PE	8
Reference scan mode	Integrated

Resolution - Filter Image

Image Filter	Off
Distortion Corr.	Off
Prescan Normalize	On
Unfiltered images	Off
Normalize	Off
B1 filter	Off

Resolution - Filter Rawdata

Raw filter	Off
Elliptical filter	Off

Geometry - Common

Slice group	1
Slices	48
Dist. factor	0 %
Position	Isocenter
Orientation	Transversal
Phase enc. dir.	R >> L
FoV read	240 mm
FoV phase	100.0 %
Slice thickness	3.0 mm
TR	9500.0 ms
Multi-slice mode	Interleaved
Series	Interleaved
Concatenations	2

Geometry - AutoAlign

Slice group	1
Position	Isocenter
Orientation	Transversal
Phase enc. dir.	R >> L
AutoAlign	Head > Brain
Initial Position	Isocenter
L	0.0 mm
P	0.0 mm
H	0.0 mm
Initial Rotation	90.00 deg
Initial Orientation	Transversal

Geometry - Saturation

Fat suppr.	Fat sat.
Fat sat. mode	Strong
Water suppr.	None
Restore magn.	Off
Special sat.	Parallel F
Gap	10 mm
Thickness	70 mm

Geometry - Navigator

Geometry - Tim Planning Suite

Set-n-Go Protocol	Off
Table position	H
Table position	0 mm
Inline Composing	Off

System - Miscellaneous

Positioning mode	FIX
Table position	H
Table position	0 mm
MSMA	S - C - T
Sagittal	R >> L
Coronal	A >> P
Transversal	F >> H
Coil Combine Mode	Adaptive Combine
Save uncombined	Off
Matrix Optimization	Off
AutoAlign	Head > Brain
Coil Select Mode	Off - AutoCoilSelect

System - Adjustments

B0 Shim mode	Standard
B1 Shim mode	TrueForm
Adjust with body coil	Off
Confirm freq. adjustment	Off
Assume Dominant Fat	Off
Assume Silicone	Off
Adjustment Tolerance	Auto

System - Adjust Volume

Position	Isocenter
Orientation	Transversal
Rotation	90.00 deg
R >> L	240 mm
A >> P	240 mm
F >> H	144 mm
Reset	Off

System - pTx Volumes

B1 Shim mode	TrueForm

System - Tx/Rx

Frequency 1H	123.244475 MHz
Correction factor	1
Gain	High
Img. Scale Cor.	2.000
Reset	Off
? Ref. amplitude 1H	0.000 V

Physio - Signal1

1st Signal/Mode	None
TR	9500.0 ms
Concatenations	2

Physio - Cardiac

Magn. preparation	Slice-sel. IR
TI	2556 ms
Fat suppr.	Fat sat.
Dark blood	Off
FoV read	240 mm
FoV phase	100.0 %
BLADE coverage	100.0 %
Trajectory	BLADE

Physio - PACE

Resp. control	Off
Concatenations	2

Inline - Common

Subtract	Off
Measurements	1
StdDev	Off
Save original images	On

Inline - MIP

MIP-Sag	Off
MIP-Cor	Off
MIP-Tra	Off
MIP-Time	Off
Save original images	On

Inline - Composing

Inline Composing	Off
Distortion Corr.	Off

Sequence - Part 1

Introduction	On
Dimension	2D
Compensate T2 decay	Off
Contrasts	1
Flow comp.	Read
Multi-slice mode	Interleaved
Free echo spacing	Off
Echo spacing	8.26 ms
Bandwidth	362 Hz/Px

Sequence - Part 2

Define	Turbo factor
Echo trains per slice	9
Phase correction	Automatic
Acoustic noise reduction	None
RF pulse type	Low SAR
Gradient mode	Fast
Hyperecho	Off
WARP	Off
Motion correction	On
Red. EC sensitivity	Off
Turbo factor	28

Sequence - Assistant

Mode	Min flip angle
Min flip angle	130 deg
Allowed delay	30 s

\\Study Protocols\BRAIN\Other\TEBC_5 year old - E161723\DTI_rev_PA

TA: 0:29 PM: REF Voxel size: 2.0×2.0×2.0 mmPAT: 6 Rel. SNR: 1.00 : epse

Properties

Prio recon	Off
Load images to viewer	On
Inline movie	Off
Auto store images	On
Load images to stamp segments	Off
Load images to graphic segments	Off
Auto open inline display	Off
Auto close inline display	Off
Start measurement without further preparation	Off
Wait for user to start	Off
Start measurements	Single measurement

Routine

Slice group	1
Slices	63
Dist. factor	0 %
Position	L0.7 P3.0 H31.9 mm
Orientation	Transversal
Phase enc. dir.	P >> A
AutoAlign	---
Phase oversampling	0 %
FoV read	256 mm
FoV phase	100.0 %
Slice thickness	2.0 mm
TR	2800 ms
TE	82.0 ms
Concatenations	1
Filter	Raw filter, Prescan Normalize
Coil elements	HEA;HEP

Contrast - Common

TR	2800 ms
TE	82.0 ms
MTC	Off
Magn. preparation	None
Fat suppr.	Fat sat.
Fat sat. mode	Strong

Contrast - Dynamic

Averaging mode	Long term
Reconstruction	Magnitude
Measurements	1
Delay in TR	0 ms
Multiple series	Off

Resolution - Common

FoV read	256 mm
FoV phase	100.0 %
Slice thickness	2.0 mm
Base resolution	128
Phase resolution	100 %
Phase partial Fourier	7/8
Interpolation	Off

Resolution - iPAT

Accel. mode	Slice accel.
Accel. factor PE	2
Ref. lines PE	40

Resolution - iPAT

Accel. factor slice	3
Reference scan mode	EPI/separate

Resolution - Filter Image

Distortion Corr.	Off
Prescan Normalize	On
Dynamic Field Corr.	Off

Resolution - Filter Rawdata

Raw filter	On
Elliptical filter	Off

Geometry - Common

Slice group	1
Slices	63
Dist. factor	0 %
Position	L0.7 P3.0 H31.9 mm
Orientation	Transversal
Phase enc. dir.	P >> A
FoV read	256 mm
FoV phase	100.0 %
Slice thickness	2.0 mm
TR	2800 ms
Multi-slice mode	Interleaved
Series	Interleaved
Concatenations	1

Geometry - AutoAlign

Slice group	1
Position	L0.7 P3.0 H31.9 mm
Orientation	Transversal
Phase enc. dir.	P >> A
AutoAlign	---
Initial Position	L0.7 P3.0 H31.9
L	0.7 mm
P	3.0 mm
H	31.9 mm
Initial Rotation	-180.00 deg
Initial Orientation	Transversal

Geometry - Saturation

Fat suppr.	Fat sat.
Fat sat. mode	Strong
Special sat.	None

Geometry - Navigator

Geometry - Tim Planning Suite

Set-n-Go Protocol	Off
Table position	H
Table position	0 mm
Inline Composing	Off

System - Miscellaneous

Positioning mode	REF
Table position	H
Table position	0 mm
MSMA	S - C - T
Sagittal	R >> L

System - Miscellaneous

Coronal	A >> P
Transversal	H >> F
Coil Combine Mode	Adaptive Combine
Matrix Optimization	Performance
AutoAlign	---
Coil Select Mode	On - AutoCoilSelect

System - Adjustments

B0 Shim mode	Standard
B1 Shim mode	TrueForm
Adjust with body coil	Off
Confirm freq. adjustment	Off
Assume Dominant Fat	Off
Assume Silicone	Off
Adjustment Tolerance	Auto

System - Adjust Volume

Position	L0.7 P3.0 H31.9 mm
Orientation	Transversal
Rotation	180.00 deg
A >> P	256 mm
R >> L	256 mm
F >> H	126 mm
Reset	Off

System - pTx Volumes

B1 Shim mode	TrueForm
Excitation	Standard

System - Tx/Rx

Frequency 1H	123.244475 MHz
Correction factor	1
Gain	High
Img. Scale Cor.	2.000
Reset	Off
? Ref. amplitude 1H	0.000 V

Physio - Signal1

1st Signal/Mode	None
TR	2800 ms
Concatenations	1

Physio - PACE

Resp. control	Off
Concatenations	1

Diff - Neuro

Diffusion mode	Free
Diff. directions	151
Diffusion Scheme	Monopolar
Diff. weightings	1
b-value	0 s/mm²
b-value	3
Diff. weighted images	On
Trace weighted images	Off
ADC maps	Off
FA maps	Off
Mosaic	Off
Tensor	Off
Noise level	40

Diff - Body

Diffusion mode	Free

Diff - Body

Diff. directions	151
Diffusion Scheme	Monopolar
Diff. weightings	1
b-value	0 s/mm²
b-value	3
Diff. weighted images	On
Trace weighted images	Off
ADC maps	Off
Exponential ADC Maps	Off
FA maps	Off
Invert Gray Scale	Off
Calculated Image	Off
b-Value >=	0 s/mm²
Noise level	40

Diff - Composing

Inline Composing	Off
Distortion Corr.	Off

Sequence - Part 1

Introduction	On
Optimization	None
Multi-slice mode	Interleaved
Free echo spacing	Off
Echo spacing	0.93 ms
Bandwidth	1446 Hz/Px

Sequence - Part 2

EPI factor	128
RF pulse type	Low SAR
Gradient mode	Normal
Excitation	Standard

Sequence - pTX Pulses

\\Study Protocols\BRAIN\Other\TEBC_5 year old - E161723\DTI_AP

TA: 7:26 PM: REF Voxel size: 2.0×2.0×2.0 mmPAT: 6 Rel. SNR: 1.00 : epse

Properties

Prio recon	Off
Load images to viewer	On
Inline movie	Off
Auto store images	On
Load images to stamp segments	Off
Load images to graphic segments	Off
Auto open inline display	Off
Auto close inline display	Off
Start measurement without further preparation	Off
Wait for user to start	Off
Start measurements	Single measurement

Routine

Slice group	1
Slices	63
Dist. factor	0 %
Position	L0.7 P3.0 H31.9 mm
Orientation	Transversal
Phase enc. dir.	A >> P
AutoAlign	---
Phase oversampling	0 %
FoV read	256 mm
FoV phase	100.0 %
Slice thickness	2.0 mm
TR	2800 ms
TE	82.0 ms
Averages	1
Concatenations	1
Filter	Raw filter, Prescan Normalize
Coil elements	HEA;HEP

Contrast - Common

TR	2800 ms
TE	82.0 ms
MTC	Off
Magn. preparation	None
Fat suppr.	Fat sat.
Fat sat. mode	Strong

Contrast - Dynamic

Averages	1
Averaging mode	Long term
Reconstruction	Magnitude
Measurements	1
Delay in TR	0 ms
Multiple series	Off

Resolution - Common

FoV read	256 mm
FoV phase	100.0 %
Slice thickness	2.0 mm
Base resolution	128
Phase resolution	100 %
Phase partial Fourier	7/8
Interpolation	Off

Resolution - iPAT

Accel. mode	Slice accel.
Accel. factor PE	2
Ref. lines PE	40
Accel. factor slice	3
Reference scan mode	EPI/separate

Resolution - Filter Image

Distortion Corr.	Off
Prescan Normalize	On
Dynamic Field Corr.	Off

Resolution - Filter Rawdata

Raw filter	On
Elliptical filter	Off

Geometry - Common

Slice group	1
Slices	63
Dist. factor	0 %
Position	L0.7 P3.0 H31.9 mm
Orientation	Transversal
Phase enc. dir.	A >> P
FoV read	256 mm
FoV phase	100.0 %
Slice thickness	2.0 mm
TR	2800 ms
Multi-slice mode	Interleaved
Series	Interleaved
Concatenations	1

Geometry - AutoAlign

Slice group	1
Position	L0.7 P3.0 H31.9 mm
Orientation	Transversal
Phase enc. dir.	A >> P
AutoAlign	---
Initial Position	L0.7 P3.0 H31.9
L	0.7 mm
P	3.0 mm
H	31.9 mm
Initial Rotation	0.00 deg
Initial Orientation	Transversal

Geometry - Saturation

Fat suppr.	Fat sat.
Fat sat. mode	Strong
Special sat.	None

Geometry - Navigator

Geometry - Tim Planning Suite

Set-n-Go Protocol	Off
Table position	H
Table position	0 mm
Inline Composing	Off

System - Miscellaneous

Positioning mode	REF
Table position	H
Table position	0 mm

System - Miscellaneous

MSMA	S - C - T
Sagittal	R >> L
Coronal	A >> P
Transversal	H >> F
Coil Combine Mode	Adaptive Combine
Matrix Optimization	Performance
AutoAlign	---
Coil Select Mode	On - AutoCoilSelect

System - Adjustments

B0 Shim mode	Standard
B1 Shim mode	TrueForm
Adjust with body coil	Off
Confirm freq. adjustment	Off
Assume Dominant Fat	Off
Assume Silicone	Off
Adjustment Tolerance	Auto

System - Adjust Volume

Position	L0.7 P3.0 H31.9 mm
Orientation	Transversal
Rotation	0.00 deg
A >> P	256 mm
R >> L	256 mm
F >> H	126 mm
Reset	Off

System - pTx Volumes

B1 Shim mode	TrueForm
Excitation	Standard

System - Tx/Rx

Frequency 1H	123.244475 MHz
Correction factor	1
Gain	High
Img. Scale Cor.	2.000
Reset	Off
? Ref. amplitude 1H	0.000 V

Physio - Signal1

1st Signal/Mode	None
TR	2800 ms
Concatenations	1

Physio - PACE

Resp. control	Off
Concatenations	1

Diff - Neuro

Diffusion mode	Free
Diff. directions	151
Diffusion Scheme	Monopolar
Diff. weightings	2
b-value 1	0 s/mm²
b-value 2	2000 s/mm²
b-value 1	1
b-value 2	1
Diff. weighted images	On
Trace weighted images	On
ADC maps	On
FA maps	On
Mosaic	On
Tensor	Off

Diff - Neuro

Noise level	40

Diff - Body

Diffusion mode	Free
Diff. directions	151
Diffusion Scheme	Monopolar
Diff. weightings	2
b-value 1	0 s/mm²
b-value 2	2000 s/mm²
b-value 1	1
b-value 2	1
Diff. weighted images	On
Trace weighted images	On
ADC maps	On
Exponential ADC Maps	Off
FA maps	On
Invert Gray Scale	Off
Calculated Image	Off
b-Value >=	0 s/mm²
Noise level	40

Diff - Composing

Inline Composing	Off
Distortion Corr.	Off

Sequence - Part 1

Introduction	On
Optimization	None
Multi-slice mode	Interleaved
Free echo spacing	Off
Echo spacing	0.93 ms
Bandwidth	1446 Hz/Px

Sequence - Part 2

EPI factor	128
RF pulse type	Low SAR
Gradient mode	Normal
Excitation	Standard

Sequence - pTX Pulses

\\Study Protocols\BRAIN\Other\TEBC_5 year old - E161723\MTSatOn_5y
TA: 2:05 PM: FIX Voxel size: 1.6×1.6×1.6 mmPAT: 4 Rel. SNR: 1.00 : qfl

Properties

Prio recon	Off
Load images to viewer	On
Inline movie	Off
Auto store images	On
Load images to stamp segments	Off
Load images to graphic segments	Off
Auto open inline display	Off
Auto close inline display	Off
Start measurement without further preparation	Off
Wait for user to start	Off
Start measurements	Single measurement

Routine

Slab group	1
Slabs	1
Dist. factor	20 %
Position	Isocenter
Orientation	Sagittal
Phase enc. dir.	A >> P
AutoAlign	Head > Basis
Phase oversampling	0 %
Slice oversampling	0.0 %
Slices per slab	104
FoV read	205 mm
FoV phase	107.8 %
Slice thickness	1.60 mm
TR	35.0 ms
TE 1	2.29 ms
TE 2	6.33 ms
TE 3	10.37 ms
Averages	1
Concatenations	1
Filter	Prescan Normalize
Coil elements	HEA;HEP

Contrast - Common

TR	35.0 ms
TE 1	2.29 ms
TE 2	6.33 ms
TE 3	10.37 ms
MTC	On
Magn. preparation	None
Flip angle	5 deg
Fat suppr.	None
Water suppr.	None
SWI	Off

Contrast - Dynamic

Averages	1
Averaging mode	Short term
Reconstruction	Magnitude
Measurements	1
Multiple series	Each measurement

Resolution - Common

FoV read	205 mm
FoV phase	107.8 %
Slice thickness	1.60 mm
Base resolution	128

Resolution - Common

Phase resolution	100 %
Slice resolution	100 %
Phase partial Fourier	Off
Slice partial Fourier	7/8
Interpolation	Off

Resolution - iPAT

PAT mode	GRAPPA
Accel. factor PE	2
Ref. lines PE	24
Accel. factor 3D	2
Ref. lines 3D	24
Reference scan mode	Integrated

Resolution - Filter Image

Image Filter	Off
Distortion Corr.	Off
Prescan Normalize	On
Unfiltered images	Off
Normalize	Off
B1 filter	Off

Resolution - Filter Rawdata

Raw filter	Off
Elliptical filter	Off

Geometry - Common

Slab group	1
Slabs	1
Dist. factor	20 %
Position	Isocenter
Orientation	Sagittal
Phase enc. dir.	A >> P
Slice oversampling	0.0 %
Slices per slab	104
FoV read	205 mm
FoV phase	107.8 %
Slice thickness	1.60 mm
TR	35.0 ms
Multi-slice mode	Interleaved
Series	Interleaved
Concatenations	1

Geometry - AutoAlign

Slab group	1
Position	Isocenter
Orientation	Sagittal
Phase enc. dir.	A >> P
AutoAlign	Head > Basis
Initial Position	Isocenter
L	0.0 mm
P	0.0 mm
H	0.0 mm
Initial Rotation	0.00 deg
Initial Orientation	Sagittal

Geometry - Saturation

Saturation mode	Standard
Fat suppr.	None
Water suppr.	None

Geometry - Saturation

Special sat.	None

Geometry - Tim Planning Suite

Set-n-Go Protocol	Off
Table position	H
Table position	0 mm
Inline Composing	Off

System - Miscellaneous

Positioning mode	FIX
Table position	H
Table position	0 mm
MSMA	S - C - T
Sagittal	R >> L
Coronal	A >> P
Transversal	F >> H
Coil Combine Mode	Sum of Squares
Save uncombined	Off
Matrix Optimization	Off
AutoAlign	Head > Basis
Coil Select Mode	Off - AutoCoilSelect

System - Adjustments

B0 Shim mode	Tune up
B1 Shim mode	TrueForm
Adjust with body coil	Off
Confirm freq. adjustment	Off
Assume Dominant Fat	Off
Assume Silicone	Off
Adjustment Tolerance	Auto

System - Adjust Volume

Position	Isocenter
Orientation	Transversal
Rotation	0.00 deg
A >> P	263 mm
R >> L	350 mm
F >> H	350 mm
Reset	Off

System - pTx Volumes

B1 Shim mode	TrueForm
Excitation	Non-sel.

System - Tx/Rx

Frequency 1H	123.244475 MHz
Correction factor	1
Gain	Low
Img. Scale Cor.	3.000
Reset	Off
? Ref. amplitude 1H	0.000 V

Physio - Signal1

1st Signal/Mode	None
TR	35.0 ms
Concatenations	1
Segments	1

Physio - Cardiac

Tagging	None
Magn. preparation	None
Fat suppr.	None
Dark blood	Off

Physio - Cardiac

FoV read	205 mm
FoV phase	107.8 %
Phase resolution	100 %

Physio - PACE

Resp. control	Off
Concatenations	1

Inline - Common

Subtract	Off
Measurements	1
StdDev	Off
Liver registration	Off
Save original images	On

Inline - MIP

MIP-Sag	Off
MIP-Cor	Off
MIP-Tra	Off
MIP-Time	Off
Save original images	On

Inline - Soft Tissue

Wash - In	Off
Wash - Out	Off
TTP	Off
PEI	Off
MIP - time	Off
Measurements	1

Inline - Composing

Inline Composing	Off
Distortion Corr.	Off

Inline - MapIt

Save original images	On
MapIt	None
Flip angle	5 deg
Measurements	1
Contrasts	3
TR	35.0 ms
TE 1	2.29 ms
TE 2	6.33 ms
TE 3	10.37 ms

Sequence - Part 1

Introduction	On
Dimension	3D
Elliptical scanning	Off
Phase stabilisation	Off
Asymmetric echo	Off
Contrasts	3
Flow comp. 1	No
Readout mode	Monopolar
Multi-slice mode	Interleaved
Bandwidth 1	400 Hz/Px
Bandwidth 2	400 Hz/Px
Bandwidth 3	400 Hz/Px

Sequence - Part 2

Segments	1
Acoustic noise reduction	Active
RF pulse type	Low SAR

Sequence - Part 2

Gradient mode	Whisper
Excitation	Non-sel.
RF spoiling	On

Sequence - Assistant

Mode	Off
Allowed delay	30 s

\\Study Protocols\BRAIN\Other\TEBC_5 year old - E161723\MTSatOff_5y

TA: 2:05 PM: FIX Voxel size: 1.6×1.6×1.6 mmPAT: 4 Rel. SNR: 1.00 : qfl

Properties

Prio recon	Off
Load images to viewer	On
Inline movie	Off
Auto store images	On
Load images to stamp segments	Off
Load images to graphic segments	Off
Auto open inline display	Off
Auto close inline display	Off
Start measurement without further preparation	Off
Wait for user to start	Off
Start measurements	Single measurement

Routine

Slab group	1
Slabs	1
Dist. factor	20 %
Position	Isocenter
Orientation	Sagittal
Phase enc. dir.	A >> P
AutoAlign	Head > Basis
Phase oversampling	0 %
Slice oversampling	0.0 %
Slices per slab	104
FoV read	205 mm
FoV phase	107.8 %
Slice thickness	1.60 mm
TR	35.0 ms
TE 1	2.29 ms
TE 2	6.33 ms
TE 3	10.37 ms
Averages	1
Concatenations	1
Filter	Prescan Normalize
Coil elements	HEA;HEP

Contrast - Common

TR	35.0 ms
TE 1	2.29 ms
TE 2	6.33 ms
TE 3	10.37 ms
MTC	Off
Magn. preparation	None
Flip angle	5 deg
Fat suppr.	None
Water suppr.	None
SWI	Off

Contrast - Dynamic

Averages	1
Averaging mode	Short term
Reconstruction	Magnitude
Measurements	1
Multiple series	Each measurement

Resolution - Common

FoV read	205 mm
FoV phase	107.8 %
Slice thickness	1.60 mm
Base resolution	128

Resolution - Common

Phase resolution	100 %
Slice resolution	100 %
Phase partial Fourier	Off
Slice partial Fourier	7/8
Interpolation	Off

Resolution - iPAT

PAT mode	GRAPPA
Accel. factor PE	2
Ref. lines PE	24
Accel. factor 3D	2
Ref. lines 3D	24
Reference scan mode	Integrated

Resolution - Filter Image

Image Filter	Off
Distortion Corr.	Off
Prescan Normalize	On
Unfiltered images	Off
Normalize	Off
B1 filter	Off

Resolution - Filter Rawdata

Raw filter	Off
Elliptical filter	Off

Geometry - Common

Slab group	1
Slabs	1
Dist. factor	20 %
Position	Isocenter
Orientation	Sagittal
Phase enc. dir.	A >> P
Slice oversampling	0.0 %
Slices per slab	104
FoV read	205 mm
FoV phase	107.8 %
Slice thickness	1.60 mm
TR	35.0 ms
Multi-slice mode	Interleaved
Series	Interleaved
Concatenations	1

Geometry - AutoAlign

Slab group	1
Position	Isocenter
Orientation	Sagittal
Phase enc. dir.	A >> P
AutoAlign	Head > Basis
Initial Position	Isocenter
L	0.0 mm
P	0.0 mm
H	0.0 mm
Initial Rotation	0.00 deg
Initial Orientation	Sagittal

Geometry - Saturation

Saturation mode	Standard
Fat suppr.	None
Water suppr.	None

Geometry - Saturation

Special sat.	None

Geometry - Tim Planning Suite

Set-n-Go Protocol	Off
Table position	H
Table position	0 mm
Inline Composing	Off

System - Miscellaneous

Positioning mode	FIX
Table position	H
Table position	0 mm
MSMA	S - C - T
Sagittal	R >> L
Coronal	A >> P
Transversal	F >> H
Coil Combine Mode	Sum of Squares
Save uncombined	Off
Matrix Optimization	Off
AutoAlign	Head > Basis
Coil Select Mode	Off - AutoCoilSelect

System - Adjustments

B0 Shim mode	Tune up
B1 Shim mode	TrueForm
Adjust with body coil	Off
Confirm freq. adjustment	Off
Assume Dominant Fat	Off
Assume Silicone	Off
Adjustment Tolerance	Auto

System - Adjust Volume

Position	Isocenter
Orientation	Transversal
Rotation	0.00 deg
A >> P	263 mm
R >> L	350 mm
F >> H	350 mm
Reset	Off

System - pTx Volumes

B1 Shim mode	TrueForm
Excitation	Non-sel.

System - Tx/Rx

Frequency 1H	123.244475 MHz
Correction factor	1
Gain	Low
Img. Scale Cor.	3.000
Reset	Off
? Ref. amplitude 1H	0.000 V

Physio - Signal1

1st Signal/Mode	None
TR	35.0 ms
Concatenations	1
Segments	1

Physio - Cardiac

Tagging	None
Magn. preparation	None
Fat suppr.	None
Dark blood	Off

Physio - Cardiac

FoV read	205 mm
FoV phase	107.8 %
Phase resolution	100 %

Physio - PACE

Resp. control	Off
Concatenations	1

Inline - Common

Subtract	Off
Measurements	1
StdDev	Off
Liver registration	Off
Save original images	On

Inline - MIP

MIP-Sag	Off
MIP-Cor	Off
MIP-Tra	Off
MIP-Time	Off
Save original images	On

Inline - Soft Tissue

Wash - In	Off
Wash - Out	Off
TTP	Off
PEI	Off
MIP - time	Off
Measurements	1

Inline - Composing

Inline Composing	Off
Distortion Corr.	Off

Inline - MapIt

Save original images	On
MapIt	None
Flip angle	5 deg
Measurements	1
Contrasts	3
TR	35.0 ms
TE 1	2.29 ms
TE 2	6.33 ms
TE 3	10.37 ms

Sequence - Part 1

Introduction	On
Dimension	3D
Elliptical scanning	Off
Phase stabilisation	Off
Asymmetric echo	Off
Contrasts	3
Flow comp. 1	No
Readout mode	Monopolar
Multi-slice mode	Interleaved
Bandwidth 1	400 Hz/Px
Bandwidth 2	400 Hz/Px
Bandwidth 3	400 Hz/Px

Sequence - Part 2

Segments	1
Acoustic noise reduction	Active
RF pulse type	Low SAR

Sequence - Part 2

Gradient mode	Whisper
Excitation	Non-sel.
RF spoiling	On

Sequence - Assistant

Mode	Off
Allowed delay	30 s

\\Study Protocols\BRAIN\Other\TEBC_5 year old - E161723\MTSatT1w_5y
TA: 0:55 PM: FIX Voxel size: 1.6×1.6×1.6 mmPAT: 4 Rel. SNR: 1.00 : qfl

Properties

Prio recon	Off
Load images to viewer	On
Inline movie	Off
Auto store images	On
Load images to stamp segments	Off
Load images to graphic segments	Off
Auto open inline display	Off
Auto close inline display	Off
Start measurement without further preparation	Off
Wait for user to start	Off
Start measurements	Single measurement

Routine

Slab group	1
Slabs	1
Dist. factor	20 %
Position	Isocenter
Orientation	Sagittal
Phase enc. dir.	A >> P
AutoAlign	Head > Basis
Phase oversampling	0 %
Slice oversampling	0.0 %
Slices per slab	104
FoV read	205 mm
FoV phase	107.8 %
Slice thickness	1.60 mm
TR	15.0 ms
TE 1	2.29 ms
TE 2	6.33 ms
TE 3	10.37 ms
Averages	1
Concatenations	1
Filter	Prescan Normalize
Coil elements	HEA;HEP

Contrast - Common

TR	15.0 ms
TE 1	2.29 ms
TE 2	6.33 ms
TE 3	10.37 ms
MTC	Off
Magn. preparation	None
Flip angle	18 deg
Fat suppr.	None
Water suppr.	None
SWI	Off

Contrast - Dynamic

Averages	1
Averaging mode	Short term
Reconstruction	Magnitude
Measurements	1
Multiple series	Each measurement

Resolution - Common

FoV read	205 mm
FoV phase	107.8 %
Slice thickness	1.60 mm
Base resolution	128

Resolution - Common

Phase resolution	100 %
Slice resolution	100 %
Phase partial Fourier	Off
Slice partial Fourier	7/8
Interpolation	Off

Resolution - iPAT

PAT mode	GRAPPA
Accel. factor PE	2
Ref. lines PE	24
Accel. factor 3D	2
Ref. lines 3D	24
Reference scan mode	Integrated

Resolution - Filter Image

Image Filter	Off
Distortion Corr.	Off
Prescan Normalize	On
Unfiltered images	Off
Normalize	Off
B1 filter	Off

Resolution - Filter Rawdata

Raw filter	Off
Elliptical filter	Off

Geometry - Common

Slab group	1
Slabs	1
Dist. factor	20 %
Position	Isocenter
Orientation	Sagittal
Phase enc. dir.	A >> P
Slice oversampling	0.0 %
Slices per slab	104
FoV read	205 mm
FoV phase	107.8 %
Slice thickness	1.60 mm
TR	15.0 ms
Multi-slice mode	Interleaved
Series	Interleaved
Concatenations	1

Geometry - AutoAlign

Slab group	1
Position	Isocenter
Orientation	Sagittal
Phase enc. dir.	A >> P
AutoAlign	Head > Basis
Initial Position	Isocenter
L	0.0 mm
P	0.0 mm
H	0.0 mm
Initial Rotation	0.00 deg
Initial Orientation	Sagittal

Geometry - Saturation

Saturation mode	Standard
Fat suppr.	None
Water suppr.	None

Geometry - Saturation

Special sat.	None

Geometry - Tim Planning Suite

Set-n-Go Protocol	Off
Table position	H
Table position	0 mm
Inline Composing	Off

System - Miscellaneous

Positioning mode	FIX
Table position	H
Table position	0 mm
MSMA	S - C - T
Sagittal	R >> L
Coronal	A >> P
Transversal	F >> H
Coil Combine Mode	Sum of Squares
Save uncombined	Off
Matrix Optimization	Off
AutoAlign	Head > Basis
Coil Select Mode	Off - AutoCoilSelect

System - Adjustments

B0 Shim mode	Tune up
B1 Shim mode	TrueForm
Adjust with body coil	Off
Confirm freq. adjustment	Off
Assume Dominant Fat	Off
Assume Silicone	Off
Adjustment Tolerance	Auto

System - Adjust Volume

Position	Isocenter
Orientation	Transversal
Rotation	0.00 deg
A >> P	263 mm
R >> L	350 mm
F >> H	350 mm
Reset	Off

System - pTx Volumes

B1 Shim mode	TrueForm
Excitation	Non-sel.

System - Tx/Rx

Frequency 1H	123.244475 MHz
Correction factor	1
Gain	Low
Img. Scale Cor.	3.000
Reset	Off
? Ref. amplitude 1H	0.000 V

Physio - Signal1

1st Signal/Mode	None
TR	15.0 ms
Concatenations	1
Segments	1

Physio - Cardiac

Tagging	None
Magn. preparation	None
Fat suppr.	None
Dark blood	Off

Physio - Cardiac

FoV read	205 mm
FoV phase	107.8 %
Phase resolution	100 %

Physio - PACE

Resp. control	Off
Concatenations	1

Inline - Common

Subtract	Off
Measurements	1
StdDev	Off
Liver registration	Off
Save original images	On

Inline - MIP

MIP-Sag	Off
MIP-Cor	Off
MIP-Tra	Off
MIP-Time	Off
Save original images	On

Inline - Soft Tissue

Wash - In	Off
Wash - Out	Off
TTP	Off
PEI	Off
MIP - time	Off
Measurements	1

Inline - Composing

Inline Composing	Off
Distortion Corr.	Off

Inline - MapIt

Save original images	On
MapIt	None
Flip angle	18 deg
Measurements	1
Contrasts	3
TR	15.0 ms
TE 1	2.29 ms
TE 2	6.33 ms
TE 3	10.37 ms

Sequence - Part 1

Introduction	On
Dimension	3D
Elliptical scanning	Off
Phase stabilisation	Off
Asymmetric echo	Off
Contrasts	3
Flow comp. 1	No
Readout mode	Monopolar
Multi-slice mode	Interleaved
Bandwidth 1	400 Hz/Px
Bandwidth 2	400 Hz/Px
Bandwidth 3	400 Hz/Px

Sequence - Part 2

Segments	1
Acoustic noise reduction	Active
RF pulse type	Low SAR

Sequence - Part 2

Gradient mode	Whisper
Excitation	Non-sel.
RF spoiling	On

Sequence - Assistant

Mode	Off
Allowed delay	30 s

\\Study Protocols\BRAIN\Other\TEBC_5 year old - E161723\gre_field_mapping_3mm
TA: 1:18 PM: FIX Voxel size: 2.3×2.3×2.3 mmRel. SNR: 1.00 : fm_r

Properties

Prio recon	Off
Load images to viewer	On
Inline movie	Off
Auto store images	On
Load images to stamp segments	Off
Load images to graphic segments	Off
Auto open inline display	Off
Auto close inline display	Off
Start measurement without further preparation	Off
Wait for user to start	Off
Start measurements	Single measurement

Routine

Slice group	1
Slices	45
Dist. factor	20 %
Position	Isocenter
Orientation	Transversal
Phase enc. dir.	R >> L
AutoAlign	Head > Brain
Phase oversampling	0 %
FoV read	193 mm
FoV phase	100.0 %
Slice thickness	2.3 mm
TR	450.0 ms
TE 1	4.92 ms
TE 2	7.38 ms
Averages	1
Concatenations	1
Filter	None
Coil elements	HEA;HEP

Contrast - Common

TR	450.0 ms
TE 1	4.92 ms
TE 2	7.38 ms
MTC	Off
Flip angle	60 deg
Fat suppr.	None

Contrast - Dynamic

Averages	1
Averaging mode	Long term
Reconstruction	Magn./Phase
Measurements	1
Multiple series	Off

Resolution - Common

FoV read	193 mm
FoV phase	100.0 %
Slice thickness	2.3 mm
Base resolution	84
Phase resolution	100 %
Phase partial Fourier	Off
Interpolation	Off

Resolution - Filter Image

Image Filter	Off
Distortion Corr.	Off

Resolution - Filter Image

Prescan Normalize	Off
Normalize	Off
B1 filter	Off

Resolution - Filter Rawdata

Raw filter	Off
Elliptical filter	Off

Geometry - Common

Slice group	1
Slices	45
Dist. factor	20 %
Position	Isocenter
Orientation	Transversal
Phase enc. dir.	R >> L
FoV read	193 mm
FoV phase	100.0 %
Slice thickness	2.3 mm
TR	450.0 ms
Multi-slice mode	Interleaved
Series	Interleaved
Concatenations	1

Geometry - AutoAlign

Slice group	1
Position	Isocenter
Orientation	Transversal
Phase enc. dir.	R >> L
AutoAlign	Head > Brain
Initial Position	Isocenter
L	0.0 mm
P	0.0 mm
H	0.0 mm
Initial Rotation	90.00 deg
Initial Orientation	Transversal

Geometry - Saturation

Fat suppr.	None
Special sat.	None

Geometry - Tim Planning Suite

Set-n-Go Protocol	Off
Table position	H
Table position	0 mm
Inline Composing	Off

System - Miscellaneous

Positioning mode	FIX
Table position	H
Table position	0 mm
MSMA	S - C - T
Sagittal	R >> L
Coronal	A >> P
Transversal	F >> H
Coil Combine Mode	Sum of Squares
Save uncombined	Off
Matrix Optimization	Off
AutoAlign	Head > Brain
Coil Select Mode	Off - AutoCoilSelect

System - Adjustments

B0 Shim mode	Standard
B1 Shim mode	TrueForm
Adjust with body coil	Off
Confirm freq. adjustment	Off
Assume Dominant Fat	Off
Assume Silicone	Off
Adjustment Tolerance	Auto

System - Adjust Volume

Position	Isocenter
Orientation	Transversal
Rotation	90.00 deg
R >> L	193 mm
A >> P	193 mm
F >> H	124 mm
Reset	Off

System - pTx Volumes

B1 Shim mode	TrueForm

System - Tx/Rx

Frequency 1H	123.244475 MHz
Correction factor	1
Gain	High
Img. Scale Cor.	1.000
Reset	Off
? Ref. amplitude 1H	0.000 V

Sequence - Part 1

Introduction	On
Dimension	2D
Asymmetric echo	Off
Contrasts	2
Flow comp.	Yes
Multi-slice mode	Interleaved
Bandwidth	595 Hz/Px

Sequence - Part 2

RF pulse type	Normal
Gradient mode	Fast
RF spoiling	On

Sequence - Assistant

Mode	Off

\\Study Protocols\BRAIN\Other\TEBC_5 year old - E161723\ep2d_p2_s3_AP_pixar-1

TA: 5:34 PM: FIX Voxel size: 2.3×2.3×2.3 mmPAT: 6 Rel. SNR: 1.00 : epfid

Properties

Prio recon	Off
Load images to viewer	On
Inline movie	Off
Auto store images	On
Load images to stamp segments	Off
Load images to graphic segments	Off
Auto open inline display	Off
Auto close inline display	Off
Start measurement without further preparation	Off
Wait for user to start	On
Start measurements	Single measurement

Routine

Slice group	1
Slices	45
Dist. factor	20 %
Position	Isocenter
Orientation	Transversal
Phase enc. dir.	A >> P
AutoAlign	Head > Brain
Phase oversampling	0 %
FoV read	193 mm
FoV phase	100.0 %
Slice thickness	2.3 mm
TR	1000 ms
TE	30.0 ms
Averages	1
Concatenations	1
Filter	None
Coil elements	HEA;HEP

Contrast - Common

TR	1000 ms
TE	30.0 ms
MTC	Off
Flip angle	60 deg
Fat suppr.	Fat sat.

Contrast - Dynamic

Averages	1
Averaging mode	Long term
Reconstruction	Magnitude
Measurements	324
Delay in TR	0 ms
Multiple series	Off

Resolution - Common

FoV read	193 mm
FoV phase	100.0 %
Slice thickness	2.3 mm
Base resolution	84
Phase resolution	100 %
Phase partial Fourier	Off
Interpolation	Off

Resolution - iPAT

Accel. mode	Slice accel.
Accel. factor PE	2
Ref. lines PE	24

Resolution - iPAT

Accel. factor slice	3
Reference scan mode	EPI/separate

Resolution - Filter Image

Distortion Corr.	Off
Prescan Normalize	Off

Resolution - Filter Rawdata

Raw filter	Off
Elliptical filter	Off
Hamming	Off

Geometry - Common

Slice group	1
Slices	45
Dist. factor	20 %
Position	Isocenter
Orientation	Transversal
Phase enc. dir.	A >> P
FoV read	193 mm
FoV phase	100.0 %
Slice thickness	2.3 mm
TR	1000 ms
Multi-slice mode	Interleaved
Series	Interleaved
Concatenations	1

Geometry - AutoAlign

Slice group	1
Position	Isocenter
Orientation	Transversal
Phase enc. dir.	A >> P
AutoAlign	Head > Brain
Initial Position	Isocenter
L	0.0 mm
P	0.0 mm
H	0.0 mm
Initial Rotation	0.00 deg
Initial Orientation	Transversal

Geometry - Saturation

Fat suppr.	Fat sat.
Special sat.	None

Geometry - Tim Planning Suite

Set-n-Go Protocol	Off
Table position	H
Table position	0 mm
Inline Composing	Off

System - Miscellaneous

Positioning mode	FIX
Table position	H
Table position	0 mm
MSMA	S - C - T
Sagittal	R >> L
Coronal	A >> P
Transversal	F >> H
Coil Combine Mode	Sum of Squares
Matrix Optimization	Off

System - Miscellaneous

AutoAlign	Head > Brain
Coil Select Mode	Off - AutoCoilSelect

System - Adjustments

B0 Shim mode	Standard
B1 Shim mode	TrueForm
Adjust with body coil	Off
Confirm freq. adjustment	Off
Assume Dominant Fat	Off
Assume Silicone	Off
Adjustment Tolerance	Auto

System - Adjust Volume

Position	Isocenter
Orientation	Transversal
Rotation	0.00 deg
A >> P	193 mm
R >> L	193 mm
F >> H	124 mm
Reset	Off

System - pTx Volumes

B1 Shim mode	TrueForm
Excitation	Standard

System - Tx/Rx

Frequency 1H	123.244475 MHz
Correction factor	1
Gain	High
Img. Scale Cor.	1.500
Reset	Off
? Ref. amplitude 1H	0.000 V

Physio - Signal1

1st Signal/Mode	None
TR	1000 ms
Concatenations	1

BOLD

GLM Statistics	Off
Dynamic t-maps	Off
Ignore meas. at start	0
Ignore after transition	0
Model transition states	On
Temp. highpass filter	On
Threshold	4.00
Paradigm size	40
Meas[1]	Baseline
Meas[2]	Baseline
Meas[3]	Baseline
Meas[4]	Baseline
Meas[5]	Baseline
Meas[6]	Baseline
Meas[7]	Baseline
Meas[8]	Baseline
Meas[9]	Baseline
Meas[10]	Baseline
Meas[11]	Baseline
Meas[12]	Baseline
Meas[13]	Baseline
Meas[14]	Baseline
Meas[15]	Baseline
Meas[16]	Baseline
Meas[17]	Baseline

BOLD

Meas[18]	Baseline
Meas[19]	Baseline
Meas[20]	Baseline
Meas[21]	Active
Meas[22]	Active
Meas[23]	Active
Meas[24]	Active
Meas[25]	Active
Meas[26]	Active
Meas[27]	Active
Meas[28]	Active
Meas[29]	Active
Meas[30]	Active
Meas[31]	Active
Meas[32]	Active
Meas[33]	Active
Meas[34]	Active
Meas[35]	Active
Meas[36]	Active
Meas[37]	Active
Meas[38]	Active
Meas[39]	Active
Meas[40]	Active
Motion correction	Off
Spatial filter	Off
Measurements	324
Delay in TR	0 ms
Multiple series	Off

Sequence - Part 1

Introduction	Off
Multi-slice mode	Interleaved
Free echo spacing	Off
Echo spacing	0.7 ms
Bandwidth	2290 Hz/Px

Sequence - Part 2

EPI factor	84
RF pulse type	Normal
Gradient mode	Normal
Excitation	Standard

Sequence - pTX Pulses

\\Study Protocols\BRAIN\Other\TEBC_5 year old - E161723\ep2d_p2_s3_AP_pixar-2
TA: 5:34 PM: FIX Voxel size: 2.3×2.3×2.3 mmPAT: 6 Rel. SNR: 1.00 : epfid

Properties

Prio recon	Off
Load images to viewer	On
Inline movie	Off
Auto store images	On
Load images to stamp segments	Off
Load images to graphic segments	Off
Auto open inline display	Off
Auto close inline display	Off
Start measurement without further preparation	Off
Wait for user to start	On
Start measurements	Single measurement

Routine

Slice group	1
Slices	45
Dist. factor	20 %
Position	Isocenter
Orientation	Transversal
Phase enc. dir.	A >> P
AutoAlign	Head > Brain
Phase oversampling	0 %
FoV read	193 mm
FoV phase	100.0 %
Slice thickness	2.3 mm
TR	1000 ms
TE	30.0 ms
Averages	1
Concatenations	1
Filter	None
Coil elements	HEA;HEP

Contrast - Common

TR	1000 ms
TE	30.0 ms
MTC	Off
Flip angle	60 deg
Fat suppr.	Fat sat.

Contrast - Dynamic

Averages	1
Averaging mode	Long term
Reconstruction	Magnitude
Measurements	324
Delay in TR	0 ms
Multiple series	Off

Resolution - Common

FoV read	193 mm
FoV phase	100.0 %
Slice thickness	2.3 mm
Base resolution	84
Phase resolution	100 %
Phase partial Fourier	Off
Interpolation	Off

Resolution - iPAT

Accel. mode	Slice accel.
Accel. factor PE	2
Ref. lines PE	24

Resolution - iPAT

Accel. factor slice	3
Reference scan mode	EPI/separate

Resolution - Filter Image

Distortion Corr.	Off
Prescan Normalize	Off

Resolution - Filter Rawdata

Raw filter	Off
Elliptical filter	Off
Hamming	Off

Geometry - Common

Slice group	1
Slices	45
Dist. factor	20 %
Position	Isocenter
Orientation	Transversal
Phase enc. dir.	A >> P
FoV read	193 mm
FoV phase	100.0 %
Slice thickness	2.3 mm
TR	1000 ms
Multi-slice mode	Interleaved
Series	Interleaved
Concatenations	1

Geometry - AutoAlign

Slice group	1
Position	Isocenter
Orientation	Transversal
Phase enc. dir.	A >> P
AutoAlign	Head > Brain
Initial Position	Isocenter
L	0.0 mm
P	0.0 mm
H	0.0 mm
Initial Rotation	0.00 deg
Initial Orientation	Transversal

Geometry - Saturation

Fat suppr.	Fat sat.
Special sat.	None

Geometry - Tim Planning Suite

Set-n-Go Protocol	Off
Table position	H
Table position	0 mm
Inline Composing	Off

System - Miscellaneous

Positioning mode	FIX
Table position	H
Table position	0 mm
MSMA	S - C - T
Sagittal	R >> L
Coronal	A >> P
Transversal	F >> H
Coil Combine Mode	Sum of Squares
Matrix Optimization	Off

System - Miscellaneous

AutoAlign	Head > Brain
Coil Select Mode	Off - AutoCoilSelect

System - Adjustments

B0 Shim mode	Standard
B1 Shim mode	TrueForm
Adjust with body coil	Off
Confirm freq. adjustment	Off
Assume Dominant Fat	Off
Assume Silicone	Off
Adjustment Tolerance	Auto

System - Adjust Volume

Position	Isocenter
Orientation	Transversal
Rotation	0.00 deg
A >> P	193 mm
R >> L	193 mm
F >> H	124 mm
Reset	Off

System - pTx Volumes

B1 Shim mode	TrueForm
Excitation	Standard

System - Tx/Rx

Frequency 1H	123.244475 MHz
Correction factor	1
Gain	High
Img. Scale Cor.	1.500
Reset	Off
? Ref. amplitude 1H	0.000 V

Physio - Signal1

1st Signal/Mode	None
TR	1000 ms
Concatenations	1

BOLD

GLM Statistics	Off
Dynamic t-maps	Off
Ignore meas. at start	0
Ignore after transition	0
Model transition states	On
Temp. highpass filter	On
Threshold	4.00
Paradigm size	40
Meas[1]	Baseline
Meas[2]	Baseline
Meas[3]	Baseline
Meas[4]	Baseline
Meas[5]	Baseline
Meas[6]	Baseline
Meas[7]	Baseline
Meas[8]	Baseline
Meas[9]	Baseline
Meas[10]	Baseline
Meas[11]	Baseline
Meas[12]	Baseline
Meas[13]	Baseline
Meas[14]	Baseline
Meas[15]	Baseline
Meas[16]	Baseline
Meas[17]	Baseline

BOLD

Meas[18]	Baseline
Meas[19]	Baseline
Meas[20]	Baseline
Meas[21]	Active
Meas[22]	Active
Meas[23]	Active
Meas[24]	Active
Meas[25]	Active
Meas[26]	Active
Meas[27]	Active
Meas[28]	Active
Meas[29]	Active
Meas[30]	Active
Meas[31]	Active
Meas[32]	Active
Meas[33]	Active
Meas[34]	Active
Meas[35]	Active
Meas[36]	Active
Meas[37]	Active
Meas[38]	Active
Meas[39]	Active
Meas[40]	Active
Motion correction	Off
Spatial filter	Off
Measurements	324
Delay in TR	0 ms
Multiple series	Off

Sequence - Part 1

Introduction	Off
Multi-slice mode	Interleaved
Free echo spacing	Off
Echo spacing	0.7 ms
Bandwidth	2290 Hz/Px

Sequence - Part 2

EPI factor	84
RF pulse type	Normal
Gradient mode	Normal
Excitation	Standard

Sequence - pTX Pulses

\\Study Protocols\BRAIN\Other\TEBC_5 year old - E161723\ep2d_p2_s3_AP_sesame
TA: 6:44 PM: FIX Voxel size: 2.3×2.3×2.3 mmPAT: 6 Rel. SNR: 1.00 : epfid

Properties

Prio recon	Off
Load images to viewer	On
Inline movie	Off
Auto store images	On
Load images to stamp segments	Off
Load images to graphic segments	Off
Auto open inline display	Off
Auto close inline display	Off
Start measurement without further preparation	Off
Wait for user to start	On
Start measurements	Single measurement

Routine

Slice group	1
Slices	45
Dist. factor	20 %
Position	Isocenter
Orientation	Transversal
Phase enc. dir.	A >> P
AutoAlign	Head > Brain
Phase oversampling	0 %
FoV read	193 mm
FoV phase	100.0 %
Slice thickness	2.3 mm
TR	1000 ms
TE	30.0 ms
Averages	1
Concatenations	1
Filter	None
Coil elements	HEA;HEP

Contrast - Common

TR	1000 ms
TE	30.0 ms
MTC	Off
Flip angle	60 deg
Fat suppr.	Fat sat.

Contrast - Dynamic

Averages	1
Averaging mode	Long term
Reconstruction	Magnitude
Measurements	394
Delay in TR	0 ms
Multiple series	Off

Resolution - Common

FoV read	193 mm
FoV phase	100.0 %
Slice thickness	2.3 mm
Base resolution	84
Phase resolution	100 %
Phase partial Fourier	Off
Interpolation	Off

Resolution - iPAT

Accel. mode	Slice accel.
Accel. factor PE	2
Ref. lines PE	24

Resolution - iPAT

Accel. factor slice	3
Reference scan mode	EPI/separate

Resolution - Filter Image

Distortion Corr.	Off
Prescan Normalize	Off

Resolution - Filter Rawdata

Raw filter	Off
Elliptical filter	Off
Hamming	Off

Geometry - Common

Slice group	1
Slices	45
Dist. factor	20 %
Position	Isocenter
Orientation	Transversal
Phase enc. dir.	A >> P
FoV read	193 mm
FoV phase	100.0 %
Slice thickness	2.3 mm
TR	1000 ms
Multi-slice mode	Interleaved
Series	Interleaved
Concatenations	1

Geometry - AutoAlign

Slice group	1
Position	Isocenter
Orientation	Transversal
Phase enc. dir.	A >> P
AutoAlign	Head > Brain
Initial Position	Isocenter
L	0.0 mm
P	0.0 mm
H	0.0 mm
Initial Rotation	0.00 deg
Initial Orientation	Transversal

Geometry - Saturation

Fat suppr.	Fat sat.
Special sat.	None

Geometry - Tim Planning Suite

Set-n-Go Protocol	Off
Table position	H
Table position	0 mm
Inline Composing	Off

System - Miscellaneous

Positioning mode	FIX
Table position	H
Table position	0 mm
MSMA	S - C - T
Sagittal	R >> L
Coronal	A >> P
Transversal	F >> H
Coil Combine Mode	Sum of Squares
Matrix Optimization	Off

System - Miscellaneous

AutoAlign	Head > Brain
Coil Select Mode	Off - AutoCoilSelect

System - Adjustments

B0 Shim mode	Standard
B1 Shim mode	TrueForm
Adjust with body coil	Off
Confirm freq. adjustment	Off
Assume Dominant Fat	Off
Assume Silicone	Off
Adjustment Tolerance	Auto

System - Adjust Volume

Position	Isocenter
Orientation	Transversal
Rotation	0.00 deg
A >> P	193 mm
R >> L	193 mm
F >> H	124 mm
Reset	Off

System - pTx Volumes

B1 Shim mode	TrueForm
Excitation	Standard

System - Tx/Rx

Frequency 1H	123.244475 MHz
Correction factor	1
Gain	High
Img. Scale Cor.	1.500
Reset	Off
? Ref. amplitude 1H	0.000 V

Physio - Signal1

1st Signal/Mode	None
TR	1000 ms
Concatenations	1

BOLD

GLM Statistics	Off
Dynamic t-maps	Off
Ignore meas. at start	0
Ignore after transition	0
Model transition states	On
Temp. highpass filter	On
Threshold	4.00
Paradigm size	40
Meas[1]	Baseline
Meas[2]	Baseline
Meas[3]	Baseline
Meas[4]	Baseline
Meas[5]	Baseline
Meas[6]	Baseline
Meas[7]	Baseline
Meas[8]	Baseline
Meas[9]	Baseline
Meas[10]	Baseline
Meas[11]	Baseline
Meas[12]	Baseline
Meas[13]	Baseline
Meas[14]	Baseline
Meas[15]	Baseline
Meas[16]	Baseline
Meas[17]	Baseline

BOLD

Meas[18]	Baseline
Meas[19]	Baseline
Meas[20]	Baseline
Meas[21]	Active
Meas[22]	Active
Meas[23]	Active
Meas[24]	Active
Meas[25]	Active
Meas[26]	Active
Meas[27]	Active
Meas[28]	Active
Meas[29]	Active
Meas[30]	Active
Meas[31]	Active
Meas[32]	Active
Meas[33]	Active
Meas[34]	Active
Meas[35]	Active
Meas[36]	Active
Meas[37]	Active
Meas[38]	Active
Meas[39]	Active
Meas[40]	Active
Motion correction	Off
Spatial filter	Off
Measurements	394
Delay in TR	0 ms
Multiple series	Off

Sequence - Part 1

Introduction	Off
Multi-slice mode	Interleaved
Free echo spacing	Off
Echo spacing	0.7 ms
Bandwidth	2290 Hz/Px

Sequence - Part 2

EPI factor	84
RF pulse type	Normal
Gradient mode	Normal
Excitation	Standard

Sequence - pTX Pulses
