# Supplementary material for: Preterm birth as a determinant of neurodevelopment and cognition in children (PRENCOG): protocol for an exposure-based cohort study in the UK
Source: BMJ Open. 2024 Sep 16;14(9):e085365. doi: 10.1136/bmjopen-2024-085365 (PMC11409314; doi:10.1136/bmjopen-2024-085365)
Supplement: Supplementary file 3 [file bmjopen-2024-085365.reviewer_comments.pdf]

PEER REVIEW HISTORY

BMJ Open publishes all reviews undertaken for accepted manuscripts. Reviewers are asked to complete a checklist review form and are provided with free text boxes to elaborate on their assessment. These free text comments are reproduced below.

ARTICLE DETAILS

**TITLE (PROVISIONAL)**	Preterm birth as a determinant of neurodevelopment and cognition in children (PRENCOG): protocol for an exposure-based cohort study in the United Kingdom.
**AUTHORS**	Boardman, James; Andrew, Ruth; Bastin, Mark; Battersby, Cheryl; Batty, George; Cábez, Manuel Blesa; Cox, Simon; Hall, Jill; Ingledow, Lauren; Marioni, Riccardo; Modi, Neena; Murphy, Lee; Quigley, Alan J.; Reynolds, Rebecca; Richardson, Hilary; Stock, Sarah; Thrippleton, Michael; Tsanas, Athanasios; Whalley, Heather

VERSION 1 - REVIEW

**REVIEWER NAME**	Pierrat, Veronique
**REVIEWER AFFILIATION**	Université de Paris, CRESS, INSERM, INRA, F-75004 Paris, France, Equipe EPOPé U 1153
**REVIEWER CONFLICT OF INTEREST**	None
**DATE REVIEW RETURNED**	07-Apr-2024

**GENERAL COMMENTS**	The research question is very relevant and the protocol is clearly described. I have only minor comments.<br>Could you include the date for the start of data collection?<br>In the abstract I was wondering what kind of behavioural traits could be collected in the neonatal period and I could not find the answer in the methods section.<br>Study population: the exclusion criteria state that infants with a contraindication to MRI at 3 Tesla were excluded. These contraindications are so exceptional in the neonatal period that I tried to find some in the literature that I was not aware of. The NHS has published recommendations. Are you referring to the NHS contraindications? They are not really applicable to neonates, but I can understand that these exclusion criteria should be mentioned in a protocol.<br>Finally, I have a personal question about the control group. Polluted environments are increasingly being described as risk factors for neurodevelopmental disorders. Do you think you will be able to control for this?<br>In conclusion, the research question is highly relevant with a strong protocol. Conclusions could help to redefine the goals of care for preterm infants and have a significant impact on outcomes.

**REVIEWER NAME**	Arabin, Birgit
**REVIEWER AFFILIATION**	Humboldt University of Berlin, Charite University Berlin

**REVIEWER CONFLICT OF INTEREST**	None
**DATE REVIEW RETURNED**	15-Apr-2024

**GENERAL COMMENTS**	GENERAL OVERVIEW, ORIGINALITY<br>The present paper summarizes an extensive multidisciplinary research protocol on the mechanisms and causal evidence of neurodevelopmental and cognitive impairment associated with early prematurity. The design is an exposure-based prospective cohort study. Another advantage is that data from the UK National Neonatal Research Database and the National Pupil Database can be linked.<br>Preterm birth (PTB) is not only a syndrome with many causes, even the pre-and postnatal consequences of prematurity for the developing brain may have different pathways which are important to modify neurocognitive development by specific interventions. Therefore, this project intends to cover the knowledge gap in how far harmful effects up to adulthood are due to changes in maternal and fetal, respectively neonatal cortisol, social factors or the interrelated brain metabolism and brain morphology. PTB is the leading cause of perinatal and infant mortality, accounting for approximately one-third of newborn deaths. Among survivors, short-term complications and the risk for long-term neurodevelopmental (but also cardiovascular, immunological and metabolic) diseases are globally increased. Therefore, this project is of elementary importance possibly interrupting vicious circles of social and global inequality,<br><br>STEPWISE APPROACH<br>ABSTRACT: The abstract clearly describes the research goals – but according to the editorial advice the authors should clearly explain the dates when the study starts (or has been started). Similarly, it is unclear from the abstract at what postnatal age certain examinations from the neonates, respectively from the infants are collected. The authors should add this information.<br>STRENGTHS AND LIMITATIONS: Accurately described – the limitation that the project is restricted to high-income settings is understandable but leaves us with uncertainties in how far this can be of relevance for low/medium-income settings.<br>INTRODUCTION/BACKGROUND: Clear<br>RATIONAL FOR STUDY. The authors discuss that it is not necessarily prematurity per se harming the development, anatomy and function of brain structures but associated factors of prematurity such as maternal stress or systemic inflammation already acquired pre- or postnatally. Several measurements will be performed such as glucocorticoids in maternal and neonatal hair or indirect determination of the activity of 11-ß dehydrogenase or DNA methylation. It might also be an option to determine pro-inflammatory markers and even the telomere length in the maternal and umbilical blood (see Lazarides C, et al. Maternal pro-inflammatory state during pregnancy and newborn leukocyte telomere length: A prospective investigation. Brain Behav Immun. 2019;80:419–26 or Verner G, et al. Maternal psychological resilience during pregnancy and newborn telomere length: a prospective study. Am J Psychiatry. 2021;178(2):183–92)<br>METHODS: The authors describe their methods in detail by text and Table 1. However, it is not clear at what postnatal age the neuroimaging is scheduled. This might depend on feasibility due to

	neonatal health. Nevertheless, there should be some rough description.<br>Did the authors also consider prenatal neuroimaging (see: Wu Y, et al. Association of prenatal maternal psychological distress with fetal brain growth, metabolism, and cortical maturation. JAMA Netw Open. 2020;3(1):e1919940)? It has been demonstrated that maternal stress (frequent in women admitted due to threatening labor, the size of several parts of the fetal brain and brain metabolism are all reduced. It might be interesting to compare the results before and after delivery.<br><br>CONCLUSION<br>The study protocol is original, well-written and deserves publication. The content of the research is essential. Nevertheless, the timing of some examinations, mainly neuroimaging, gut microbiome etc. and the follow-up of these children after the neonatal period should be clarified more clearly.

VERSION 1 – AUTHOR RESPONSE

Reviewer: 1
Dr. Veronique Pierrat, Université de Paris, CRESS, INSERM, INRA, F-75004 Paris, France, CHU Lille
Comments to the Author:
The research question is very relevant and the protocol is clearly described. I have only minor comments.
Could you include the date for the start of data collection?

Response 5. Please see response 3.

In the abstract I was wondering what kind of behavioural traits could be collected in the neonatal period and I could not find the answer in the methods section.

Response 6. The behavioural data collected at the neonatal time point are listed in Table 1 in Methods: Edinburgh postnatal depression scale, parenting daily hassles, World Health Organisation-Quality of Life, Adult temperament questionnaire. We appreciate the word 'behaviour' could imply that direct measures of neonatal behaviour are part of the research protocol, so we have removed this term. The sentence now reads:

'We will collect parental and infant medical, demographic, socioeconomic characteristics, and biological data which include placental tissue, umbilical cord blood, maternal and infant hair, infant saliva, infant dried blood spots, faecal material, and structural and diffusion MRI.'

Study population: the exclusion criteria state that infants with a contraindication to MRI at 3 Tesla were excluded. These contraindications are so exceptional in the neonatal period that I tried to find some in the literature that I was not aware of. The NHS has published recommendations. Are you

referring to the NHS contraindications? They are not really applicable to neonates, but I can understand that these exclusion criteria should be mentioned in a protocol.

Response 7. The reviewer is correct: we are referring to generally accepted contra-indications to MRI in the NHS. Although rare in neonates, this can include implanted medical devices that contain metal. We have qualified this as '…iii) Infants with a contraindication to MRI at 3Tesla determined by the Edinburgh Imaging safety policy, which is developed in accordance with UK Medicines and Healthcare Products Regulatory Agency (MHRA) safety guidelines.'

Finally, I have a personal question about the control group. Polluted environments are increasingly being described as risk factors for neurodevelopmental disorders. Do you think you will be able to control for this?

Response 8. Thank you for this suggestion. We collect neighbourhood-level information for deriving the deprivation index (Table 1), which is a covariate in planned analyses. This is useful in the present context because people who reside in more deprived areas are typically those exposed to higher pollutant levels (https://academic.oup.com/jpubhealth/article/39/3/485/3076806). Beyond using deprivation as a proxy, it is plausible for us to link neighbourhood-level geographical data to environmental pollutant data, and we will certainly explore this possibility.

In conclusion, the research question is highly relevant with a strong protocol. Conclusions could help to redefine the goals of care for preterm infants and have a significant impact on outcomes.

Response 9. Thank you.

Reviewer: 2
Dr. Birgit Arabin, Humboldt University of Berlin
Comments to the Author:
GENERAL OVERVIEW, ORIGINALITY
The present paper summarizes an extensive multidisciplinary research protocol on the mechanisms and causal evidence of neurodevelopmental and cognitive impairment associated with early prematurity. The design is an exposure-based prospective cohort study. Another advantage is that data from the UK National Neonatal Research Database and the National Pupil Database can be linked.
Preterm birth (PTB) is not only a syndrome with many causes, even the pre-and postnatal consequences of prematurity for the developing brain may have different pathways which are important to modify neurocognitive development by specific interventions. Therefore, this project intends to cover the knowledge gap in how far harmful effects up to adulthood are due to changes in maternal and fetal, respectively neonatal cortisol, social factors or the interrelated brain metabolism and brain morphology. PTB is the leading cause of perinatal and infant mortality, accounting for approximately one-third of newborn deaths. Among survivors, short-term complications and the risk for long-term neurodevelopmental (but also cardiovascular, immunological and metabolic) diseases are globally increased. Therefore, this project is of elementary importance possibly interrupting vicious circles of social and global inequality,

Response 10. Thank you for commenting on the importance and implications of the study.

STEPWISE APPROACH
ABSTRACT: The abstract clearly describes the research goals – but according to the editorial advice the authors should clearly explain the dates when the study starts (or has been started). Similarly, it is unclear from the abstract at what postnatal age certain examinations from the neonates, respectively from the infants are collected. The authors should add this information.

Response 11. Thank you. We have now added study dates to the abstract (see response 3). We have also added the following sentence to the abstract 'Infant biosamples and MRI will be collected between birth and 44 weeks gestational age.'

STRENGTHS AND LIMITATIONS: Accurately described – the limitation that the project is restricted to high-income settings is understandable but leaves us with uncertainties in how far this can be of relevance for low/medium-income settings.

Response 12. We agree. The point is included in the list of strengths and limitations in the original manuscript.

INTRODUCTION/BACKGROUND: Clear
RATIONAL FOR STUDY. The authors discuss that it is not necessarily prematurity per se harming the development, anatomy and function of brain structures but associated factors of prematurity such as maternal stress or systemic inflammation already acquired pre- or postnatally. Several measurements will be performed such as glucocorticoids in maternal and neonatal hair or indirect determination of the activity of 11-ß dehydrogenase or DNA methylation. It might also be an option to determine pro-inflammatory markers and even the telomere length in the maternal and umbilical blood (see Lazarides C, et al. Maternal pro-inflammatory state during pregnancy and newborn leukocyte telomere length: A prospective investigation. Brain Behav Immun. 2019;80:419–26 or Verner G, et al. Maternal psychological resilience during pregnancy and newborn telomere length: a prospective study. Am J Psychiatry. 2021;178(2):183–92)

Response 13. We agree that there are other plausible pathways linking PTB with atypical brain development, including changes in the proteome and telomere biology. While these are currently outside the scope of funded work, we have future-proofed the study by collecting biological material for storage in the event of future funded analyses (e.g., dried blood spots for immunoregulatory protein measurement). Furthermore, the data access and collaboration policy signposted in the manuscript describes the governance framework for external researchers to access study resources.

METHODS: The authors describe their methods in detail by text and Table 1. However, it is not clear at what postnatal age the neuroimaging is scheduled. This might depend on feasibility due to neonatal health. Nevertheless, there should be some rough description.

Response 14. MRI will take place at 38-44 weeks; we have now added this to Table 1. Please also see Response 11.

Did the authors also consider prenatal neuroimaging (see: Wu Y, et al. Association of prenatal maternal psychological distress with fetal brain growth, metabolism, and cortical maturation. JAMA Netw Open. 2020;3(1):e1919940)? It has been demonstrated that maternal stress (frequent in women admitted due to threatening labor, the size of several parts of the fetal brain and brain metabolism are all reduced. It might be interesting to compare the results before and after delivery.

Response 15. We agree this is an interesting idea but fetal brain MRI is outside the scope of the funded study.

CONCLUSION
The study protocol is original, well-written and deserves publication. The content of the research is essential. Nevertheless, the timing of some examinations, mainly neuroimaging, gut microbiome etc. and the follow-up of these children after the neonatal period should be clarified more clearly.

Response 16. Thank you for this positive feedback. We have added the timing of all neonatal biosample collections and MRI acquisition to Table 1. The study is not currently funded beyond the neonatal period, however, with additional funding follow-up studies may take place. This will certainly be a consideration for us as the the present phase of data collection nears completion.

Reviewer: 1
Competing interests of Reviewer: None

Reviewer: 2
Competing interests of Reviewer: None
